# Recovering nutrients and unblocking the cake layer of an electrochemical anaerobic membrane bioreactor

**DOI:** 10.1038/s41467-024-53341-y

**Published:** 2024-10-22

**Authors:** Yuhan Zhang, Yongbin Wang, Zhibin Chen, Chengzhi Hu, Jiuhui Qu

**Affiliations:** 1 State Key Laboratory of Environmental Aquatic Chemistry, Research Center for Eco-Environmental Sciences, Chinese Academy of Sciences, 100085 Beijing, China; 2 University of Chinese Academy of Sciences, 100049 Beijing, China

**Keywords:** Bioanalytical chemistry, Environmental chemistry

## Abstract

The sustainable development strategy shifts water treatment from pollution removal to resource recovery. Here, an electrochemical resource-recovery anaerobic membrane bioreactor (eRAnMBR) that employed a magnesium plate and conductive membrane as dual anodes is presented and shows excellent performance in carbon, nitrogen, and phosphorus recovery, as well as 95% membrane anti-fouling. The Mg^2+^ released alters the physicochemical properties of sludge, unblocking the cake layer, and recovers ammonium and phosphate, yielding 60.64% purity and 0.08 g d^−1^ struvite deposited onto cathode to be separated from sludge. The enhanced direct interspecies electron transfer, along with hydrogen evolution and alkalinity increase due to the electrochemical reactions, significantly increase methane yield and purity (93.97%) of the eRAnMBR. This increased internal energy can cover the additional electricity and electrode consumption. This integrated eRAnMBR reactor boasts the benefits of short process, low maintenance, and low carbon footprint, introducing a concept for the next generation of wastewater treatment.

## Abstract (web summary)

Recovering resources from wastewater sources is an important sustainable development strategy. Here, authors build an electrochemical resource-recovery anaerobic membrane bioreactor for the simultaneous full recovery of carbon, nitrogen, and phosphorus, while demonstrating membrane fouling mitigation.

## Introduction

In the context of sustainable development, sewage treatment facilities are not only required to recover renewable resources but also achieve carbon emission reduction. Anaerobic membrane bioreactors (AnMBRs), which integrate anaerobic processes with membrane separation, are among the most effective low-carbon wastewater treatment technologies, offering excellent application prospects in sustainable water treatment. However, challenges such as membrane fouling^1,2^ and the inability to recover nitrogen and phosphorus^3,4^ significantly restrict the large-scale application of AnMBRs in wastewater treatment^5^. Addressing these issues could greatly enhance their efficiency and application scope^6^. Besides, biogas produced by anaerobic digestion processes plays a key role in the emerging market for renewable energy^7^. However, in addition to 60–70% methane (CH_4_), raw biogas contains 30–40% carbon dioxide (CO_2_) which decreases its specific calorific value^8^. Therefore, the ratio of CH_4_ to CO_2_ needs to be increased for biogas to work as an effective fuel source^9^.

Numerous methods have been employed to resist the membrane fouling^10^. Recently, an approach that incorporates an electric field in AnMBRs has garnered considerable attention as a chemical-free and efficient way to alleviate in-situ membrane pollution^11,12^. The electrostatic repulsion at the cathode effectively inhibited the deposition of negatively charged extracellular polymeric substances (EPS) and soluble microbial products (SMP), which are the main substances contributing to membrane fouling. Most studies have focused on the electrophoresis, electrostatic forces, and other electrodynamic effects of the electric field on the membrane fouling^11–13^, while research on the mechanisms of the regulation of the cake layer in AnMBRs is relatively scarce. However, altering the physicochemical properties of the sludge can directly influence the substances that form membrane fouling, thus modifying the structure of the cake layer or slowing its formation.

As for nitrogen and phosphorus removal, struvite precipitation is considered an excellent method, with recovered struvite further serving as a slow-release fertilizer^14^. Among the various struvite precipitation methods, electrochemical processes are considered promising due to their high efficiency in nitrogen and phosphorus removal^15,16^, coupled with the advantages of simple operation, controllable reactions, and reduced sludge production^17,18^. Typically, struvite precipitation is used as a post-treatment process following biological treatment^15,16^, and its application within the bioreactor is rare which faces the problem of the separation of struvite and sludge. If magnesium (Mg) anode was introduced into the AnMBR system, it could exert dual effects of the nitrogen and phosphorus removal and electrochemical regulation on membrane fouling. Additionally, the Mg^2+^ ions and hydroxide ions (OH^−^) produced from the hydrogen evolution reaction at the cathode can absorb CO_2_ produced by anaerobic digestion, synergizing CO_2_ mineralization and biogas upgrading^19^. Understanding how the Mg anode impacts the biological system during this process is a key area that warrants further investigation.

In this study, an electrochemical resource-recovery AnMBR (eRAnMBR) equipped with double anodes, consisting of an Mg plate and conductive membrane-anode, was developed to facilitate nutrient and energy recovery, as well as alleviate membrane fouling by unblocking the cake layer. Over a 200-day operational period, the eRAnMBR enhanced the recovery of carbon, nitrogen, and phosphorus and improved membrane filtration capacity. The introduction of the Mg anode improved struvite generation and enhanced the absorption of CO_2_ from the biogas into the sludge-water mixture, while also altering the structure of the cake layer and inhibiting the formation of the gel layer. The introduction of the electric field enhanced the microbial anodic oxidation and accelerated the transformation of proteins within the system. Changes in the archaea community and the increased capacity for direct interspecies electron transfer (DIET) within the system were also the reason for the promotion of CH_4_ production. This study presents a strategy for resource and energy recovery from sewage, demonstrating considerable potential for efficient and eco-friendly wastewater treatment.

## Results

### Carbon, nitrogen, and phosphorus recovery in the eRAnMBR

**Fig. 1:**
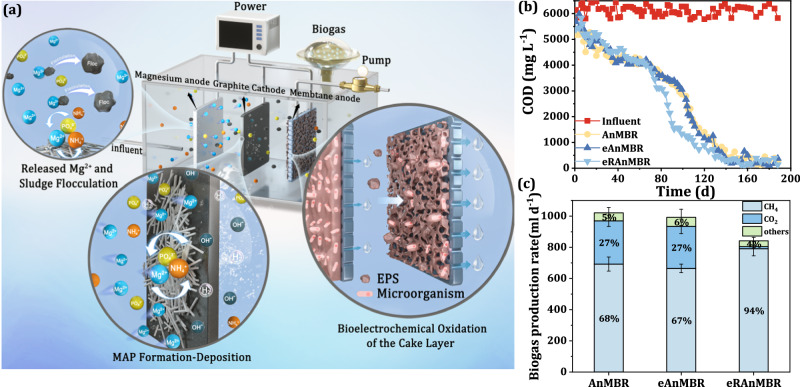
Overview of the mechanism and carbon recovery in the eRAnMBR. **a** Mechanisms of the eRAnMBR for struvite recovery and membrane antifouling. **b** Variations in COD in the three reactors of AnMBRs during the operation period. **c** Biogas production rate and biogas composition in the three reactors of AnMBRs during the stable operation period, the percentages of each gas have been rounded to the nearest whole number in the figure (The figure primarily highlights the production rates of the main components of biogas, CH_4_ and CO_2_. In addition to these two, other gases in biogas we measured were provided in Supplementary Fig. 7). Error bars represent the standard deviation of the biogas production measured 9–12 times during the stable operation phase of each reactor. The anodes of the eAnMBR comprised a conductive membrane and a graphite plate, while the anodes of the eRAnMBR included a conductive membrane and a Mg plate. Applied voltage of both the eRAnMBR and eAnMBR was 0.6 V. COD concentration in the influent was 6 g L^−1^. (Note: the word electro-AnMBR means we mention both the eRAnMBR and eAnMBR). Source data are provided as a Source Data file.

The eRAnMBR configuration and processes at the anodes and cathode are detailed in Fig. 1a. Initially, the variations in chemical oxygen demand (COD) an CH_4_ across the three AnMBRs were measured to assess carbon recovery. As shown in Fig. 1b, during the 200 days of operation, the COD concentration gradually decreased and stabilized, with all three reactors demonstrating excellent degradation of organic contaminants, achieving a COD removal rate of 95%. In the first 40 days, the COD degradation rate increased rapidly, but between days 40 and 70, the rate of improvement hardly increased. Therefore, the hydraulic retention time (HRT) was extended from 6 days to 8 days to enhance acclimation efficiency at day 70. After this adjustment, a faster increase in the COD degradation rate of the sludge in the eRAnMBR was observed. This is because, compared to the AnMBR and the eAnMBR, the electrode reactions in the eRAnMBR were more intense (It was concluded from the pH data in Supplementary Fig. 2b and the current data in Supplementary Figs. 3,  4). The alkalinity produced by the cathodic hydrogen evolution reaction could prevent acid inhibition of the system in the initial stages of the anaerobic process (Supplementary Fig. 5), allowing it to complete the four stages of anaerobic process earlier^20^. From Fig. 1c, the eRAnMBR also exhibited superior carbon recovery performance, with CH_4_ production rate reaching approximately 790.9 mL d^−1^, higher than the other two groups, which produced about 692.8 mL d^−1^. Furthermore, in the eRAnMBR, the proportion of CO_2_ in the biogas decreased from 27.18% to 1.63%, and the proportion of CH_4_ in the biogas increased from 67.82% to 93.97%, which was considerably higher than that of previously published reports of AnMBRs^10,21,22^. This improvement aided the upgrade to biomethane which can be recovered as natural gas with less treatment^9,23^. This enhancement in CH_4_ production efficiency was likely due to the significant release of Mg^2+^ by the Mg anode and increase in alkalinity, which improved the solubility of CO_2_ in the liquid phase^7^ and its absorption from the biogas. This is evidenced by the significant increase in bicarbonate ion (HCO_3_^−^) concentration observed following the addition of the Mg plate (Supplementary Fig. 6), accelerating CO_2_ transfer in the liquid phase and facilitating its utilization by methanogens for CH_4_ production^19^. Additionally, the hydrogen (H_2_) produced was utilized by hydrogenotrophic methanogens, in conjunction with CO_2_ for CH_4_ production^24^.

**Fig. 2:**
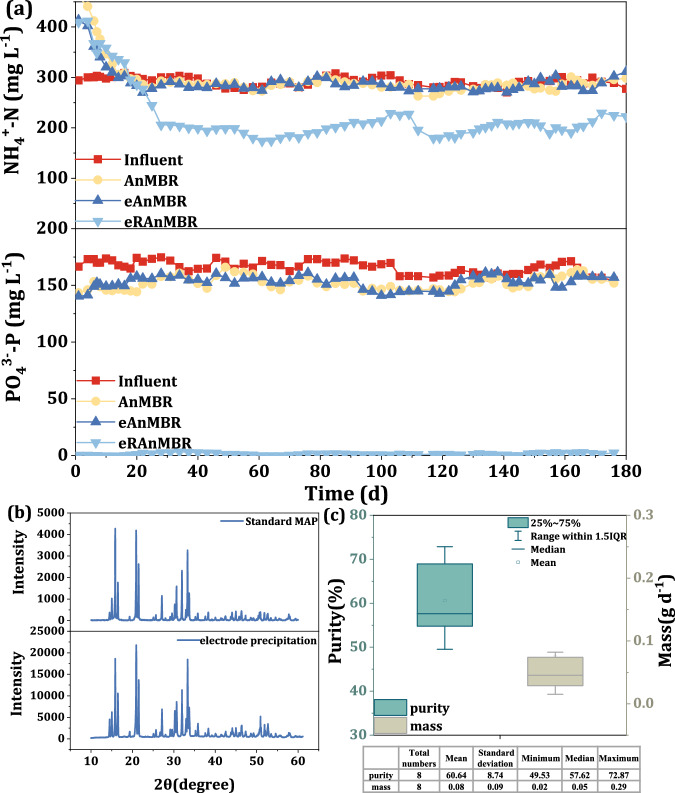
Nitrogen, and phosphorus recovery in the eRAnMBR. **a** Variations in NH_4_^+^-N and PO_4_^3-^-P of the three AnMBRs. The anodes of the eAnMBR comprised a conductive membrane and a graphite plate, while the anodes of the eRAnMBR included a conductive membrane and a Mg plate. Applied voltage of both the eRAnMBR and eAnMBR was 0.6 V. (Note: the word electro-AnMBR means we mention both the eRAnMBR and eAnMBR). NH_4_^+^-N and PO_4_^3-^-P concentrations in influent were 300 mg L^−1^ and 160 mg L^−1^, respectively. **b** X-ray Diffraction spectra of precipitates deposited on the cathode. **c** Mass and purity of struvite deposited on the cathode plate during the stable period, and the elements (total numbers, mean, standard deviation, minimum, median, maximum) of this box-plot were showed in the form of a table below the figure. Source data are provided as a Source Data file.

The eRAnMBR also demonstrated considerable nitrogen and phosphorus resource recovery. As shown in Fig. 2a, ammonium (NH_4_^+^-N) and phosphate (PO_4_^3−^-P) significantly decreased in the eRAnMBR, with removal rates of 30.59% and 99%, respectively, compared to minimal recovery in the controlled AnMBR and the eAnMBR. In addition to the sacrificial anode reaction induced by a 0.6 V bias applied to the Mg anode, the Mg anode and graphite cathode also formed a galvanic cell when a 0.6 V bias was applied to the membrane anode, leading to the continuous release of Mg²⁺ (Supplementary Fig. 4). Additionally, in the presence of microorganisms, non-Faradaic current was generated, which further accelerated the corrosion of Mg (Supplementary Fig. 8 and Supplementary Fig. 9). These processes ensured that sufficient Mg²⁺ was available to meet the requirements for struvite formation, resulting in a phosphate removal rate of 99% through the precipitation of magnesium phosphate and struvite. The removed nitrogen and phosphorus were recovered as struvite, which partially precipitated on the cathode surface and was separated from the sludge. When the current between the Mg anode and the graphite cathode dropped below 0.1 A (referred to the current when the 0.6 V applied on the Mg plate, current data was displayed in Supplementary Fig. 4), both the Mg anode and graphite cathode were replaced, which prevented the deposits on the electrodes from affecting mass transfer and electron exchange as well as ensured sufficient Mg^2+^ throughout the experiment. The images of Scanning electron microscope (SEM) revealed that the cathode plate surface was covered with crystals displaying the needle-shaped prismatic morphology typical of struvite (Supplementary Fig. 10a). The X-ray Diffraction (XRD) results (Fig. 2b) indicated that the precipitates formed on the cathode surface displayed peaks similar to those of struvite, indicating its generation and deposition on the cathode. This process was facilitated by the release of Mg^2+^ from the sacrificial Mg anode during electrolysis, and hydrogen evolution on the graphite cathode surface, which increased localized pH^25,26^ and promoted struvite formation. The Mg^2+^ ions generated at the anode then migrated towards the cathode, driven by the electric field force, thus promoting struvite precipitation on the cathode surface^27^ (Fig. 1a). Calculating through the method of determination of the struvite on the cathode surface, the purity of struvite was approximately 60.64% and the mass was 0.08 g d^−1^, which meant approximately 9.04% struvite generated in the reactor was deposited on the graphite cathode surface (Supplementary Fig. 11).

### Antifouling performance and optimization of cake layer structure

**Fig. 3:**
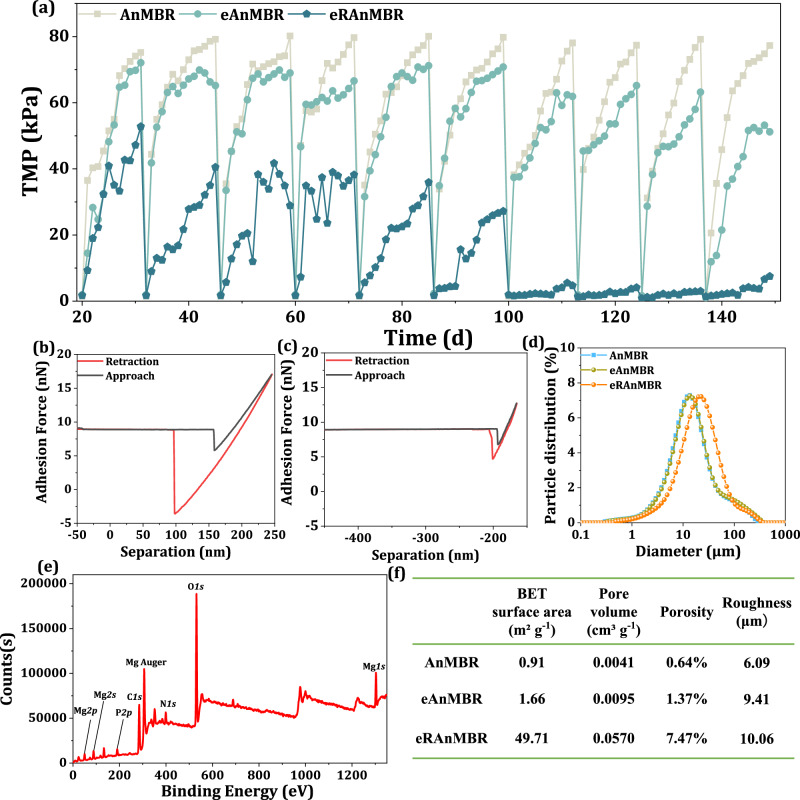
Optimization of cake layer structure and its role in membrane antifouling. **a** Antifouling performance of the three reactors. When TMP of one group reached 80 kPa, the membrane module of all the three reactors were renewed together and the next phase of the operation began. **b**, **c** Adhesion force measurements by AFM between metal surface and sludge flocs of the eAnMBR and the eRAnMBR. **d** Particle size distribution of sludge in the three reactors. **e** XPS spectra of sludge in the eRAnMBR. **f** BET surface area, pore volume, porosity, and roughness across three reactors. Source data are provided as a Source Data file.

Membrane fouling was also significantly alleviated in the eRAnMBR. As illustrated in Fig. 3a, under identical effluent flux conditions, the transmembrane pressure (TMP) of the AnMBR reached approximately 80 kPa, while the TMP of the eAnMBR and the eRAnMBR were approximately 64 kPa and 4 kPa, respectively. Membrane fouling was reduced by 95% in the eRAnMBR, substantially extending the life span of the membrane, indicating the key role on membrane fouling mitigation of Mg anode.

First, the presence of Mg^2+^ decreased the viscosity of the sludge (Supplementary Fig. 12b), potentially weakening the adsorption force between the membrane surface and sludge flocs, as highly viscous sludge flocs tend to accumulate on the membrane surface^28^. This effect was confirmed by the images of Atomic force microscopy (AFM)^29^, which showed a decrease in adhesion force from 13.15 nN to 4.94 nN, thereby slowing the formation of the cake layer^30^. Second, the size of the sludge flocs increased in the eRAnMBR (Fig. 3d), and X-ray photoelectron spectroscopy (XPS) analysis confirmed the existence of Mg(OH)_2_ (Fig. 3e, Supplementary Note 2 and Supplementary Fig. 18). Mg hydroxide coagulation primarily occurred through charge neutralization and adsorptive mechanisms, leading to floc formation^31^. The decrease in absolute zeta potential of sludge (Supplementary Fig. 12a) in the eRAnMBR further confirmed the effect of Mg^2+^ on sludge floc agglomeration. Third, Mg^2+^ changed the secondary structure of EPS protein weakened the formation of gel layer. As a typical negatively charged and sticky substance^4^, the contribution of proteins to membrane fouling in the AnMBRs was more than that of polysaccharides in EPS. In comparison to the AnMBR and the eAnMBR, EPS proteins of the eRAnMBR exhibited fewer α-helices and more β-sheets, contributing to the high aggregation capability of the sludge^32,33^ (Supplementary Fig. 13). A lower α-helix/ (β-sheet + random coil) ratio also suggested that the proteins in the eRAnMBR possessed less hydrogen bonds^34–36^ which played a key role in the gel layer^37,38^, thus weakening the formation of a compact foulants layer.

Consequently, the eRAnMBR cake layer exhibited a larger BET specific surface area and total pore volume, with porosity reaching 7.47% which was 10 times higher than that of the control group (Fig. 3f). The released Mg^2+^ improved the physicochemical properties of sludge flocs making which difficult to deposit or adhere to the membrane surface, thus forming a loose porous cake layer structure, which greatly improved the service life of the membrane.

### Microbial anodic oxidation and its effect on fouling distribution

**Fig. 4:**
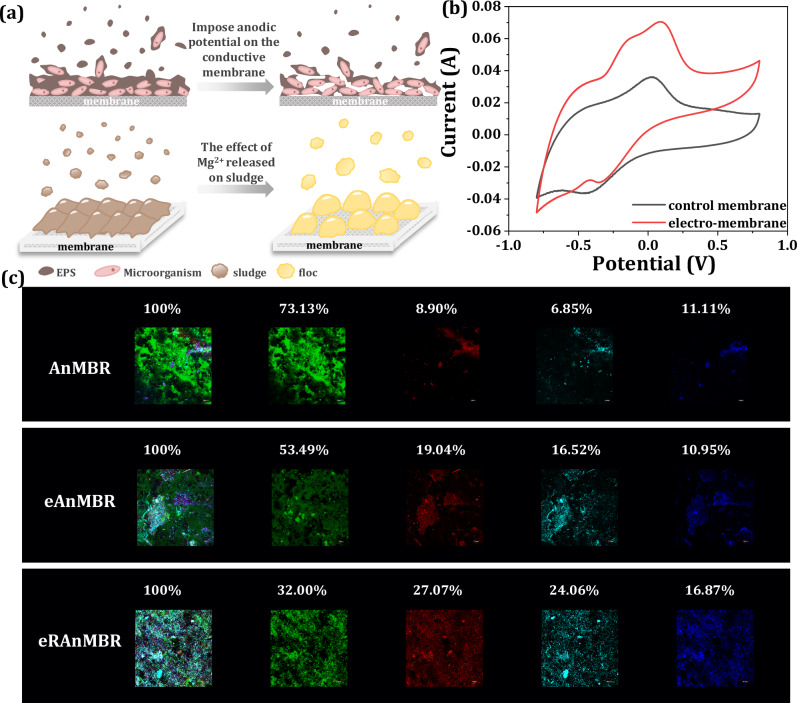
Electrochemical response of the biofilm on the membrane-anode surface. **a** Membrane fouling mitigation of the eRAnMBR: effects of anode potential on the cake layer and Mg^2+^ released on sludge. **b** Cyclic voltametric analyses of control membrane and electro-membrane in the 0.8 to −0.8 V range at scan rates of 2 mV s^−1^ using a three-electrode system, with Ag/AgCl (R0303, 6 mm × 65 mm) as the reference electrode, conductive membrane as the working electrode, and graphite cathode as the opposing electrode. Control membrane was the membrane of the AnMBR, and electro-membrane was the membrane of the eAnMBR. **c** CLSM images of membrane surface of three reactors (total cells are in red, proteins are in green, α-polysaccharides are in purple, and β-polysaccharides are in blue). The percentage represented the ratio of the fluorescence intensity of each substance to the total fluorescence intensity. Source data are provided as a Source Data file.

To investigate the mitigation mechanisms of membrane fouling following the application of anodic potential to the membrane, fouling distribution of the cake layer and electrochemical performance were further explored. The Confocal laser-scanning microscopy (CLSM) images showed that while protein intensity was very high in the AnMBR, it significantly decreased after the application of anodic potential imposed on the membrane (Fig. 4c). In contrast, total cell intensity increased from 8.90% to 19.04% following the imposition of anodic potential on the membrane, indicating the formation of electro-biofilm. The images also revealed that proteins in the AnMBR tightly and thickly covered the membrane surface, whereas proteins in the membrane-anode showed a much looser structure, facilitating water permeation. Consequently, the roughness of the cake layer increased after the anodic potential was applied on the membrane (Supplementary Fig. 14a–c).

The mechanisms behind the aforementioned results were further investigated. The cyclic voltammogram (CV) curves in Fig. 4b showed that the sludge cake layer on the membrane-anode exhibited markedly higher electron-storage capacity and peak oxidation current compared to the control membrane, indicating the enhancement of microbial anodic oxidation. And the cytochrome c-type coding genes associated with bioelectrochemical oxidation^39^ of microorganisms on the membrane surface also increased by 14%, from 299 on the control membrane of AnMBR to 341 on the electro-membrane of the eAnMBR (Supplementary Table 1), especially the ccm proteins involved in post-translational modifications essential for Mtr pathway protein production^40^. Therefore, the current density between the anode and cathode increased driven by exoelectrogens (Supplementary Fig. 3a)^41^. These results confirmed that microbial anodic oxidation was strengthened after the imposition of anodic potential on the membrane, conductive to the degradation of proteins in the cake layer of the membrane-anode^42,43^. Given the higher contribution of proteins to membrane fouling in AnMBRs than polysaccharides^4^, the degradation of proteins was more beneficial for the mitigation of membrane fouling.

**Fig. 5:**
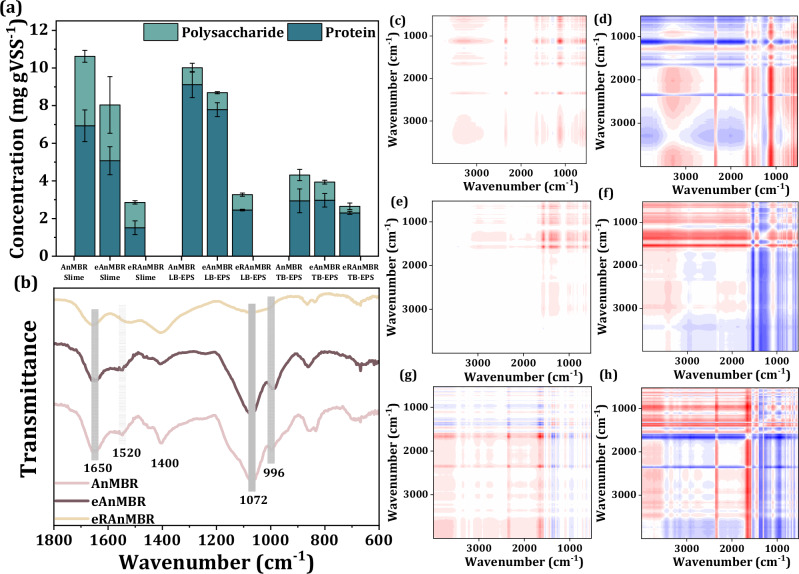
Electrochemical response of EPS. **a** Proteins and polysaccharides concentration of slime layer, LB-EPS layer, and TB-EPS layer of sludge around the membrane in the three reactors during the stable period. Error bars represent the standard deviation of the EPS concentration results, which were measured after being extracted three times from the sludge of each reactor. **b** Fourier transform infrared spectroscopy (FTIR) spectra of sludge EPS in the three reactors during the stable period. (Here, EPS referred to the overall EPS obtained using a one-step extraction method, without layered extraction, including both LB-EPS and TB-EPS). The gray blocks were used to highlight the peaks in the figure, with the corresponding wavelengths of each peak written below the gray blocks, and the functional groups corresponding to each peak were explained in detail in the Supplementary Note 1. Synchronous spectra (**c**, **e**, **g**) and asynchronous spectra (**d**, **f**, **h**) of 2D-COS analysis using LB-EPS and SMP infrared spectra of sludge around the membrane in the three reactors, with (**c**) and (**d**) showing the AnMBR, (**e**) and (**f**) showing the eAnMBR, and (**g**) and (**h**) showing the eRAnMBR. Red and blue represent positive and negative correlations, respectively. Relevance increases with increasing color intensity. Source data are provided as a Source Data file.

Moreover, the quantity of slime, loosely-bound EPS (LB-EPS), tightly-bound EPS (TB-EPS) of sludge around the membrane in the three reactors was also calculated to further investigate the effect of electric field on organic matter forming membrane fouling. Results indicated that levels of all the three EPS layers decreased following the application of anodic potential to the membrane-anode, with a significant reduction in the eRAnMBR (Fig. 5a and Supplementary Fig. 15). Two-Dimensional correlation spectroscopy (2D-COS) analysis were generated using LB-EPS and SMP infrared spectra (Fig. 5c–h) to further gain insights into the molecular compositions of organic substances. During the transformation from LB-EPS to SMP, signal changes in the asynchronous spectra revealed that the sequence of electron disturbance in the control group was polysaccharide (1150 cm^−1^) > amide I (1640 cm^−1^) > amide II (1540 cm^−1^). In contrast, for the electro-AnMBR groups, the sequence of electron disturbance was amide II (1540 cm^−1^) > amide I (1640 cm^−1^) > polysaccharide (1150 cm^−1^). The intricate structure, diverse functional groups, and uneven surface charge made proteins more easily influenced by the electric field than polysaccharides^39,44^. Additionally, redox proteins, as the main electroactive components in EPS, played a crucial role in the extracellular electron transfer process^45^. These made proteins more likely to participate in redox reactions after the electric field applied, thereby accelerating the electron transport rate.

### Microbial community succession and its enhancement of CH_4_ production

**Fig. 6:**
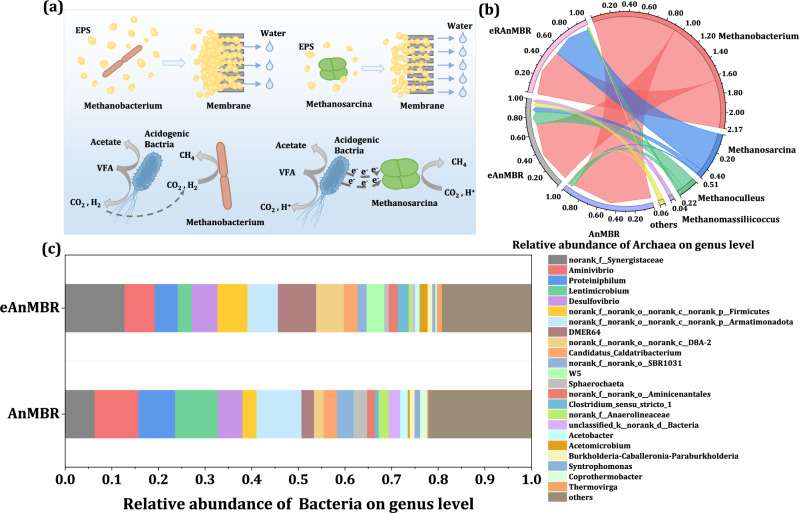
Microbial community succession in the AnMBRs. **a** Differences of Methanobacterium and Methanosarcina on EPS secretion and electron transport. Methanobacterium was the only dominant archaea in the AnMBR and eAnMBR, while Methanosarcina was also the dominant archaea in the eRAnMBR. **b** Relative abundance of archaea at genus level in the AnMBR, eAnMBR, and eRAnMBR. **c** Relative abundance of bacteria at genus level in the AnMBR and eAnMBR. The anodes of the eAnMBR comprised a conductive membrane and a graphite plate, while the anodes of the eRAnMBR included a conductive membrane and a Mg plate. Applied voltage of both the eRAnMBR and eAnMBR was 0.6 V. (Note: the word electro-AnMBR means we mention both the eRAnMBR and eAnMBR). Source data are provided as a Source Data file.

The analysis of archaeal community also explained the mechanism behind enhanced CH_4_ production of the eRAnMBR. From Fig. 6b we can see that Methanobacterium was the dominant archaea genus in the control AnMBR, with a relative abundance of 92.9%. In contrast, following the application of an electric field, the relative abundance of Methanosarcina increased to 5.6% in the eAnMBR and 43.9% in the eRAnMBR. The abundance of Methanosarcina increased in the eAnMBR was favorable for CH_4_ production^46^, due to its capability to produce CH_4_ through all the three methanogenesis pathways (acetoclastic, hydrogenotrophic, and methylotrophic methanogenesis)^22,47^. As shown in Supplementary Fig. 16, all relative abundances of modules involved in methanogenesis increased in the eRAnMBR. Besides, Methanosaeta and Methanosarcina, the only methanogen genera with membrane-bound cytochromes^48^, may facilitate extracellular electron exchange and DIET process^49^. The abundance of DMER64 genus possessing DIET ability and the cytochrome c-type coding genes related to DIET also increased (Fig. 6c and Supplementary Table 2). And the apparent electron transfer rate constant (k_app_) of the eRAnMBR was higher than the other two reactors (Supplementary Fig. 17), indicating that the overall extracellular electron transfer performance was strengthened^50^. DIET, considered to be a more efficient electron transfer pathway^51^ without relying on the diffusion of electron carriers such as H_2_, was conducive to the increase of CH_4_ yield in the eRAnMBR. In addition, Methanosarcina produced less EPS than Methanobacterium^52^ which can help alleviating membrane fouling.

## Discussion

To strengthen energy and resource recovery as well as membrane anti-fouling, a eRAnMBR featuring dual anodes—an Mg anode and a membrane-anode—was developed. Notably, the eRAnMBR system outperformed traditional AnMBRs, removing 30.59% of NH_4_^+^-N and 99% of PO_4_^3−^-P, while producing struvite with 60.64% purity and depositing 0.08 g d^−1^ on the cathode surface for easy separation from the sludge. The eRAnMBR system also upgraded biogas to biomethane, significantly increasing its calorific value and facilitating its recovery as natural gas. The operating cost analysis indicated that the average energy production capacity of the eRAnMBR, exceeded the additional energy and electrode material consumption, demonstrating the economic viability of this configuration (Supplementary Note 3). The enhancement of DIET and all methanogenesis pathways within the system, along with the H_2_ evolution and alkalinity production after the application of the Mg anode, all contributed to the increase in CH_4_ purity and yield. In-depth research on the physicochemical properties of the sludge explained the 95% reduction in membrane fouling of the eRAnMBR. The released Mg^2+^ increased floc size, decreased the adhesion force between the sludge particles and the membrane surface, and reduced the proportion of hydrogen bonds in the secondary structure of EPS proteins, thereby weakening the formation of the gel layer and making the cake layer structure loose and porous. The application of the electric field also enhanced the microbial anodic oxidation, and made proteins more likely to participate in redox reactions.

In this study, it was found that alleviating membrane fouling by regulating the cake layer through altering the physicochemical properties of the sludge was far more effective than the singular electrodynamic effect. This provided a new approach for extending membrane lifespan in future applications. The new configuration, which switched between the membrane and Mg anodes, reduced Mg material input while effectively recovering three types of resources, enabling the application of AnMBRs in a more sustainable wastewater treatment. As for industrial applications, more economical anode materials, electrode configurations that highly facilitate mass transfer and struvite precipitation, and more efficient utilization of the generated CH_4_ to reduce costs are engineering considerations for the practical application of this reactor.

## Methods

### AnMBR construction and operation

In this study, three integrated AnMBRs, eRAnMBR, eAnMBR, and a conventional AnMBR, were operated in parallel. Each bioreactor system consisted of a continuously stirred cylindrical anaerobic digester with an effective volume of 5.0 L (190 mm internal diameter, 240 mm height), into which the membrane unit was directly immersed. A removable module was designed at the center of the reactor’s top lid, where the electrode plates and membrane modules were fixed. This setup allowed for quick replacement of the membrane modules and electrode plates without disrupting the anaerobic environment (Supplementary Fig. 1). The three setups were operated under different conditions.In the eRAnMBR, a conductive Ti membrane (141.4 cm^2^ surface area and 0.22 μm pore size, purity >99.6%) was used as one of the anodes, while a Mg plate (100 mm × 60 mm × 3.5 mm, 99.9%, 1.74 g cm^−3^) and a graphite plate (100 mm × 60 mm × 3.5 mm, 99.9%, 1.8 g cm^−3^) acted as the second anode and the cathode, respectively. In the eAnMBR and conventional AnMBR systems, the Mg plate was replaced with a graphite anode. Copper wiring was used to connect the electrodes in both the eRAnMBR and eAnMBR to a 0.6 V DC power source, with the anodes operating at an optimized ratio of 1:60 for Mg and the Ti membrane in the eRAnMBR (the same ratio for graphite and Ti membrane in the eAnMBR). In contrast, the conventional AnMBR operated with an open circuit, serving as the control group. To maintain homogeneity of the mixed liquor, a magnetic stirrer was used in all reactors, and the liquid levels were kept constant via a float valve system connected to a balance tank. Biogas production was measured by periodically replacing the gas collection bags positioned atop the reactors. Membrane fouling was monitored by tracking the evolution of TMP under constant flux conditions. When the TMP reached a threshold of 80 kPa, the membrane module was replaced, and a new operational phase commenced. In addition, pH and oxidation-reduction potential sensors were installed within the reactors for continuous monitoring, ensuring the system remained stable. All reactors were operated at a hydraulic retention time (HRT) of 8 days, with the temperature controlled at 38 ± 1 °C using a thermostatic water bath.

### Experimental wastewater and sludge

The AnMBR was fed with highly concentrated organic wastewater, rich in nitrogen and phosphorus, simulated using glucose (99%, SCR), CH_3_COONa (99%, SCR), KH_2_PO_4_ (99.5%, SCR), NH_4_Cl (99.8%, SCR), and tryptone (BR, SCR) as sources of carbon, phosphorus, and nitrogen. The COD, NH_4_^+^-N, and PO_4_^3−^-P levels in raw water were approximately 6.0 ± 0.25 g L^−1^, 300 mg L^−1^, and 160 mg L^−1^, respectively. Trace element formulae referred to previous research and detailed compositions are shown below: MgSO_4_ (99%, SCR) 3.0 g L^−1^; MnSO_4_·H_2_O (99%, SCR) 0.5 g L^−1^; NaCl (99.8%, SCR) 1.0 g L^−1^; FeSO_4_·7H_2_O (99%, SCR) 0.1 g L^−1^; CaCl_2_·2H_2_O (96%, SCR) 0.1 g L^−1^; CoCl_2_·H_2_O (99%, SCR) 0.1 g L^−1^; ZnCl_2_ (98%, Macklin) 0.13 g L^−1^; CuSO_4_·5H_2_O (99%, SCR) 0.01 g L^−1^; AlK(SO_4_)_2_·12H_2_O (99.8%, SCR) 0.01 g L^−1^; H_3_BO_3_ (99.5%, Innochem) 0.01 g L^−1^; Na_2_MoO_4_ (99%, SCR) 0.025 g L^−1^; NiCl_2_·6H_2_O (98%, SCR) 0.024 g L^−1^; Na_2_WO_4_·2H_2_O (99%, SCR) 0.025 g L^−1^. The inoculated activated sludge in the reactor was obtained from the sludge digestion tank of the Gaobeidian Wastewater Treatment Plant (Beijing, China). Initial anaerobic sludge was added into the reactors through a 1-mm mesh sieve after dilution so that the mixed liquor suspended solids (MLSS) and mixed liquor volatile suspended solids (MLVSS) in the reactor eventually reached 10,000 mg L^−1^ and 5000 mg L^−1^, respectively.

### Physicochemical characterization of anaerobic digestion

COD was measured using a HACH DR/3900 spectrophotometer (CO 80539; HACH, USA). The concentrations of NH_4_^+^-N and PO_4_^3−^-P were measured using Nessler’s reagent and molybdate spectrophotometry (T6, PERSEE, China). The TMP was detected using a pressure sensor between the effluent and pump (Thunder Magnet, Shanghai Instrument Co., China) and recorded using a data acquisition device. A pH detector (HQ30d, Hach Co., USA) was used to determine pH.

EPS were extracted by thermal extraction (Supplementary Method 1)^53^. All extracted EPS samples were measured using a three-dimensional excitation emission matrix (EEM) (F-4600, HITACHI, Japan) and FTIR (Nicolet iN10MX, Thermo Fisher Scientific, USA). Imported the infrared data the infrared spectra data of LB-EPS and SMP into the software 2D Shige ver. 1.3 (Shigeaki Morita, Kwansei-Gakuin University, Japan), and then obtained the 2D-COS spectra to gain insights into the molecular compositions and formation mechanisms of organic substances. The amide I region of the FTIR spectra at 1700–1600 cm^−1^ was then obtained using Omnic v8.0 (Thermo-Fisher Scientific Inc.) to explore protein secondary structures. The second-derivative and deconvolution spectra were obtained using Peakfit v4.12 (Seasolve Software Inc.). The amide I band was then fitted until the residual reached the minimum according to the maximum absorption intensity, band frequency, and bandwidth from the second-derivative spectra. Peakfit software was used for quantitative analysis of each peak^54^. The EPS concentration was measured, with protein content determined using the Folin-phenol method and polysaccharide content determined using the phenol-sulfuric acid method.

A laser particle size analyzer (Mastersizer2000, Malvern, UK) was used to measure particle size of the sludge. A Zetasizer Nano Z instrument (Malvern, UK) was used to determine the zeta potential of the sludge and a Viscosity meter (DV2TLV, Brookfield, USA) was used to determine sludge viscosity. The sludge from the AnMBRs was freeze-dried for 48 h using a vacuum freeze dryer (FD-IA-50, Biocool, China), then examined using XPS (ESCALAB250Xi, Thermo Fisher Scientific, USA) and BET surface area analysis (ASAP 2460, Micromeritics, USA). AFM was employed to measure the interaction forces between metal surface and sludge flocs. A true color confocal microscope (Zeiss CSM700, Germany) was used to observe the surface morphology of the cake layer. CV curves of the membranes in the AnMBRs were constructed using a three-electrode system (CHI660, Chenhua Instrument, China), in which Ag/AgCl (R0303, 6 mm × 65 mm) served as the reference electrode, membrane served as the working electrode, and graphite cathode served as the opposing electrode with the range of 0.8 V to −0.8 V and scan rates of 2 mV s^−1^ (electrode parameters were shown in AnMBR construction and operation). CLSM (Fluoview FV-1000, Olympus, Germany) was employed to visualize the distribution of nucleic acids, proteins, and polysaccharides on the sludge cake layers.

### Determination of struvite

At the end of each operational cycle, the electrode plate of the eRAnMBR was replaced, and the precipitate accumulated on the surface of the graphite cathode was scraped off. The precipitate was then imaged and analyzed using SEM (Nova Nano SEM 450, FEI, USA) in conjunction with XRD (X’Pert3 Powder, PANalytical, Netherlands). Given that most common impurities formed during struvite crystallization, such as Mg(OH)_2_, MgHPO_4_, Mg_3_(PO_4_)_2_, and MgKPO_4_^55^, do not contain nitrogen, we assumed that one mole of ammonium corresponded to one mole of struvite^56^ for purity calculations. The struvite purity was determined using Eq. (1):

1 $${{{\rm{Purity}}}}=\frac{{({{{{\rm{NH}}}}}_{4}^{+}{\mbox{-}}{{{\rm{N}}}})}_{{{{\rm{prec}}}}}}{{({{{{\rm{NH}}}}}_{4}^{+}{\mbox{-}}{{{\rm{N}}}})}_{{{{\rm{struv}}}}}}=\frac{{({{{{\rm{NH}}}}}_{4}^{+}{\mbox{-}}{{{\rm{N}}}})}_{{{{\rm{prec}}}}}}{57}$$

where (NH_4_^+^-N)_prec_ is the measured concentration of the ammonium nitrogen in the precipitate and (NH_4_^+^-N)_struv_ is the theoretical content of nitrogen in pure struvite (57 mg g^−1^). To quantify the struvite purity, 0.1 g of precipitate from the plate was dissolved in 0.5% nitric acid solution, and the total volume was brought up to 50 mL using ultrapure water. The ammonium concentration was then measured using Nessler’s Reagent Spectrophotometry. Based on the measured ammonium content, the mass of struvite in the precipitate was calculated, and the struvite purity was obtained by dividing this value by the initial 0.1 g sample^57^.

### Microbial community analysis

The sludge of the AnMBRs was collected at the end of the experiment and cryopreserved at −80 °C. The bacterial and archaeal communities were analyzed using 16S rRNA high-throughput sequencing, with an E.N.Z.A.® soil DNA kit (Omega Bio-tek, Norcross, GA, America) used to extract DNA from sludge samples before sequencing. According to the instructions of the kit, the basic extraction steps included the cracking of microbial cells, removal of impurities such as proteins, and DNA precipitation and purification. The DNA integrity of the samples was then examined by agarose gel. High-throughput sequencing of the extracted DNA was conducted using the Majorbio sequencing platform (Shanghai, China). The hypervariable region V3-V4 of the bacterial 16S rRNA gene were amplified with primer pairs 338F (5’-ACTCCTACGGGAGGCAGCAG-3’) and 806R (5’-GGACTACHVGGGTWTCTAAT-3’)^58^. The hypervariable region V4-V5 of the archaeal 16S rRNA gene was amplified with primer pairs 524F10extF (5’-TGYCAGCCGCCGCGGTAA-3’) and Arch958RmodR (5’- YCCGGCGTTGAVTCCAATT-3’)^52^. The amplifications were carried out using T100 Thermal Cycler PCR thermocycler (BIO-RAD, USA). Illumina Miseq-PE300 sequencing service was provided by Majorbio (Shanghai, China).

### Metagenomic analysis

The cake layers of the membranes in the AnMBR and the eAnMBR and anaerobic sludge samples of bulk solution in the three groups were collected for metagenomic analysis from the perspective of microbes and genes. Analysis included DNA extraction, library construction, metagenomic sequencing, sequence quality control, genome assembly, gene prediction, taxonomic annotation, and alignment of functional protein coding genes. BLASTP v2.2.28+ (http://blast.ncbi.nlm.nih.gov/Blast.cgi) was used to compare non-redundant gene sequences with the Kyoto Encyclopedia of Genes and Genomes (KEGG) database, with the expected value of BLAST comparison parameters set to 1e^−5^. Based on the comparative results, functional annotation was conducted using KOBAS 2.0 [4] (KEGG Orthology Based Annotation System). The abundance of genes corresponding to KO, Pathway, EC, and Module was summed to calculate the abundance of this functional category.

### Reporting summary

Further information on research design is available in the Nature Portfolio Reporting Summary linked to this article.

## Supplementary information

Supplementary Information

Reporting Summary

## Source data

Source Data

Transparent Peer Review file

## Data Availability

The data supporting the findings of this work are available within the article and its Supplementary Information files. Source data are provided with this paper. The number PRJNA1164645 gives access to raw data deposited in the NCBI Sequence Read Archive database. Source data are provided with this paper.
