# Supplementary material for: Recovering nutrients and unblocking the cake layer of an electrochemical anaerobic membrane bioreactor
Source: Nat Commun. 2024 Oct 22;15:9111. doi: 10.1038/s41467-024-53341-y (PMC11496669; doi:10.1038/s41467-024-53341-y)
Supplement: Supplementary file 1 — Supplementary Information [file 41467_2024_53341_MOESM1_ESM.pdf]

Content of Supplementary material

Supplementary Notes

Supplementary Note 1 Analysis results of FTIR

Supplementary Note 2 Analysis results of XPS

Supplementary Note 3 Preliminary operating cost analysis for eRAnMBR

Supplementary Methods

Supplementary Method 1 The method of extracellular polymeric substances (EPS) extraction

Supplementary Figures

Supplementary Figure 1 The setup of eRAnMBR: (a) Schematic (b) Stereogram.

Supplementary Figure 2 Reactor performances along operation. Variation of (a) ORP and (b) pH of the three AnMBR reactors. The anodes of the eAnMBR comprised a conductive membrane and a graphite plate, while the anodes of the eRAnMBR included a conductive membrane and a Mg plate. Applied voltage of both the eRAnMBR and eAnMBR was 0.6 V. During the acclimation phase (before the COD degradation rate stabilized), if the system pH dropped below 6.5, we added appropriate amount of sodium bicarbonate ($NaHCO_3$) to prevent acid inhibition, which could lead to system failure. Source data are provided as a Source Data file.

Supplementary Figure 3 Change of current in the eAnMBR. (a) Change of current between the membrane anode and graphite cathode in the eAnMBR after the membrane module renewed. (b) The current between the graphite anode and graphite cathode when graphite is used as the anode in the eAnMBR, and the elements (total numbers, mean, standard deviation, minimum, median, maximum) of this box-plot were showed in the form of a table below the figure. Source data are provided as a Source Data file.

Supplementary Figure 4 The current between Mg anode and graphite cathode in the eRAnMBR. The switching time between Mg anode and membrane anode was 1 min:1 h. The dispersed points at high current were 0.6 V voltage applied on the Mg anode, while the densely connected points at low current were 0.6 V voltage applied on the

membrane anode. The figure showed the current changes at different times after the replacement of Mg anode and graphite cathode. (a) was earlier stage after the replacement of Mg anode and graphite cathode, (b) was middle stage after the replacement of Mg anode and graphite cathode, and (c) was the end stage after the replacement of Mg anode and graphite cathode. Source data are provided as a Source Data file.

Supplementary Figure 5 Volatile fatty acids (VFA) production in the AnMBR, eAnMBR, eRAnMBR, at days 74, 81, and 86 (All the days chosen were after the hydraulic retention time was adjusted from 6 days to 8 days). Source data are provided as a Source Data file.

Supplementary Figure 6 Comparison of HCO_3^- and CO_3^{2-} concentration of batch anaerobic fermentation experiments with or without the addition of Mg plate. Error bars represent the standard deviation of the results from three duplicate measurements of each independent experimental group. Source data are provided as a Source Data file.

Supplementary Figure 7 Content of other gases in biogas (biogas except for CH_4 and CO_2): (a) Concentration of H_2 and H_2S in biogas in the AnMBR, eAnMBR and eRAnMBR which showed differences during the stable operation period, and the elements (total numbers, mean, standard deviation, minimum, median, maximum) of this box-plot were showed in the form of a table below the figure. (b) Proportion of gases which were common in all the three reactors of AnMBRs. In this study, gas was collected using gas bags, making it impossible to avoid the escape of H_2[9-11]. As a result, data of H_2 were lower than the actual value, but it can still be used to compare differences between the three reactors. Source data are provided as a Source Data file.

Supplementary Figure 8 Variations of Mg^{2+} in the eRAnMBR during the stable operation period. The elements (total numbers, mean, standard deviation, minimum, median, maximum) of this box-plot were showed in the form of a table below the figure. Source data are provided as a Source Data file.

Supplementary Figure 9 Reasons for the generation of non-Faradaic current in the eRAnMBR. (a) The polarization curves of the Mg plate under microbial and non-microbial conditions on the third day. Three lines of each group represent the results of

three scans. (b) The cyclic polarization curves of the Mg plate under microbial and non-microbial conditions on the first day. (c) Concentration of Mg^{2+} released in the system under microbial and non-microbial conditions within 7 days. Error bars represent the standard deviation of the results from two duplicate measurements of each independent experimental group. (d) Corrosion current, linear polarization resistance, cathodic slope, anodic slope and corrosion potential of the Mg plate obtained from the polarization curves under microbial and non-microbial conditions on the third day. All data were based on the SBR reactor, with both microbial and non-microbial conditions. Mg (35 mm × 25 mm × 1 mm, 99.9%, 1.74 g cm^{-3}) was used as the anode, graphite (35 mm × 25 mm × 1 mm, 99.9%, 1.8 g cm^{-3}) as the cathode, and Ag/AgCl (R0303, 6 mm× 65 mm) as the reference electrode. No voltage was applied. The wastewater conditions were consistent with the AnMBRs. The polarization curves and Mg^{2+} were measured over 7 days, with the polarization curves from the third day selected as representative. Source data are provided as a Source Data file.

Supplementary Figure 10 The characterization of precipitate on the cathode surface of the eRAnMBR. (a) SEM image of the formed struvite on cathode surface (The measuring scale is 1 mm). EDS image of the precipitate on cathode surface (b) and the elementary composition and proportion of the precipitate on cathode surface (c), abs. error corresponded to Normalized mass.

Supplementary Figure 11 Mass balance of the phosphorus and nitrogen and the distribution of struvite in the eRAnMBR. Source data of mass balance refer to data of Figure 2a and Source data of distribution of struvite refer to data of Figure 2c.

Supplementary Figure 12 Physicochemical properties of sludge flocs in the AnMBRs. (a) Zeta potential of sludge around membrane in the three reactors. Error bars represent the standard deviation of the results from four duplicate measurements of each reactor. (b) Sludge viscosity in the three reactors. Error bars represent the standard deviation of the results from two duplicate measurements of each reactor. Source data are provided as a Source Data file.

Supplementary Figure 13 Secondary structure of EPS protein. Second-derivative resolution enhancement and curve-fitted amide I region (1700–1600 cm^{-1}) for EPS

proteins from the (a) AnMBR, (b) eAnMBR, and (c) eRAnMBR. The original infrared data was obtained from the FTIR spectrum, and then use Peakfit v4.12 (Seasolve Software Inc.) to obtain the second-derivative and deconvolution spectra. The amide I band was then fitted until the residual reached the minimum according to the maximum absorption intensity, band frequency, and bandwidth from the second-derivative spectra. Peakfit software was used for quantitative analysis of each peak. (d) Relative content of each secondary structure in EPS proteins for the AnMBR, eAnMBR, and eRAnMBR. Source data are provided as a Source Data file.

Supplementary Figure 14 Membrane surface morphology of the (a) AnMBR, (b) eAnMBR and (c) eRAnMBR.

Supplementary Figure 15 Three-dimensional excitation emission matrix (3D-EEM) fluorescence spectroscopy of slime layer, LB-EPS, TB-EPS of EPS from sludge in the AnMBR, eAnMBR, eRAnMBR. Source data are provided as a Source Data file.

Supplementary Figure 16 The relative abundance of modules involved in methanogenesis. Source data are provided as a Source Data file.

Supplementary Figure 17 Overall extracellular electron transfer performance of the three reactors. Cyclic voltammetry (CV) of sludge sampled from the (a) AnMBR, (b) eAnMBR, (c) eRAnMBR measured at scan rates of 60, 80, 100, 150 and 200 mV s^{-1}. (d) The apparent electron transfer rate constant (kapp, s^{-1}) of the sludge in the three reactors. CV curves were constructed using a three-electrode system (CHI660, Chenhua Instrument, China), in which Ag/AgCl (R0303, 6 mm× 65 mm) served as the reference electrode, a graphite plate (10 mm × 5 mm × 1 mm, 99.9%, 1.8g cm^{-3}) served as the working electrode, and a platinum sheet (10 mm × 5 mm × 1 mm, 99.99%, 21.45 g cm^{-3}) served as the opposing electrode. The sludge samples were taken from the three reactors during the stable operation period. Source data are provided as a Source Data file.

Supplementary Figure 18 XPS spectra of sludge in the eRAnMBR: (a) Mg *1s* (The data were analyzed using Xpspeak 4.1 software) and (b) Magnified view of the 40-100 eV binding energy of Figure 3e. Source data refer to data of Figure 3e.

Supplementary Tables

Supplementary Table 1 Cytochrome c gene counts of the microorganisms on the membrane surface in the AnMBR and the eAnMBR. Source data are provided as a Source Data file.

Supplementary Table 2 Cytochrome c gene counts of the microorganisms in sludge bulk solution of the AnMBR, eAnMBR and eRAnMBR. Source data are provided as a Source Data file.

Supplementary Note 1 Analysis results of FTIR

FTIR analysis was conducted to further identify the variations of the functional groups in EPS of the three groups. The regions between 1800–600 cm^{-1}, which exhibited the main information about the compositions and functionalities of EPS spectra, was investigated in detail. The peak 1650 cm^{-1} is the stretching vibration of C=O, C-N related to protein (amide I band), 1520 cm^{-1} is related to amide II band (that is, N-H bending vibration and C=N stretching vibration), and the peak at 1400 cm^{-1} is the typical peak of phenolic hydroxyl, COO^- and CH_3COO^-, the peak 1072 cm^{-1} is the stretching vibration of C-O, -OH related to polysaccharides and the peak 996 cm^{-1} is the stretching vibration of C-H bond.

Supplementary Note 2 Analysis results of XPS

Fig.3e and Supplementary Figure 18 showed the XPS image of sludge in eRAnMBR, the binding energy of 49.6 eV was correspond with Mg(*2p*), which meant the existence of $Mg(OH)_2$[1]. The binding energy of 88.1 eV, 235.3 eV and 1303.3 eV were correspond with and Mg(*2s*), Mg Auger and Mg(*1s*)[2]. The peak assigned to $Mg(OH)_2$ (1302.5 eV) was also observed in the subpeak of Mg(*1s*)[3]. The magnesium hydroxide colloidal particles with positively charged play the role of condensation nucleus, adsorbing a large number of negatively charged organic matter, thereby increasing the floc particle size[4].

Supplementary Note 3 Preliminary operating cost analysis for eAnRMBR

In this system, many sources of the electric energy consumption (e.g. the pump, the constant temperature water bath and the mixer) and operation costs (e.g.pipeline and membrane replacement) were consistent between the eRAnMBR and conventional AnMBR. Therefore, the additional operating cost of the eRAnMBR compared to conventional AnMBR was composed of energy consumption (electricity) and material consumption (electrode) which drove the electrochemical processes. It was estimated using the following equation.

$$C\text{=aENC+bELC} \tag{1}$$

Where, C refers to the additional operating cost, USD m^{-3}; ENC is the energy consumption, kWh m^{-3}; ELC is the electrode consumption, kg m^{-3}; a is the unit cost of electricity, i.e. 0.102 USD kWh^{-1} [5]; and b is the unit cost of the magnesium (Mg) electrode, i.e. 2.56 USD kg^{-1} (Because graphite plates are cheap and can be reused during the experiment, we only consider Mg electrode here).

In this system, although additional electrical energy is consumed, the internal methane produced is also a form of energy. Therefore, ENC was calculated as follows:

$$\text{ENC}=W_e-W_b \tag{2}$$

The electric energy consumption (W_e, Wh·m^{-3}) was calculated as follows:

$$W_e=\frac{UIt}{3600V}=\frac{UI}{3600Q} \tag{3}$$

Methane was the internal capacity of AnMBR. Under standard conditions, gas production (P_2, Nm^3) was calculated as follows:

$$\frac{P_1V_1}{T_1}=\frac{P_2V_2}{T_2} \tag{4}$$

Biogas energy (methane to electricity, W_b, Wh·m^{-3}) was calculated as follows:

$$W_b=\frac{\varepsilon qV_2t}{3600V}=\frac{\varepsilon qV_2}{3600Q} \tag{5}$$

where U is the applied potential (V), I is the current (A), t is the running time (h), Q is the flow ($m^3 \cdot h^{-1}$), P_1 is the working pressure (atm), V_1 is the working biogas production rate ($m^3 \cdot h^{-1}$), T_1 is 298 K (25 °C), P_2 is one standard atmospheric pressure (atm), V_2 is the standard gas production ($Nm^3 \cdot h^{-1}$), T_2 is 273 K (0 °C), q is the heat value of the biogas (J·Nm^{-3}), and ε (%) is the methane-to-electricity conversion efficiency

with a typical value of 13.779% via recent methane fuel cells[6].

While, the electrode consumption was determined following Faraday's Law[7] as expressed in Eq. (6).

$$\mathrm{ELC}=\frac{IMt}{n\mathrm{F}V} \tag{6}$$

Where, I is the applied current, A; t is the electrolysis time, h; V is the volume of treated effluent, m^3; M is the molecular weight of iron electrode, g mol^{-1}; n is the number of transferred electrons; and F is the Faraday constant, coulomb mol^{-1}.

Based on the calculation of the increased methane production by the eRAnMBR compared to the control AnMBR, as the additional internal energy of the eRAnMBR, the C value calculated is -19.4 USD m^{-3} which means the novel eRAnMBR is economical if the methane produced is properly utilized.

Supplementary Method 1 The method of extracellular polymeric substances (EPS) extraction

Extracellular polymeric substances (EPS) were extracted by thermal extraction[8]. In brief, 25 mL of sludge-water mixture collected from the AnMBRs was first centrifuged at 4 000 *g* and 4 ℃ for 5 min, with the collected supernatant representing the slime layer. The sludge remaining in the centrifuge tube was then added to 25 mL of 0.05% NaCl solution for re-suspension, sheared for 1 min using a vortex mixer, and centrifuged again at 4 000 *g* for 10 mins and 4 ℃, with the collected supernatant representing LB-EPS. Subsequently, 25 mL of 0.05% NaCl solution was added to re-suspend the sludge left in the centrifuge tube, followed by a water bath at 60 ℃ for 30 min and centrifugation at 4 000 *g* for 15 min and 4 ℃, with the collected supernatant representing TB-EPS.

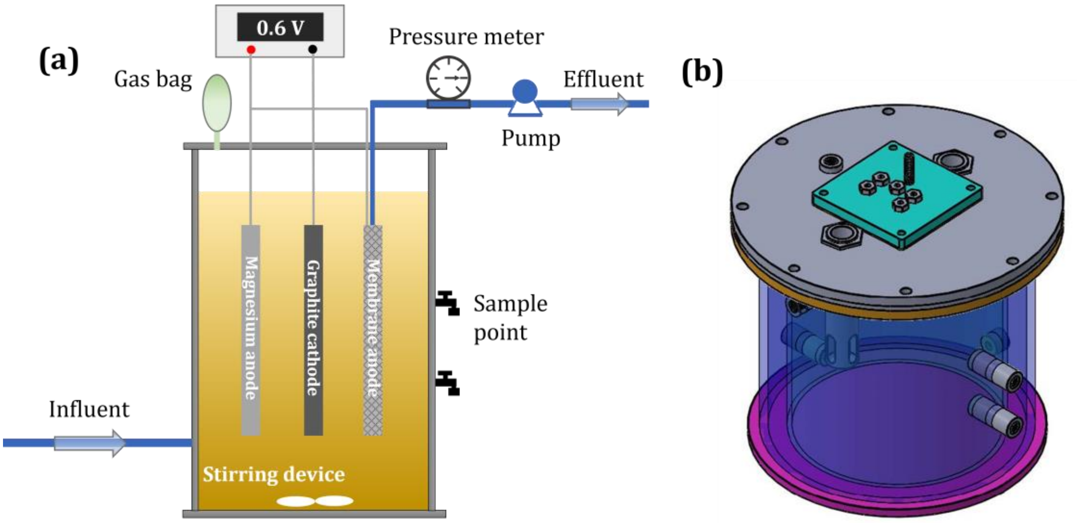

Supplementary Figure 1 The setup of eRAnMBR: (a) Schematic (b) Stereogram.

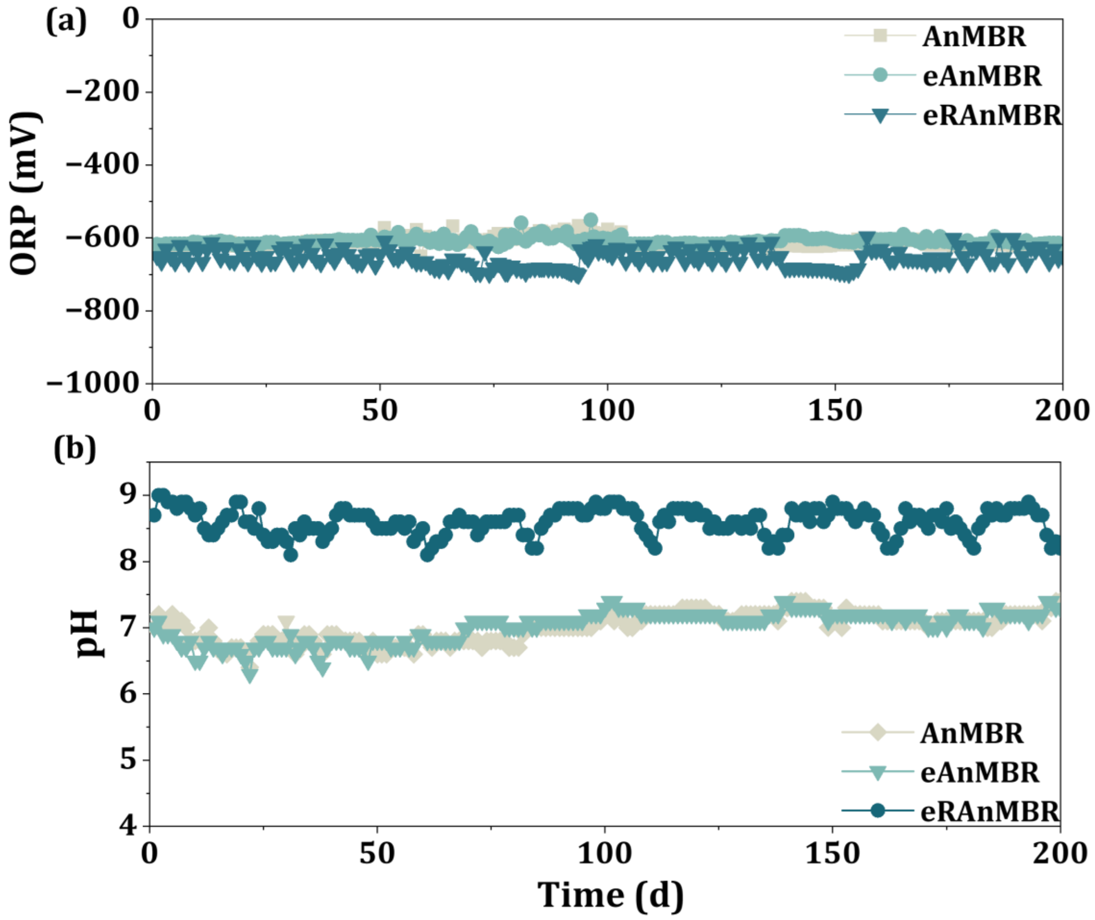

Supplementary Figure 2 Reactor performances along operation. Variation of (a) ORP and (b) pH of the three AnMBR reactors. The anodes of the eAnMBR comprised a conductive membrane and a graphite plate, while the anodes of the eRAnMBR included a conductive membrane and a Mg plate. Applied voltage of both the eRAnMBR and eAnMBR was 0.6 V. During the acclimation phase (before the COD degradation rate stabilized), if the system pH dropped below 6.5, we added appropriate amount of sodium bicarbonate ($NaHCO_3$) to prevent acid inhibition, which could lead to system failure. Source data are provided as a Source Data file.

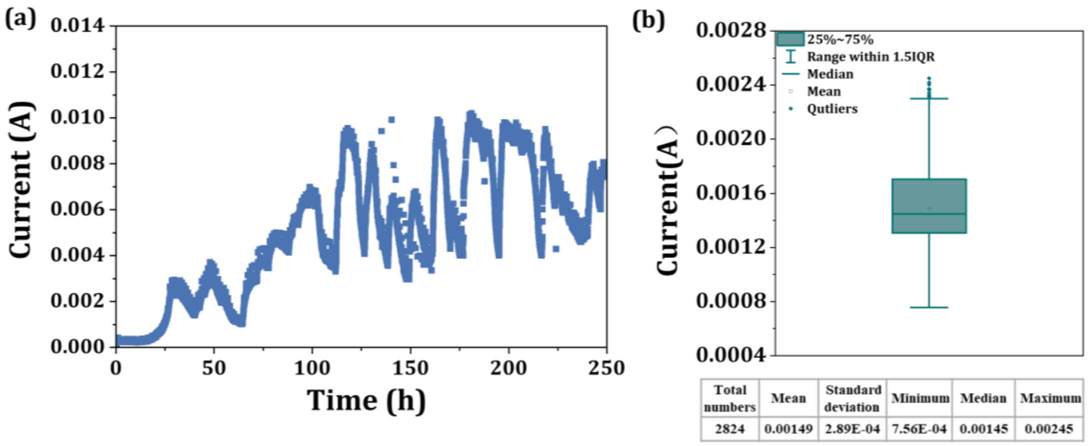

Total numbers	Mean	Standard deviation	Minimum	Median	Maximum
2824	0.00149	2.89E-04	7.56E-04	0.00145	0.00245

Supplementary Figure 3 Change of current in the eAnMBR. (a) Change of current between the membrane anode and graphite cathode in the eAnMBR after the membrane module renewed. (b) The current between the graphite anode and graphite cathode when graphite is used as the anode in the eAnMBR, and the elements (total numbers, mean, standard deviation, minimum, median, maximum) of this box-plot were showed in the form of a table below the figure. Source data are provided as a Source Data file.

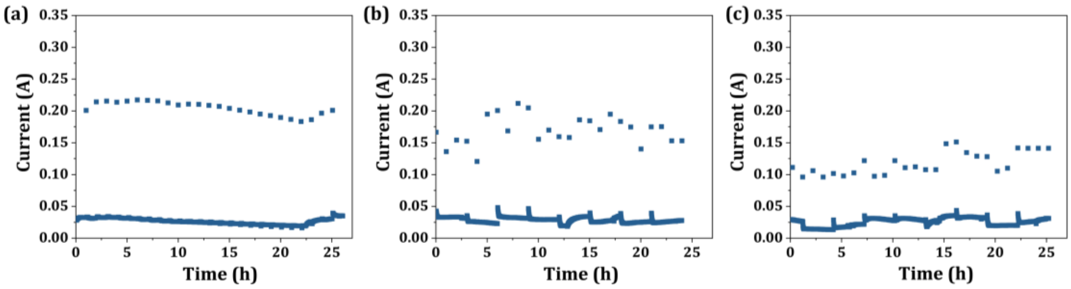

Supplementary Figure 4 The current between Mg anode and graphite cathode in the eRAnMBR. The switching time between Mg anode and membrane anode was 1 min:1 h. The dispersed points at high current were 0.6 V voltage applied on the Mg anode, while the densely connected points at low current were 0.6 V voltage applied on the membrane anode. The figure showed the current changes at different times after the replacement of Mg anode and graphite cathode. (a) was earlier stage after the replacement of Mg anode and graphite cathode, (b) was middle stage after the replacement of Mg anode and graphite cathode, and (c) was the end stage after the replacement of Mg anode and graphite cathode. Source data are provided as a Source Data file.

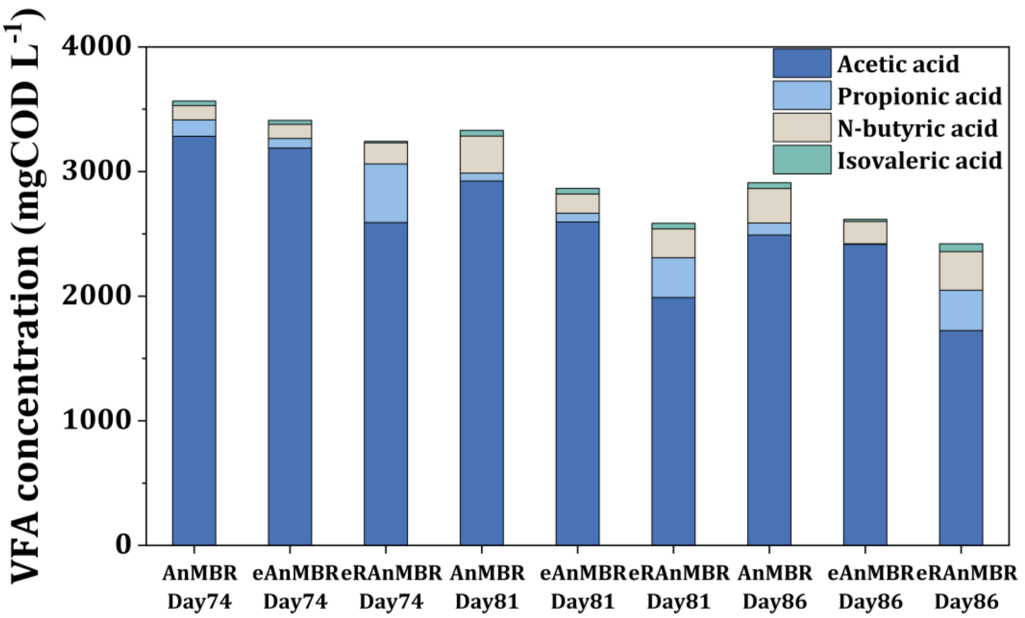

Supplementary Figure 5 Volatile fatty acids (VFA) production in the AnMBR, eAnMBR, eRAnMBR, at days 74, 81, and 86 (All the days chosen were after the hydraulic retention time was adjusted from 6 days to 8 days). Source data are provided as a Source Data file.

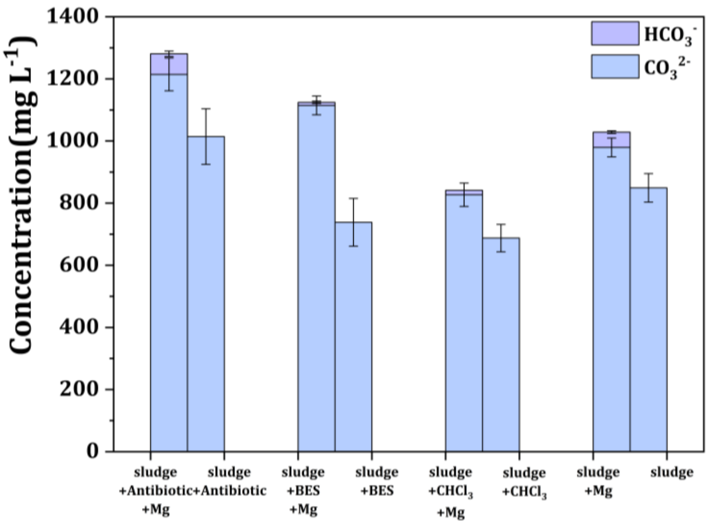

Supplementary Figure 6 Comparison of HCO_3^- and CO_3^{2-} concentration of batch anaerobic fermentation experiments with or without the addition of Mg plate. Error bars represent the standard deviation of the results from three duplicate measurements of each independent experimental group. Source data are provided as a Source Data file.

Batch experiments were conducted in 250 mL serum bottles. Anaerobic sludge was withdrawn from the AnMBR reactor, centrifugated at 6000 g for 5 min and washed three times by 0.05% NaCl. Sludge with the same concentration as the reactor and 2000 mg L^{-1} COD, 50 mg L^{-1} NH_4^+ and 10 mg L^{-1} PO_4^{3-} were mixed with the volume of 200 ml and then added to every serum bottles. 4 groups were set to selectively inhibit of bacteria, methanogens and both using antibiotics (200 mM), BES (100 mM) and $CHCl_3$ (200 mM) respectively. And there were 2 bottles in each group, one was added 0.114g Mg plate, one was added nothing. Specifically, the BES inhibits the Coenzyme M (CoM; $H_SCH_2CH_2SO_3^-$) which is a cofactor found in all methanogens, but not in other bacteria or archaea. CoM is involved in the terminal step of methane biosynthesis, where the methyl group carried by CoM is reduced to methane by methylCoM reductase. $CHCl_3$ can completely inhibit the acetoclastic and hydrogenotrophic methanogens and can decrease the activity of homoacetogenic bacteria and acetate-consuming sulfate-reducing bacteria. The antibiotics can inhibit bacteria but not methanogens[9].

From Supplementary Figure 6 we can see, whether the bacteria and methanogens were affected or not, HCO_3^- concentration increased after adding Mg plate, indicating that the absorption of CO_2 by the system was increased with the addition of Mg plate.

(a)

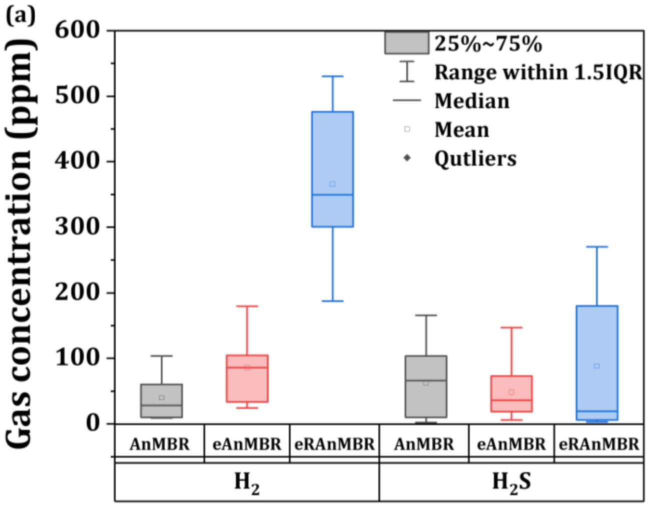

		Total numbers	Mean	Standard deviation	Minimum	Median	Maximum
H_2	AnMBR	6	40.01	37.10	9.00	28.16	103.72
	eAnMBR	6	85.70	56.14	24.58	85.76	179.70
	eRAnMBR	6	365.64	125.40	187.63	349.42	530.17
H_2S	AnMBR	25	62.51	52.11	2.24	66.44	165.84
	eAnMBR	16	48.83	41.24	6.10	36.03	146.76
	eRAnMBR	11	88.29	99.60	3.29	19.40	270.10

(b)

Gas	N_2	CO	C_2H_6	C_2H_4	C_3H_6	C_3H_8
Proportion	2.1831% ±0.2187%	0.0067% ±0.0028%	0	0	0	0

Supplementary Figure 7 Content of other gases in biogas (biogas except for CH_4 and CO_2): (a) Concentration of H_2 and H_2S in biogas in the AnMBR, eAnMBR and eRAnMBR which showed differences during the stable operation period, and the elements (total numbers, mean, standard deviation, minimum, median, maximum) of this box-plot were showed in the form of a table below the figure. (b) Proportion of gases which were common in all the three reactors of AnMBRs. In this study, gas was collected using gas bags, making it impossible to avoid the escape of H_2[10-12]. As a result, data of H_2 were lower than the actual value, but it can still be used to compare differences between the three reactors. Source data are provided as a Source Data file.

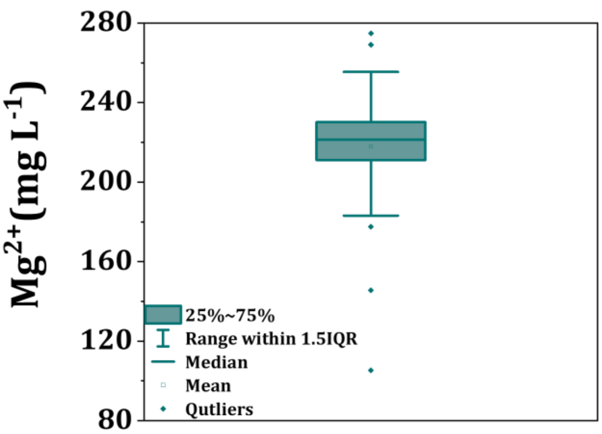

Total numbers	Mean	Standard deviation	Minimum	Median	Maximum
38	217.97	30.30	105.26	221.34	274.80

Supplementary Figure 8 Variations of Mg^{2+} in the eRAnMBR during the stable operation period. The elements (total numbers, mean, standard deviation, minimum, median, maximum) of this box-plot were showed in the form of a table below the figure. Source data are provided as a Source Data file.

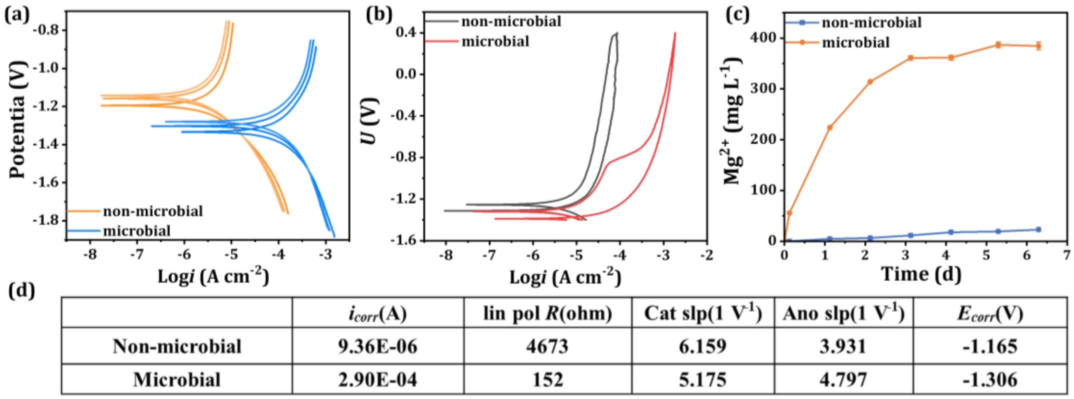

(d)

	i_{corr}(A)	lin pol R(ohm)	Cat slp(1 V^{-1})	Ano slp(1 V^{-1})	E_{corr}(V)
Non-microbial	9.36E-06	4673	6.159	3.931	-1.165
Microbial	2.90E-04	152	5.175	4.797	-1.306

Supplementary Figure 9 Reasons for the generation of non-Faradaic current in the eRAnMBR. (a) The polarization curves of the Mg plate under microbial and non-microbial conditions on the third day. Three lines of each group represent the results of three scans. (b) The cyclic polarization curves of the Mg plate under microbial and non-microbial conditions on the first day. (c) Concentration of Mg^{2+} released in the system under microbial and non-microbial conditions within 7 days. Error bars represent the standard deviation of the results from two duplicate measurements of each independent experimental group. (d) Corrosion current, linear polarization resistance, cathodic slope, anodic slope and corrosion potential of the Mg plate obtained from the polarization curves under microbial and non-microbial conditions on the third day. All data were based on the SBR reactor, with both microbial and non-microbial conditions. Mg (35 mm × 25 mm × 1 mm, 99.9%, 1.74 g cm^{-3}) was used as the anode, graphite (35 mm × 25 mm × 1 mm, 99.9%, 1.8 g cm^{-3}) as the cathode, and Ag/AgCl (R0303, 6 mm× 65 mm) as the reference electrode. No voltage was applied. The wastewater conditions were consistent with the AnMBRs. The polarization curves and Mg^{2+} were measured over 7 days, with the polarization curves from the third day selected as representative. Source data are provided as a Source Data file.

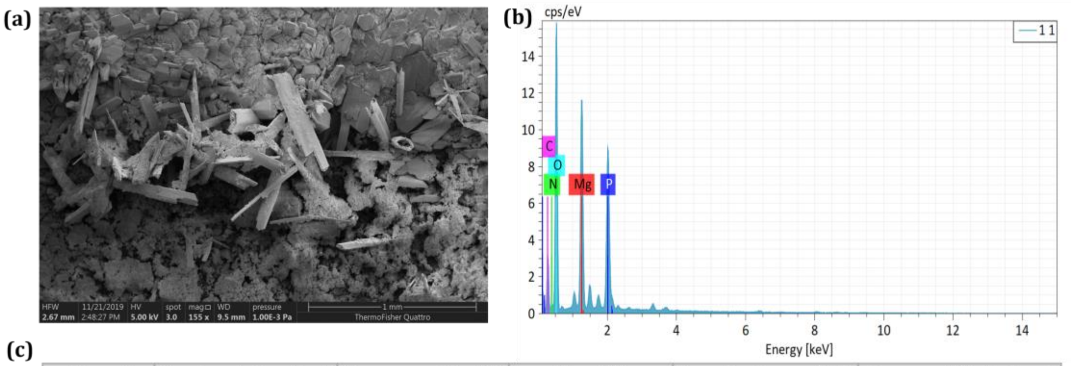

(c)

Element	Number of atoms(a.u.)	Atom proportion (%)	Mass (%)	Normalized mass （%）	abs. error (%) (1 sigma)
O	8	52.65	43.58	51.32	5.17
C	6	28.25	17.55	2067	2.53
Mg	12	8.56	10.76	12.67	0.60
P	15	6.14	9.84	11.58	0.40
N	7	4.40	3.19	3.76	0.71
		100.00	84.92	100.00	

Supplementary Figure 10 The characterization of precipitate on the cathode surface of the eRAnMBR. (a) SEM image of the formed struvite on cathode surface (The measuring scale is 1 mm). EDS image of the precipitate on cathode surface (b) and the elementary composition and proportion of the precipitate on cathode surface (c), abs. error corresponded to Normalized mass.

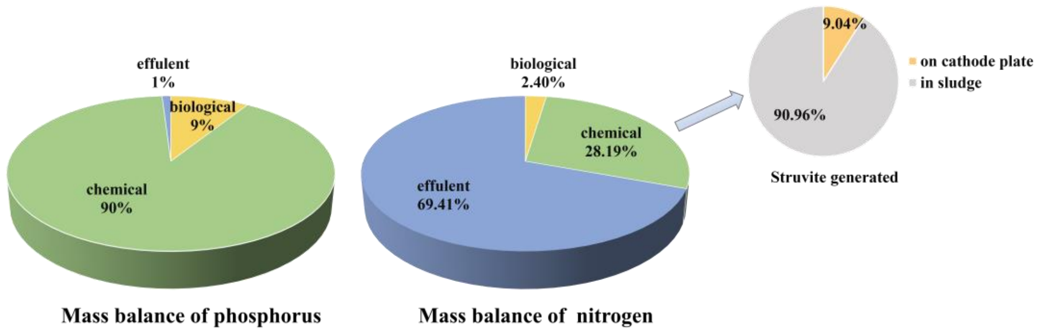

Supplementary Figure 11 Mass balance of the phosphorus and nitrogen and the distribution of struvite in the eRAnMBR. Source data of mass balance refer to data of Figure 2a and Source data of distribution of struvite refer to data of Figure 2c.

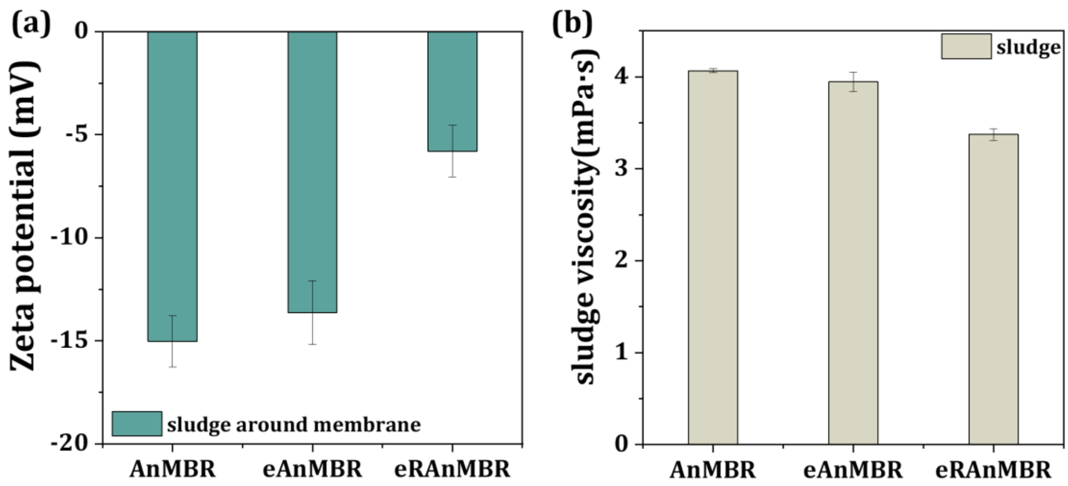

Supplementary Figure 12 Physicochemical properties of sludge flocs in the AnMBRs. (a) Zeta potential of sludge around membrane in the three reactors. Error bars represent the standard deviation of the results from four duplicate measurements of each reactor. (b) Sludge viscosity in the three reactors. Error bars represent the standard deviation of the results from two duplicate measurements of each reactor. Source data are provided as a Source Data file.

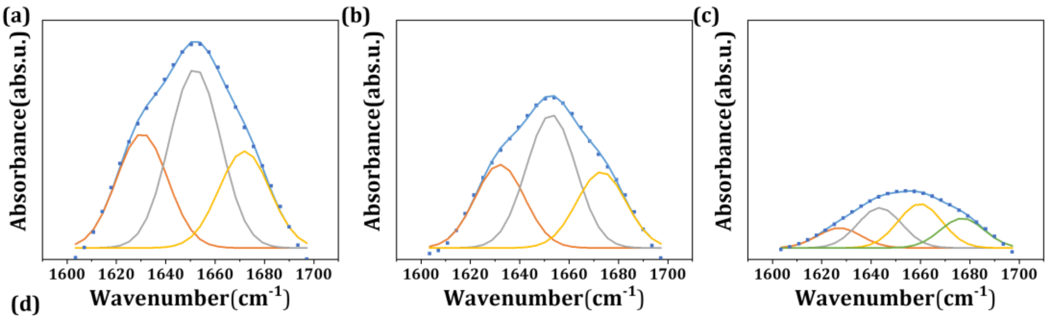

(d)

Sludge	α-helix	β-sheet	Random coil	β-turn	α-Helix/(β-sheet + random coil)
AnMBR	45.56%	28.49%	0.00%	25.96%	1.60
eAnMBR	45.89%	29.30%	0.00%	24.80%	1.57
eRAnMBR	32.97%	14.95%	29.96%	22.13%	0.73

Supplementary Figure 13 Secondary structure of EPS protein. Second-derivative resolution enhancement and curve-fitted amide I region (1700–1600 cm^{-1}) for EPS proteins from the (a) AnMBR, (b) eAnMBR, and (c) eRAnMBR. The original infrared data was obtained from the FTIR spectrum, and then use Peakfit v4.12 (Seasolve Software Inc.) to obtain the second-derivative and deconvolution spectra. The amide I band was then fitted until the residual reached the minimum according to the maximum absorption intensity, band frequency, and bandwidth from the second-derivative spectra. Peakfit software was used for quantitative analysis of each peak. (d) Relative content of each secondary structure in EPS proteins for the AnMBR, eAnMBR, and eRAnMBR. Source data are provided as a Source Data file.

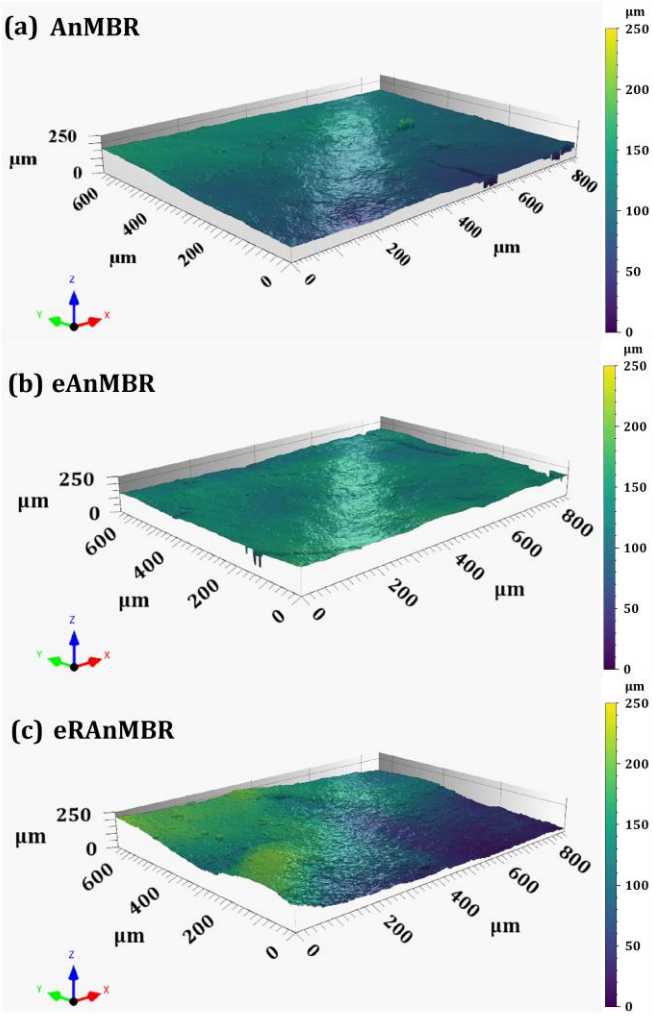

Supplementary Figure 14 Membrane surface morphology of the (a) AnMBR, (b) eAnMBR and (c) eRAnMBR.

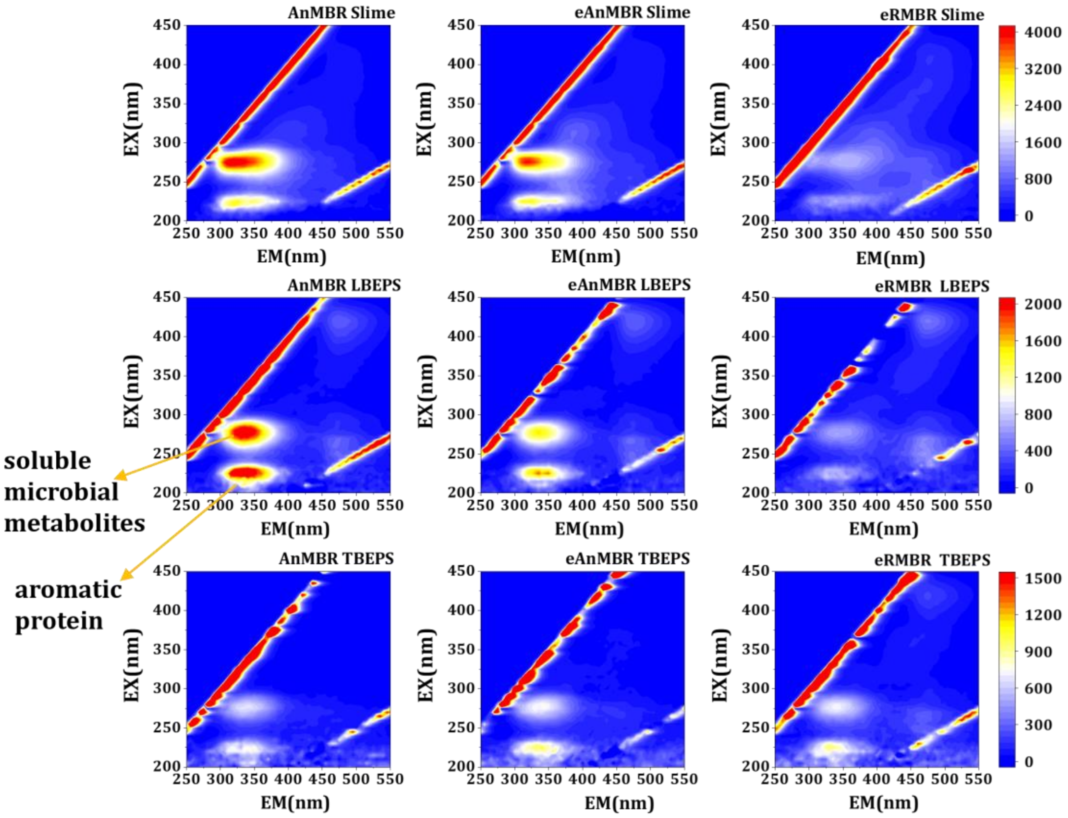

Supplementary Figure 15 Three-dimensional excitation emission matrix (3D-EEM) fluorescence spectroscopy of slime layer, LB-EPS, TB-EPS of EPS from sludge in the AnMBR, eAnMBR, eRAnMBR. Source data are provided as a Source Data file.

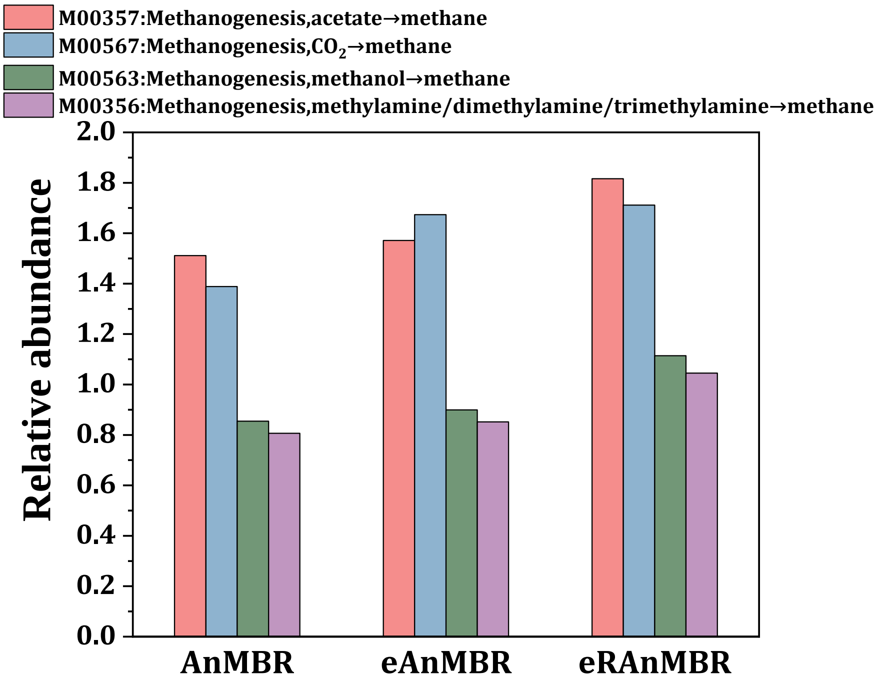

Supplementary Figure 16 The relative abundance of modules involved in methanogenesis. Source data are provided as a Source Data file.

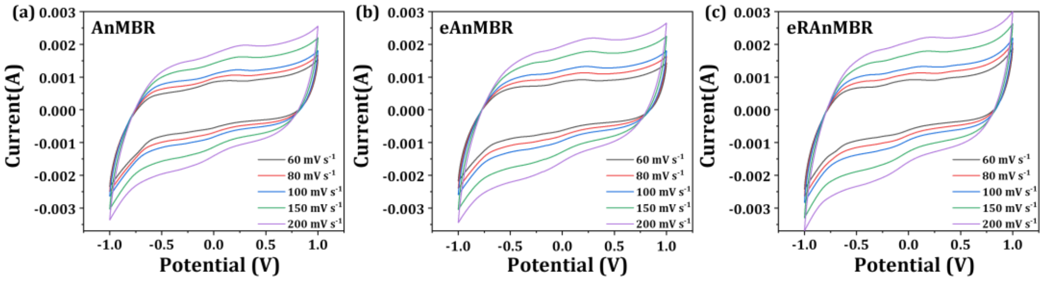

(d)

	AnMBR	eAnMBR	eRAnMBR
k_{app} (s^{-1})	0.2021	0.2009	0.2569

Supplementary Figure 17 Overall extracellular electron transfer performance of the three reactors. Cyclic voltammetry (CV) of sludge sampled from the (a) AnMBR, (b) eAnMBR, (c) eRAnMBR measured at scan rates of 60, 80, 100, 150 and 200 mV s^{-1}. (d) The apparent electron transfer rate constant (k_{app}, s^{-1}) of the sludge in the three reactors. CV curves were constructed using a three-electrode system (CHI660, Chenhua Instrument, China), in which Ag/AgCl (R0303, 6 mm× 65 mm) served as the reference electrode, a graphite plate (10 mm × 5 mm × 1 mm, 99.9%, 1.8g cm^{-3}) served as the working electrode, and a platinum sheet (10 mm × 5 mm × 1 mm, 99.99%, 21.45 g cm^{-3}) served as the opposing electrode. The sludge samples were taken from the three reactors during the stable operation period. Source data are provided as a Source Data file.

The apparent electron transfer rate constant (k_{app}, s^{-1}) was calculated based on the Laviron equation[13].

$$E_{pc}=E_c^{0'} - \left(\frac{RT}{\alpha n F}\right) \mathrm{Ln} \left(\frac{\alpha n F v_c}{RTk_{app}}\right) \quad (7)$$

$$E_{pa}=E_a^{0'} - \left[\frac{RT}{(1-\alpha) n F}\right] \mathrm{Ln} \left[\frac{(1-\alpha) n F v_a}{RTk_{app}}\right] \quad (8)$$

where α is the transfer coefficient, E_{pc} is the cathodic peak potential, E_{pa} is the anodic peak potential, v is the sweep rate, k_{app} is the apparent rate constant, T is the temperature, R is the ideal gas constant and F is the Faraday's constant (R =8.314 J·mol^{-1}·K^{-1}, F = 96483 C·mol^{-1}). The slopes of the linear portion of the (Ep - $E^{0'}$) versus ln(υ) curves are $RT/\alpha nF$ and $RT/(1 - \alpha)nF$ for the cathodic and anodic branches, respectively.

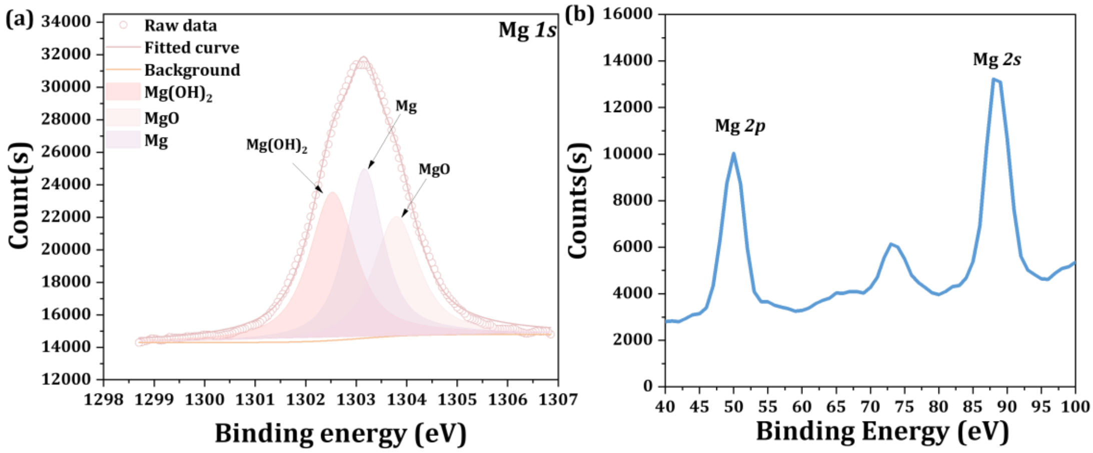

Supplementary Figure 18 XPS spectra of sludge in the eRAnMBR: (a) Mg *1s* (The data were analyzed using Xpspeak 4.1 software) and (b) Magnified view of the 40-100 eV binding energy of Figure 3e. Source data refer to data of Figure 3e.

Supplementary Table 1 Cytochrome c gene counts of the microorganisms on the membrane surface in the AnMBR and the eAnMBR. Source data are provided as a Source Data file.

KO	KEGG Name	KO Description	Gene_Count	
			AnMBR	eAnMBR
K06196	ccdA	cytochrome c-type biogenesis protein	110	124
K02197	ccmE	cytochrome c-type biogenesis protein CcmE	18	24
K02198	ccmF	cytochrome c-type biogenesis protein CcmF	51	56
K02199	ccmG, dsbE	cytochrome c biogenesis protein CcmG, thiol:disulfide interchange protein DsbE	26	31
K02200	ccmH	cytochrome c-type biogenesis protein CcmH	18	26
K08738	CYC	cytochrome c	39	48
K07399	resB, ccs1	cytochrome c biogenesis protein	37	32
Total			299	341

Supplementary Table 2 Cytochrome c gene counts of the microorganisms in sludge bulk solution of the AnMBR, eAnMBR and eRAnMBR. Source data are provided as a Source Data file.

KO	KEGG Name	KO Description	Gene_Count		
			AnMBR	eAnMBR	eRAnMBR
K02197	ccmE	cytochrome c-type biogenesis protein CcmE	23	23	27
K02198	ccmF	cytochrome c-type biogenesis protein CcmF	69	59	82
K02199	ccmG, dsbE	cytochrome c biogenesis protein CcmG, thiol:disulfide interchange protein DsbE	179	179	197
K02200	ccmH	cytochrome c-type biogenesis protein CcmH	6	7	15
K06196	ccdA	cytochrome c-type biogenesis protein	178	161	211
K07399	resB, ccs1	cytochrome c biogenesis protein	41	41	45
K08738	CYC	cytochrome c	48	41	54
K26318	fccA, fcc3	fumarate reductase (cytochrome) [EC:1.3.2.4]	15	18	22
Total			559	529	653

Supplementary References

1. Zhao, D.L., Wang, T.T., Nahan, K., Guo, X.F., Zhang, Z.P., Dong, Z.Y., Chen, S.N., Chou, D.T., Hong, D., Kumta, P.N., and Heineman, W.R., In vivo characterization of magnesium alloy biodegradation using electrochemical H2 monitoring, ICP-MS, and XPS. Acta Biomaterialia, 2017. 50: 556-565.
2. Cai, Y., Han, Z., Lin, X., Du, J., Lei, Z., Ye, Z., and Zhu, J., Mechanisms of releasing magnesium ions from a magnesium anode in an electrolysis reactor with struvite precipitation. Journal of Environmental Chemical Engineering, 2022. 10(1): 106661.
3. Song, Y.W., Han, E.H., Dong, K.H., Shan, D.Y., Yim, C.D., and You, B.S., Microstructure and protection characteristics of the naturally formed oxide films on Mg–xZn alloys. Corrosion Science, 2013. 72: 133-143.
4. Li, B., Zhao, J.H., Ge, W.Q., Li, W.P., and Yuan, H.Y., Coagulation-flocculation performance and floc properties for microplastics removal by magnesium hydroxide and PAM. Journal of Environmental Chemical Engineering, 2022. 10(2): 107263.
5. Udomkittayachai, N., Xue, W., Xiao, K., Visvanathan, C., and Tabucanon, A.S., Electroconductive moving bed membrane bioreactor (EcMB-MBR) for single-step decentralized wastewater treatment: Performance, mechanisms, and cost. Water Research, 2021. 188: 116547.
6. Ying, X.B., Huang, J.J., Shen, D.S., Feng, H.J., Jia, Y.F., and Guo, Q.Q., Fouling behaviors are different at various negative potentials in electrochemical anaerobic membrane bioreactors with conductive ceramic membranes. Science of The Total Environment, 2021. 761: 143199.
7. Kobya, M., Hiz, H., Senturk, E., Aydiner, C., and Demirbas, E., Treatment of potato chips manufacturing wastewater by electrocoagulation. Desalination, 2006. 190(1): 201-211.
8. Dominguez, L., Rodriguez, M., and Prats, D., Effect of different extraction methods on

bound EPS from MBR sludges. Part I: Influence of extraction methods over three-dimensional EEM fluorescence spectroscopy fingerprint. Desalination, 2010. 261(1-2): 19-26.

9. Khelaifia, S. and Drancourt, M., Susceptibility of archaea to antimicrobial agents: applications to clinical microbiology. Clinical Microbiology and Infection, 2012. 18(9): 841-848.
10. Vikrant, K., Roy, K., Kim, K.H., and Bhattacharya, S.S., Insights into the storage stability of ammonia in polyester aluminum bags. Environmental Research, 2019. 177: 108596.
11. Sun, Y., Lv, H., Zhou, W., and Zhang, C.M., Research on hydrogen permeability of polyamide 6 as the liner material for type IV hydrogen storage tank. International Journal of Hydrogen Energy, 2020. 45(46): 24980-24990.
12. Usman, M.R., Hydrogen storage methods: Review and current status. Renewable and Sustainable Energy Reviews, 2022. 167: 112743.
13. Laviron, E., General expression of the linear potential sweep voltammogram in the case of diffusionless electrochemical systems. Journal of Electroanalytical Chemistry and Interfacial Electrochemistry, 1979. 101(1): 19-28.
