# Supplementary material for: Recovering nutrients and unblocking the cake layer of an electrochemical anaerobic membrane bioreactor
Source: Nat Commun. 2024 Oct 22;15:9111. doi: 10.1038/s41467-024-53341-y (PMC11496669; doi:10.1038/s41467-024-53341-y)
Supplement: Supplementary file 2 — Reporting Summary [file 41467_2024_53341_MOESM2_ESM.pdf]

nature portfolio

Corresponding author(s): NCOMMS-24-29478B

Last updated by author(s): Oct 9, 2024

Reporting Summary

Nature Portfolio wishes to improve the reproducibility of the work that we publish. This form provides structure for consistency and transparency in reporting. For further information on Nature Portfolio policies, see our Editorial Policies and the Editorial Policy Checklist.

Statistics

For all statistical analyses, confirm that the following items are present in the figure legend, table legend, main text, or Methods section.

n/a	Confirmed	
☐	☒	The exact sample size (n) for each experimental group/condition, given as a discrete number and unit of measurement
☐	☒	A statement on whether measurements were taken from distinct samples or whether the same sample was measured repeatedly
☒	☐	The statistical test(s) used AND whether they are one- or two-sided<br>*Only common tests should be described solely by name; describe more complex techniques in the Methods section.*
☒	☐	A description of all covariates tested
☒	☐	A description of any assumptions or corrections, such as tests of normality and adjustment for multiple comparisons
☒	☐	A full description of the statistical parameters including central tendency (e.g. means) or other basic estimates (e.g. regression coefficient) AND variation (e.g. standard deviation) or associated estimates of uncertainty (e.g. confidence intervals)
☒	☐	For null hypothesis testing, the test statistic (e.g. F, t, r) with confidence intervals, effect sizes, degrees of freedom and P value noted<br>*Give P values as exact values whenever suitable.*
☒	☐	For Bayesian analysis, information on the choice of priors and Markov chain Monte Carlo settings
☒	☐	For hierarchical and complex designs, identification of the appropriate level for tests and full reporting of outcomes
☒	☐	Estimates of effect sizes (e.g. Cohen's d, Pearson's r), indicating how they were calculated

Our web collection on statistics for biologists contains articles on many of the points above.

Software and code

Policy information about availability of computer code

Data collection	No custom code or software was used in this study.
Data analysis	No custom code or software was used in this study.

For manuscripts utilizing custom algorithms or software that are central to the research but not yet described in published literature, software must be made available to editors and reviewers. We strongly encourage code deposition in a community repository (e.g. GitHub). See the Nature Portfolio guidelines for submitting code & software for further information.

Data

Policy information about availability of data

All manuscripts must include a data availability statement. This statement should provide the following information, where applicable:

- Accession codes, unique identifiers, or web links for publicly available datasets
- A description of any restrictions on data availability
- For clinical datasets or third party data, please ensure that the statement adheres to our policy

The data supporting the findings of this work are available within the article and its Supplementary Information files. Source data are provided with this paper. The number PRJNA1164645 gives access to raw data deposited in the NCBI Sequence Read Archive database.

Research involving human participants, their data, or biological material

Policy information about studies with human participants or human data. See also policy information about sex, gender (identity/presentation), and sexual orientation and race, ethnicity and racism.

Reporting on sex and gender	This study does not involve human participants, their data, or biological material.
Reporting on race, ethnicity, or other socially relevant groupings	This study does not involve human participants, their data, or biological material.
Population characteristics	This study does not involve human participants, their data, or biological material.
Recruitment	This study does not involve human participants, their data, or biological material.
Ethics oversight	No ethics approval was required as this study does not involve human participants or related materials

Note that full information on the approval of the study protocol must also be provided in the manuscript.

Field-specific reporting

Please select the one below that is the best fit for your research. If you are not sure, read the appropriate sections before making your selection.

☐ Life sciences ☐ Behavioural & social sciences ☒ Ecological, evolutionary & environmental sciences

For a reference copy of the document with all sections, see nature.com/documents/nr-reporting-summary-flat.pdf

Ecological, evolutionary & environmental sciences study design

All studies must disclose on these points even when the disclosure is negative.

Study description	In this study, three anaerobic membrane bioreactors were set up under different conditions: one control group and two experimental groups, operated continuously for 200 days. Basic operational parameters like pH, oxidation-reduction potential, and TMP were monitored daily, while COD, ammonium and phosphorus concentrations were measured every three days without replicates due to large data volume. Other parameters, such as gas production, EPS concentration, and struvite purity and mass, were measured at least three times during stable operation to represent the performance of the reactor. Morphological characterization using microscope (AFM, SEM, CLSM, True color confocal microscope) was conducted at 3-5 different positions for measurement. All measurements were based on samples from uniformly mixed AnMBR systems.
Research sample	In this study, three anaerobic membrane bioreactors were set up under different conditions: one control group and two experimental groups, operated continuously for 200 days. After setting the parameters initially, the reactors were operated without any further changes to these parameters. During operation, samples were taken from the uniformly mixed system to measure various indicators.
Sampling strategy	Samples were taken from the uniformly mixed AnMBR system to measure various indicators.The sample size for each index measurement was sufficient to meet the requirements of that specific index. For instance, concentrations of COD, ammonium, and phosphate were measured from both influent and effluent, typically without affecting the reactor. Samples taken from the reactor were about 10-30 ml each time, from a total reactor volume of 5L, which had no impact on reactor performance and was adequate for the analysis. Gas bags and electrode plates were replaced regularly, with measurements taken during replacement. Gas production was measured every three days during stable operation.
Data collection	The data were collected from the Analytical instruments which were used to measure various indicators of the AnMBRs as the form of electronic edition. All data were recorded by Zhang Yuhan.
Timing and spatial scale	Basic operational parameters like pH, oxidation-reduction potential, and TMP were monitored daily, while COD, ammonium and phosphorus concentrations were measured every three days without replicates due to large data volume (The data was sufficient to support the operational performance of the system). Other parameters, such as gas production, EPS concentration, and struvite purity and mass, were measured at least three times during stable operation to represent the performance of the reactor. All measurements were based on samples from uniformly mixed AnMBR systems.
Data exclusions	If external factors cause abnormalities in the system's parameters during the measurement process, the affected data will be excluded. For instance, if an issue arises with the effluent pump during operation, causing the hydraulic retention time to deviate from the set value and affecting the measurement results, the data will be discarded.
Reproducibility	During the stable operation, relevant indicators such as gas production, EPS concentration, struvite formation and so on were measured repeatedly. The experimental results were reproducible.
Randomization	In this study, the system was continuously monitored during operation, and no random groupings were involved.
Blinding	In this study, all samples were taken from the three uniformly mixed reactors, and the results were objectively obtained. Since no subjective judgment from participants was involved, blinding is not applicable.

Did the study involve field work? ☐ Yes ☒ No

Reporting for specific materials, systems and methods

We require information from authors about some types of materials, experimental systems and methods used in many studies. Here, indicate whether each material, system or method listed is relevant to your study. If you are not sure if a list item applies to your research, read the appropriate section before selecting a response.

Materials & experimental systems

n/a	Involved in the study
☒	☐ Antibodies
☒	☐ Eukaryotic cell lines
☒	☐ Palaeontology and archaeology
☒	☐ Animals and other organisms
☒	☐ Clinical data
☒	☐ Dual use research of concern
☒	☐ Plants

Methods

n/a	Involved in the study
☒	☐ ChIP-seq
☒	☐ Flow cytometry
☒	☐ MRI-based neuroimaging

Plants

Seed stocks	Plants experiment was not involved in our study.
Novel plant genotypes	Plants experiment was not involved in our study.
Authentication	Plants experiment was not involved in our study.
