# Supplementary material for: Recovering nutrients and unblocking the cake layer of an electrochemical anaerobic membrane bioreactor
Source: Nat Commun. 2024 Oct 22;15:9111. doi: 10.1038/s41467-024-53341-y (PMC11496669; doi:10.1038/s41467-024-53341-y)
Supplement: Supplementary file 3 — Source Data [file 41467_2024_53341_MOESM3_ESM.pdf]

Peer Review File

Recovering Nutrients and Unblocking the Cake Layer of an Electrochemical Anaerobic Membrane Bioreactor

Corresponding Author: Dr Chengzhi Hu

This file contains all reviewer reports in order by version, followed by all author rebuttals in order by version.

Version 0:

Reviewer comments:

Reviewer #1

(Remarks to the Author)
In the present work, Zhang et al. built an electrochemical resource-recovery AnMBR (eRAnMBR) which showed excellent performance in carbon, nitrogen, and phosphorus recovery, as well as membrane anti-fouling. Overall, the research content is meaningful and the manuscript discussed the experimental results reasonably. However, there are still some specific concerns that need to be addressed prior to be published in Nature Communications. The details were listed below:

(1) How do you determine the proportion of modules involved in methanogenesis? Please provide a complete description of the method.
(2) In Fig. 4(a), you illustrated the mechanism of membrane fouling alleviation, but the labeling here is a bit unclear. What does "Mg as another electrode" mean? Are you trying to express the membrane fouling mitigation alleviation in eRAnMBR?
(3) Mass balance of the NH4+ and PO43- should be done to have a clear idea of conversion of these chemicals to struvite.
(4) Ensure all abbreviations are defined on first mention depending on the order of your final manuscript.
(5) In line 196, what do the ccm proteins mean?
(6) In Fig.5, what is the method of Two-Dimensional correlation spectroscopy analysis? Please write in detail.
(7) The caption of Fig. 6(a) is not clear. What is the “Differences in dominant archaea” mean. The results of bacterial and archaeal community analysis are not concise enough.
(8) I think your conclusion could be further enhanced to highlight the strengths of this study.
(9) What software are the authors using for writing the REF? It’s something wrong. Example = Ye, Z.-L. This is not correct. Why are the authors writing the first name and second name? Please check all reference formats to ensure they are correct.
(10) In line 192-195, you say the “cytochrome c-type coding genes of microorganisms on the membrane 192 surface in the AnMBR and eRAnMBR”, but the results were from eAnMBR? Is it a mistake??

Reviewer #2

(Remarks to the Author)
The purpose and concept of wastewater treatment are shifting from pollution removal to resource recovery. AnMBR, as a promising approach, has attracted more and more attention because of a variety of advantages/benefits. In this study, Zhang et al. presented an electrochemical resource-recovery AnMBR (eRAnMBR) by employing a magnesium (Mg) plate and conductive membrane as dual anodes for simultaneous carbon, nitrogen, and phosphorus recovery, as well as membrane anti-fouling. Actually, the application of electrochemical method in AnMBR is not a new concept because several proof-of-concept studies have been carried out with very interesting outcomes obtained, such as “Water Research, 2022. 222: 118883, Nature Communications, 2019. 10(1): 4860.” In addition, the supply of Mg or using magnesium (Mg) plate for P and NH4+ recovery is also studied previously in electrochemical process. Overall, the novelty for this study is not high enough, and the new findings or outcomes are very limited, hindering its publication in the journal of Nature Communications. Some specific comments are as follows:
1. The main research gap is not very clear.
2. Lines 87-93, and figure 1b, COD removal for three different reactors seems similar, with only difference during days 80-120; and what’s the cause for such different? does “80 days” mean “more swiftly”? eAnMBR exhibited the same COD removal tendency toAnMBR, and why did the alkali addition from the hydrogen evolution reaction only happen in eRAnMBR but not in eAnMBR with the same posed voltage of 0.6 V?

3. figure 1C, what are the others in biogas with so high content?
4. The data regarding methane production rate and biogas composition should be provided.
5. The precipitates that formed on the cathode surface will affect the mass transfer (CO_2) and electron exchange, and how to control such technical issue if it happens?
6. The concentration of Mg^{2+} ions released into bulk solution should be provided because the size of sacrificial Mg anode will decrease and the release of Mg^{2+} will become slow.
7. Line 225-230, more explanations should be given for why the sequence of electron disturbance was re-ordered by the applied electric field.
8. Line 283, for the energy demand, how about the heating for feedstock, pump for feeding and others?
9. Line 292, "lowered the hydrophobicity of proteins in EPS" should be "increased", contradictory to the lines 160-164: "Due to the twisted and pleated structure of the β-sheets, the inner hydrophobic groups of amino acids were more readily exposed, thus expressing the hydrophobic properties of the sludge flocs. By altering the secondary structure of the EPS proteins, Mg^{2+} strengthened sludge particle hydrophobicity, thereby reducing sludge viscosity and improving sludge floc aggregation".
10. The findings of this study seems predictable without in-depth insights into either reactor configuration or mechanisms.

Reviewer #3

(Remarks to the Author)
This paper investigates a novel resource-recovery anaerobic membrane bioreactor and delves into its mechanism. The work is intriguing, but I believe there are still many issues that need to be addressed to meet publication standards. My comments are listed below:

Comment 1: Fig.4b: What is the control membrane and electro-membrane?
Comment 2: Fig.2c: I noticed the same image appearing in both Fig.2 and Fig.S3, but they appear inconsistent. Please check.
Comment 3: Section "Microbial anodic oxidation and its effect on EPS": "These results confirmed that microbial anodic oxidation was strengthened, leading to the decomposition of complex organics in the cake layer of the membrane-anode" However, the author only focused on proteins in the explanation of CLSM images. It should be noted that complex organics are not limited to proteins alone.
Comment 4:Section"Antifouling performance and optimization of cake layer structure": The analysis of protein secondary structure quite interesting, but the correlation with membrane fouling is inadequately described. It is necessary for the author to improve their explanation of this aspect of the mechanism.
Comment 5: Section "Carbon, nitrogen, and phosphorus recovery in eRAnMBR":"And from the Energy-dispersive X-ray spectroscopy (EDS) spectrogram as well as the atom proportion, we can draw a conclusion that except struvite there may be a small amount of other Mg phosphate phase generating on the cathode surface." What does this sentence mean here? The author should clarify the research focus of this paragraph.
Comment 6: Write the future research perspectives in conclusions section. The present information is VERY short and more inclined towards lab studies. The entire world is now shifting from lab to pilot scale works and its important that the authors look beyond the lab for future applications.

Moderate comments:
Comment 1: Figures and tables should be referenced within the text to guide the reader through the data presentation.
Comment 2: More information should be included in Introduction to underscore the significance of this research.
Comment 3: There appears to be a lack of a coherent logical connection between the various sections of the paper. It is necessary for the author to improve it.
Comment 4: The section of method in the manuscript is quite lengthy, but there are still some details of the methods that are not clearly written. It is necessary for the author to supplement or move to SI.

Version 1:

Reviewer comments:

Reviewer #1

(Remarks to the Author)
I have confirmed that the manuscript has been modified and improved based on my previous comments.

Reviewer #2

(Remarks to the Author)
Authors have reivsed the manuscript and it can be acceptable.

Reviewer #3

(Remarks to the Author)

I have reviewed the revised manuscript and the response, and think the comments have been well addressed by the authors. I suggest the manuscript can be accepted in the present form.

Open Access This Peer Review File is licensed under a Creative Commons Attribution 4.0 International License, which permits use, sharing, adaptation, distribution and reproduction in any medium or format, as long as you give appropriate credit to the original author(s) and the source, provide a link to the Creative Commons license, and indicate if changes were made.
In cases where reviewers are anonymous, credit should be given to 'Anonymous Referee' and the source.
The images or other third party material in this Peer Review File are included in the article's Creative Commons license, unless indicated otherwise in a credit line to the material. If material is not included in the article's Creative Commons license and your intended use is not permitted by statutory regulation or exceeds the permitted use, you will need to obtain permission directly from the copyright holder.
To view a copy of this license, visit https://creativecommons.org/licenses/by/4.0/

Reviewer #1 (Remarks to the Author):

Reviewer: *In the present work, Zhang et al. built an electrochemical resource-recovery AnMBR (eRAnMBR) which showed excellent performance in carbon, nitrogen, and phosphorus recovery, as well as membrane anti-fouling. Overall, the research content is meaningful and the manuscript discussed the experimental results reasonably. However, there are still some specific concerns that need to be addressed prior to be published in Nature Communications. The details were listed below:*

Our Response: We would like to express our sincere thanks to the reviewer for the constructive comments and suggestions to improve the quality of this manuscript.

Reviewer (Comment 1): *How do you determine the proportion of modules involved in methanogenesis? Please provide a complete description of the method.*

Our Response: Thanks for your kind suggestion. We agree that it is essential to provide a detailed description of the method used to determine the proportion of modules involved in methanogenesis. This has been included in the revised manuscript to enhance the clarity and facilitate readers understanding.

> **-Line 563** BLASTP v2.2.28+ (http://blast.ncbi.nlm.nih.gov/Blast.cgi) was used to compare non-redundant gene sequences with the Kyoto Encyclopedia of Genes and Genomes (KEGG) database, with the expected value of BLAST comparison parameters set to 1e^{-5}. Based on the comparative results, functional annotation was conducted using KOBAS 2.0 [4] (KEGG Orthology Based Annotation System). The abundance of genes corresponding to KO, Pathway, EC, and Module was summed to calculate the abundance of this functional category.

Reviewer (Comment 2): *In Fig. 4(a), you illustrated the mechanism of membrane fouling alleviation, but the labeling here is a bit unclear. What does "Mg as another electrode" mean? Are you trying to express the membrane fouling mitigation alleviation in eRAnMBR?*

Our Response: Thanks for your kind suggestion. We realized that our initial explanation and labeling were not sufficiently clear, which may have led to some confusion. In response, the labeling and the figure caption were revised to more accurately and clearly reflect the intended mechanism, ensuring that the diagram more effectively communicated our findings. The intention of Fig.4a was to illustrate the mechanism of membrane fouling alleviation when the membrane served as the anode, as well as to demonstrate the effect on fouling mitigation when Mg was used as another anode in a dual-anode switching system. We appreciate your insight and have made the necessary adjustments accordingly.

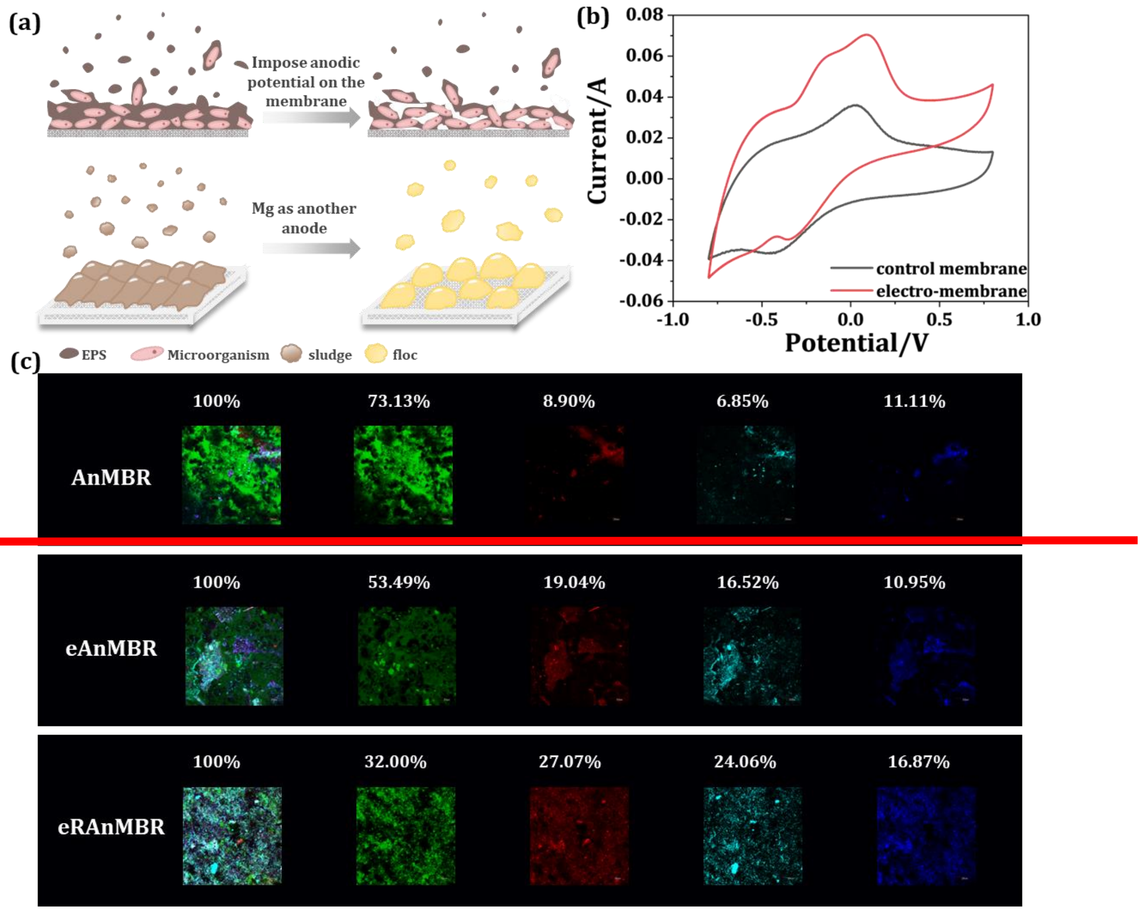

Fig.4 (a) Effects of Mg on sludge and anode potential on membrane fouling alleviation. (b) Cyclic voltametric analyses of control membrane and electro-membrane in the 0.8 to −0.8 V range at scan rates of 2 mV/s using a three-electrode system, with Ag/AgCl as the reference electrode, conductive membrane as the working electrode, and graphite cathode as the opposing electrode. Control membrane was taken from AnMBR, and electro-membrane was taken from eAnMBR (c) CLSM images of membrane surface of three reactors (total cells are in red, proteins are in green, α-polysaccharides are in purple, and β-polysaccharides are in blue).

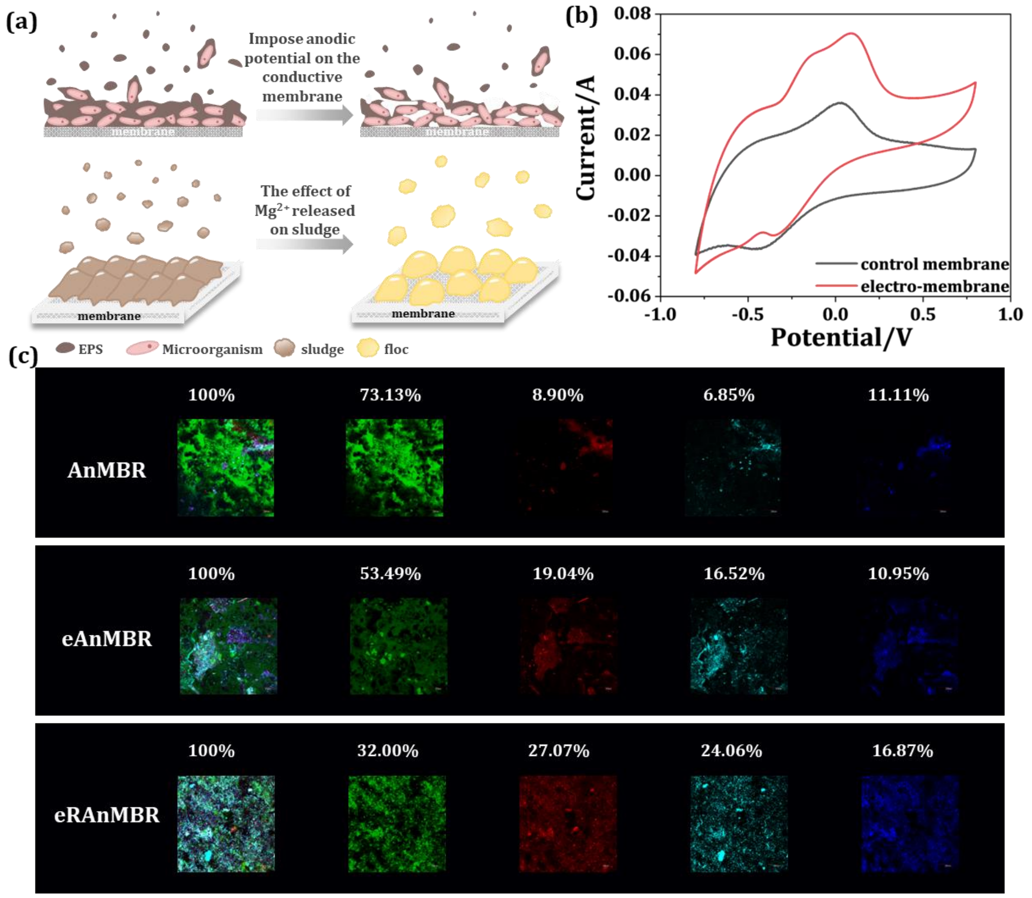

Fig.4 (a) ~~Effects of Mg on sludge and anode potential on membrane fouling alleviation.~~ (a) Membrane fouling mitigation of the eRAnMBR: effects of anode potential on the cake layer and Mg^{2+} released on sludge. (b) Cyclic voltametric analyses of control membrane and electro-membrane in the 0.8 to −0.8 V range at scan rates of 2 mV/s using a three-electrode system, with Ag/AgCl as the reference electrode, conductive membrane as the working electrode, and graphite cathode as the opposing electrode. Control membrane was taken from the AnMBR, and electro-membrane was taken from the eAnMBR. (c) CLSM images of membrane surface of three reactors (total cells are in red, proteins are in green, α-polysaccharides are in purple, and β-polysaccharides are in blue).

Reviewer (Comment 3): *Mass balance of the NH_4^+ and PO_4^{3-} should be done to have a clear idea of conversion of these chemicals to struvite.*

Our Response: Thanks for your kind suggestion. We agree that performing a mass balance for NH_4^+ and PO_4^{3-} was crucial for a clearer understanding of these chemicals' conversion to struvite. In response, the mass balance for these compounds was calculated, and the results have been added into the Supplementary Information (SI) as a figure (Fig.S11). Additionally, a more detailed analysis was provided in the corresponding section of the main manuscript.

-line 186 Calculating through the method of determination of the struvite on the cathode surface, the purity of struvite was approximately 60.64% and the mass was 0.08 g/d, which meant approximately 9.04% struvite generated in the reactor was deposited on the graphite cathode surface (Fig.S11).

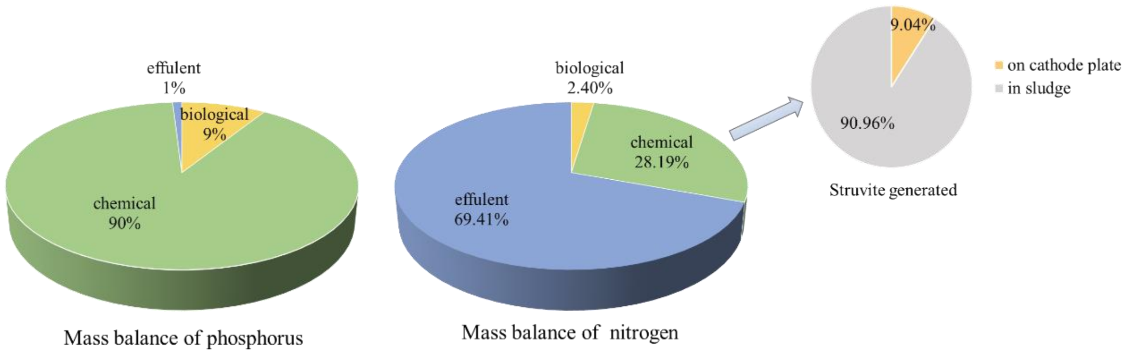

Fig.S11 Mass balance of the phosphorus and nitrogen in the eRAnMBR.

<u>Reviewer (Comment 4)</u>: *Ensure all abbreviations are defined on first mention depending on the order of your final manuscript.*

Our Response: Thanks for your kind recommendation. We have checked all abbreviations to ensure they are defined the first time on the order of my manuscript.

<u>Reviewer (Comment 5)</u>: *In line 196, what do the ccm proteins mean?*

Our Response: We thank the reviewers for their insightful observation. The gene abundance comparison was presented in Table.S1, and relevant explanations have been added in the manuscript to better clarify this finding. In the comparison of cytochrome c gene abundance in microbial on the membrane, depending on whether an anode potential was applied, the ccm protein showed significant differences. Its function was involved in the post-translational modifications essential for Mtr pathway protein production[1].

-line 288 And the cytochrome c-type coding genes associated with bioelectrochemical oxidation[41] of microorganisms on the membrane surface also increased by 14%, from 299 on the control membrane of AnMBR to 341 on the electro-membrane of the ~~eRAnMBR~~eAnMBR (Table S1), especially the ccm proteins involved in post-translational modifications essential for Mtr pathway protein production[42].

Table.S1 Cytochrome c gene counts of the microorganisms on the membrane surface in the AnMBR and the eAnMBR

KO	KEGG Name	KO Description	Gene_Count	
			AnMBR	eAnMBR
K06196	ccdA	cytochrome c-type biogenesis protein	110	124
K02197	ccmE	cytochrome c-type biogenesis protein CcmE	18	24
K02198	ccmF	cytochrome c-type biogenesis protein CcmF	51	56
K02199	ccmG, dsbE	cytochrome c biogenesis protein CcmG, thiol:disulfide interchange protein DsbE	26	31
K02200	ccmH	cytochrome c-type biogenesis protein CcmH	18	26
K08738	CYC	cytochrome c	39	48
K07399	resB, ccs1	cytochrome c biogenesis protein	37	32
Total			299	341

Reviewer (Comment 6): *In Fig.5, what is the method of Two-Dimensional correlation spectroscopy analysis? Please write in detail.*

Our Response: Thank you very much for your reminder. The method of Two-Dimensional correlation spectroscopy analysis has been added to the Method section in the manuscript.

-line 495 All extracted EPS samples were measured using a three-dimensional excitation emission matrix (EEM) (F-4600, HITACHI, Japan) and Fourier transform infrared spectroscopy (FTIR) (Nicolet iN10MX, Thermo Fisher Scientific, USA). The software 2D Shige ver. 1.3 (Shigeaki Morita, Kwansei-Gakuin University, Japan) was used to analyze the infrared spectra data of LB-EPS and SMP, and then obtained the 2D-COS spectra to gain insights into the molecular compositions and formation mechanisms of organic substances.

Reviewer (Comment 7): *The caption of Fig. 6(a) is not clear. What is the “Differences in dominant archaea” mean. The results of bacterial and archaeal community analysis are not concise enough.*

Our Response: Thank you for your insightful feedback. We realized that this was not clearly conveyed in the original figure, so the revised figure title was added to make the image's message clearer. The intention of Fig. 6a was to show that in the comparison of archaeal abundance among the three different reactors, it was found that *Methanobacterium* was the dominant archaea in both the AnMBR and the eAnMBR. However, in the eRAnMBR, the abundance of *Methanosarcina* significantly increased, reaching 43.9%. Consequently, the differences between these two archaeal communities in terms of EPS secretion (which plays a role in membrane fouling alleviation) and interspecies electron transfer were compared[2-4].

Regarding your concern about the results of bacterial and archaeal community analysis are not concise enough, we acknowledged that our analysis in this section did not effectively highlight the key points. Therefore, the title of this part and the

entire paragraph was revised to emphasize how the changes in the microbial community promote the increase in methane (CH_4) purity and production in the eRAnMBR. Firstly, we discussed the changes in the archaeal community, and followed by the analysis of the electron transfer pathways, making the overall analytical framework clearer.

-line 341 **~~Evolution of microbial community structure under electrical stimulation~~**

~~The relative abundance of microbial genera in the AnMBRs is shown in Fig. 6. Among bacteria, two genera increased markedly after bias application. Notably, DMER64 from 2.7% to 8.1%, a fermentative genus capable of establishing direct interspecific electron transfer (DIET) with methanogens due to its electroactivity and improve methane rate. The other is norank_f__Synergistaceae from 6.3% to 12.8%, which belonged to the phylum Synergistetes that can coexist with methanobacteria, accelerating hydrolysis and generating small molecules that can be utilized by methanobacteria to enhance methanogenesis[44]. Methanobacterium was the dominant archaea genus in the control AnMBR, with a relative abundance of 92.9%. In contrast, following the application of an electric field, the relative abundance of Methanosarcina increased to 5.6% in the eAnMBR and 43.9% in the eRAnMBR. Methanosaeta and Methanosarcina, the only methanogen genera with membrane-bound cytochromes[45], may facilitate extracellular electron exchange. Of note, Methanosarcina growth can be induced by an electric field due to its capability for direct interspecific electron transfer[46], which is favorable for methane production[47]. Moreover, Methanosarcina produces less EPS than Methanobacterium[48] which can help alleviating membrane fouling, and it produce methane through all the three methanogenesis pathways (acetoclastic, hydrogenotrophic, and methylotrophic methanogenesis)[23] providing a more stable and perfect metabolic system[49]. As shown in Fig. S9, all relative abundances of modules involved in methanogenesis increased in the eRAnMBR, thus promoting methane production.~~

Microbial community succession and its enhancement of CH_4 production

The analysis of archaeal community also explained the mechanism behind enhanced CH_4 production of the eRAnMBR. From Fig. 6b we can see that Methanobacterium was the dominant archaea genus in the control AnMBR, with a relative abundance of 92.9%. In contrast, following the application of an electric field, the relative abundance of Methanosarcina increased to 5.6% in the eAnMBR and 43.9% in the eRAnMBR. The abundance of Methanosarcina increased in the eAnMBR was favorable for CH_4 production[50], due to its capability to produce CH_4 through all the three methanogenesis pathways (acetoclastic, hydrogenotrophic, and methylotrophic methanogenesis)[23, 52]. As shown in Fig.S16, all relative abundances of modules involved in methanogenesis increased in the eRAnMBR. Besides, Methanosaeta and Methanosarcina, the only methanogen genera with membrane-bound cytochromes[48], may facilitate extracellular electron exchange and DIET process[49]. The abundance of DMER64 genus possessing DIET ability and the cytochrome c-type coding genes related to DIET also increased (Fig.6c and Table.S2). And the apparent electron transfer rate constant (kapp) of the eRAnMBR was higher than the other two reactors (Fig.S17), indicating that the overall extracellular electron transfer performance was strengthened[53]. DIET, considered to be a more efficient electron transfer pathway[54] without relying on the diffusion of electron carriers such as H_2, was conducive to the increase of CH_4 yield in the eRAnMBR. In addition, Methanosarcina produced less EPS than Methanobacterium[51] which can help alleviating membrane fouling.

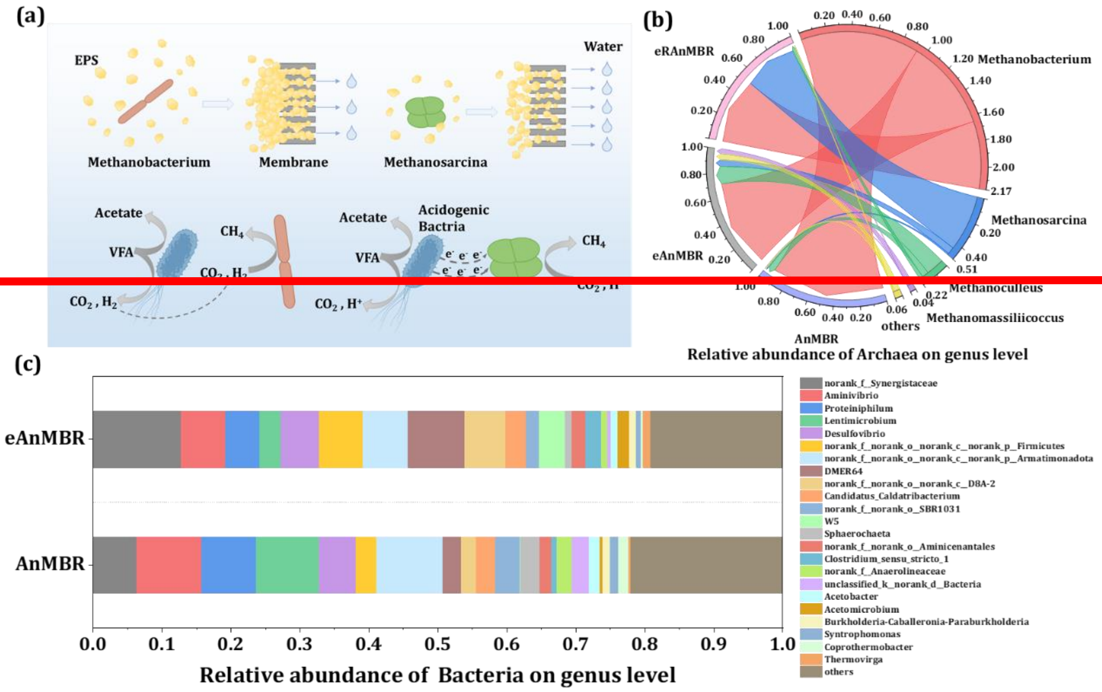

Fig.6 (a) Differences in dominant archaea related to EPS secretion and electron transport. (b) Relative abundance of archaea at genus level in the AnMBR, the eAnMBR, and the eRAnMBR. (c) Relative abundance of bacteria at genus level in the AnMBR and the eAnMBR. The anodes of the eAnMBR comprised a conductive membrane and a graphite plate, while the anodes of the eRAnMBR included a conductive membrane and a Mg plate. Applied voltage of both the eRAnMBR and the eAnMBR was 0.6 V. (Note: the word electro AnMBR means we mention both the eRAnMBR and the eAnMBR).

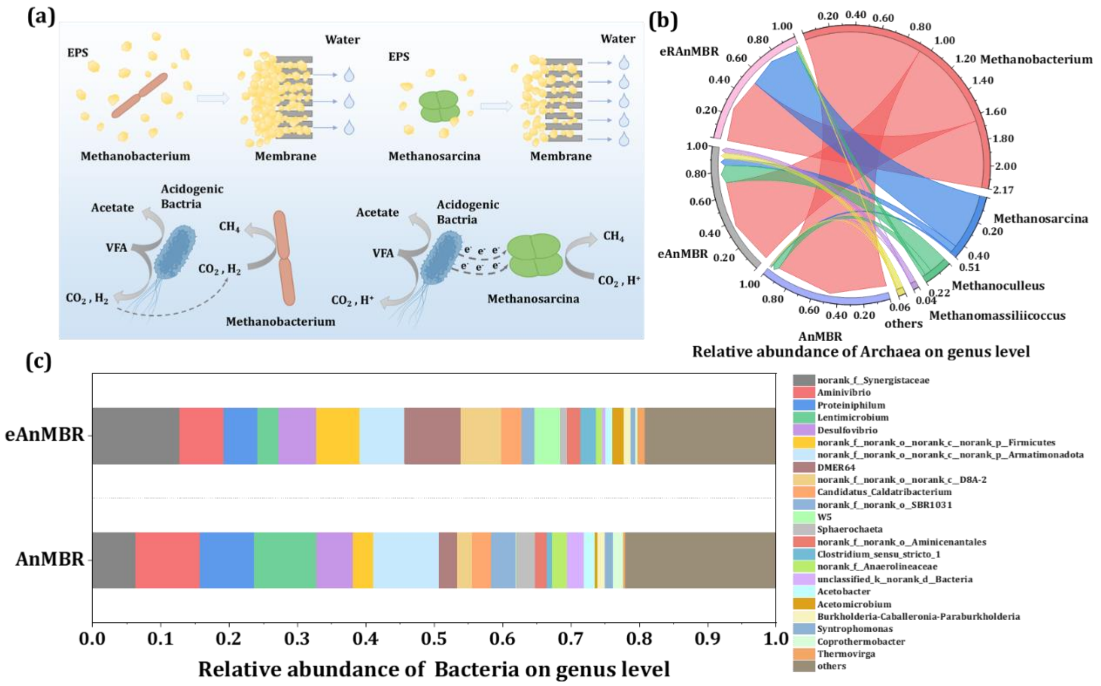

Fig.6 (a) Differences in dominant archaea related to EPS secretion and electron transport.

Differences of *Methanobacterium* and *Methanosarcina* on EPS secretion and electron transport. *Methanobacterium* was the only dominant archaea in the AnMBR, the eAnMBR, while *Methanosarcina* was also the dominant archaea in the eRAnMBR. (b) Relative abundance of archaea at genus level in the AnMBR, eAnMBR, and eRAnMBR. (c) Relative abundance of bacteria at genus level in the AnMBR and eAnMBR. The anodes of the eAnMBR comprised a conductive membrane and a graphite plate, while the anodes of the eRAnMBR included a conductive membrane and a Mg plate. Applied voltage of both the eRAnMBR and eAnMBR was 0.6 V. (Note: the word electro-AnMBR means we mention both the eRAnMBR and eAnMBR).

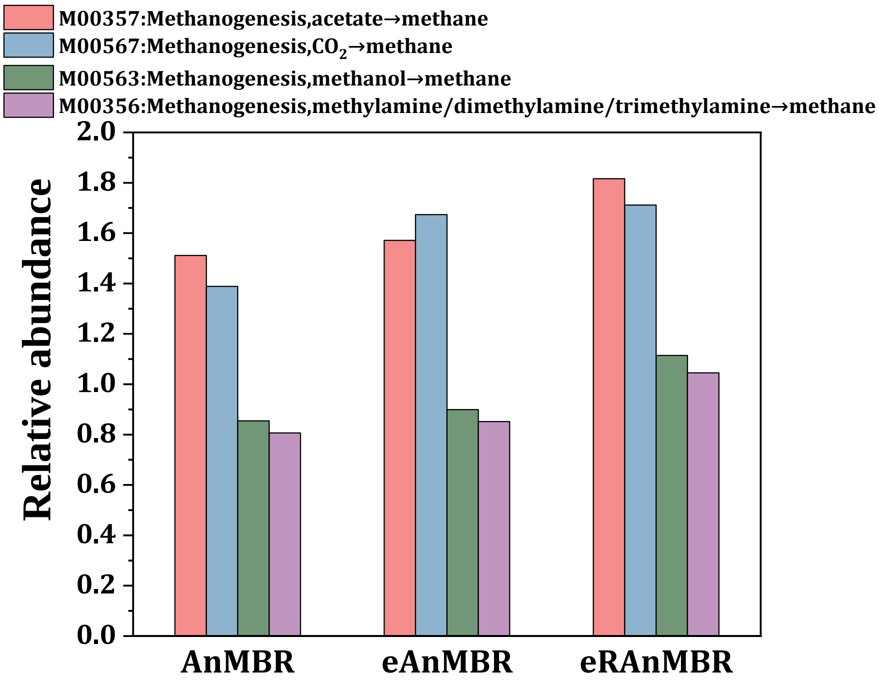

Fig.S16 The relative abundance of modules involved in methanogenesis.

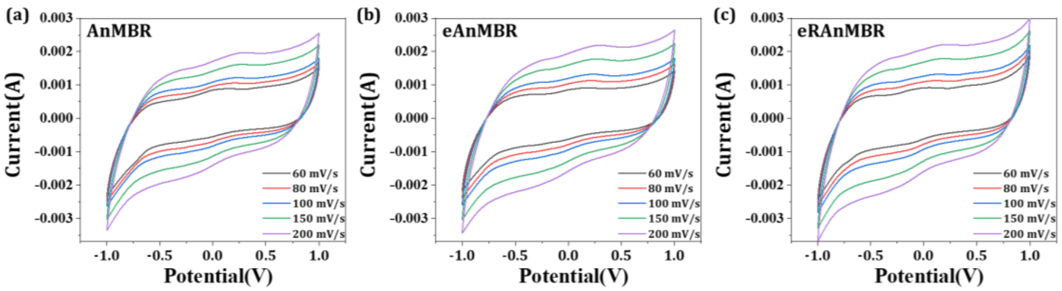

(d)

	AnMBR	eAnMBR	eRAnMBR
k_{app} (s^{-1})	0.2021	0.2009	0.2569

Fig.S17 Cyclic voltammetry (CV) of sludge sampled from the (a) AnMBR, (b) eAnMBR, (c) eRAnMBR measured at scan rates of 60, 80, 100, 150 and 200 mV/s. (d) The apparent electron transfer rate constant (kapp, s^{-1}) of the sludge in the three reactors. CV curves were constructed using a three-electrode system (CHI660, Chenhua Instrument, China), in which Ag/AgCl served as the reference electrode, a graphite plate served as the working electrode, and a platinum sheet served as the opposing electrode. The sludge samples were taken from the three reactors during

the stable operation period. The apparent electron transfer rate constant (k_{app}, s^{-1}) was calculated based on the Laviron equation[9].

Table.S2 Cytochrome c gene counts of the microorganisms in sludge bulk solution of the AnMBR, eAnMBR and eRAnMBR.

KO	KEGG Name	KO Description	Gene_Count		
			AnMBR	eAnMBR	eRAnMBR
K02197	ccmE	cytochrome c-type biogenesis protein CcmE	23	23	27
K02198	ccmF	cytochrome c-type biogenesis protein CcmF	69	59	82
K02199	ccmG, dsbE	cytochrome c biogenesis protein CcmG, thiol:disulfide interchange protein DsbE	179	179	197
K02200	ccmH	cytochrome c-type biogenesis protein CcmH	6	7	15
K06196	ccdA	cytochrome c-type biogenesis protein	178	161	211
K07399	resB, ccs1	cytochrome c biogenesis protein	41	41	45
K08738	CYC	cytochrome c	48	41	54
K26318	fccA, fcc3	fumarate reductase (cytochrome) [EC:1.3.2.4]	15	18	22
Total			559	529	653

Reviewer (Comment 8): *I think your conclusion could be further enhanced to highlight the strengths of this study.*

Our Response: Thanks for your kind suggestion. The original conclusion has been rewritten to provide a more in-depth analysis, as the initial version was more of a list of results and lacked depth.

The focus of this study was on developing a novel anaerobic membrane bioreactor capable of simultaneously recovering carbon, nitrogen, and phosphorus, and thoroughly investigating how changes in sludge physicochemical properties contributed to mitigating membrane fouling. The deposition of struvite (formed from NH_4^+ and PO_4^{3-}) on the electrode plates facilitated the separation from sludge. The CH_4 production rate reached 93.97%, making it a viable energy fuel[5-8]. The presence of Mg electrodes intensified hydrogen evolution at the cathode, with the hydrogen produced serving as a substrate for methanogenesis. The generated alkalinity improved the absorption of carbon dioxide (CO_2) from biogas, reducing its proportion. Furthermore, Mg anode altered the microbial community structure, promoted DIET, and enhanced multiple CH_4 metabolism pathways, thereby increasing CH_4 production.

The membrane fouling mitigation efficiency in the eRAnMBR reached 95%, with changes in sludge properties playing a more significant role in fouling reduction than electrokinetic forces alone. The addition of Mg anodes significantly reduced the interaction forces between sludge particles and the membrane, enhanced sludge aggregation, thus slowing down the formation of a gel layer and resulting in a loose, porous cake layer.

An in-depth analysis and summary of all the mechanisms have been conducted. The corresponding revisions are provided as follows.

-line 395 Discussion

To strengthen energy and resource recovery as well as membrane anti-fouling, a novel eRAnMBR featuring dual anodes—an Mg anode and a membrane-anode—was developed. Notably, the eRAnMBR system outperformed traditional AnMBRs, removing 30.59% of NH_4^+ and 99% of PO_4^{3-}, while producing struvite with 60.64% purity and depositing 0.08 g/d on the cathode surface for easy separation from the sludge. The eRAnMBR system also upgraded biogas to biomethane, significantly increasing its calorific value and facilitating its recovery as natural gas. Considering the conversion efficiency of methane to electricity, the average energy production capacity of the eRAnMBR was 883.35 Wh/m^3, far exceeding the energy demand (0.015 Wh/m^3), demonstrating the feasibility of using self-sufficient power for sewage treatment. The operating cost analysis indicated that the average energy production capacity of the eRAnMBR, exceeded the additional energy and electrode material consumption, demonstrating the economic viability of this configuration (Text.S3). The enhancement of DIET and all methanogenesis pathways within the system, along with the H_2 evolution and alkalinity production after the application of the Mg anode, all contributed to the increase in CH_4 purity and yield. Regarding antifouling performance, the eRAnMBR achieved a 95% reduction compared to traditional AnMBRs, primarily attributed to two reasons. First, the release of Mg^{2+} altered the physicochemical properties of the sludge, generating large flocs and reducing the adhesion force between the membrane surface and sludge, thus slowing the formation of an irreversible cake layer. Second, the strength of microbial anodic oxidation decomposed the proteins in the cake layer after applying anodic potential to the conductive membrane. The eRAnMBR reduced the secretion and lowered the hydrophobicity of proteins in EPS, and made them more likely to engage in redox reactions that accelerate the electron transport rate. Furthermore, the increased relative abundance of *DMER64* and *Methanosarcina* indicated enhanced direct interspecific electron transfer following the application of the electric field, and all methanogenesis-related modules were strengthened in the eRAnMBR, thus promoting methane production efficiency. The eRAnMBR introduces a novel approach to anaerobic digestion for more sustainable wastewater treatment. Future research should focus on improving the proportion of struvite deposited on the cathode surface for easier nutrient recovery, and on optimizing the use of collected methane to offset the greenhouse gas footprint, thereby maximizing the application potential of integrated AnMBR systems. In-depth research on the physicochemical properties of the sludge explained the 95% reduction in membrane fouling of the eRAnMBR. The released Mg^{2+} increased floc size, decreased the adhesion force between the sludge particles and the membrane surface, and reduced the proportion of hydrogen bonds in the secondary structure of EPS proteins, thereby weakening the formation of the gel layer and making the cake layer structure loose and porous. The application of the electric field also enhanced the microbial anodic oxidation, and made proteins more likely to participate in redox reactions.

In this study, it was found that alleviating membrane fouling by regulating the cake layer through altering the physicochemical properties of the sludge was far more effective than the singular electrodynamic effect. This provided a new approach for extending membrane lifespan in future applications. The new configuration, which switched between the membrane and Mg anodes, reduced Mg material input while effectively recovering three types of resources, enabling the application of AnMBRs in a more sustainable wastewater treatment. As for industrial applications, more economical anode materials, electrode configurations that highly facilitate mass transfer and struvite precipitation, and more efficient utilization of the generated CH_4 to reduce costs are engineering considerations for the practical application of this reactor.

Reviewer (Comment 9): *What software are the authors using for writing the REF? It's something wrong. Example = Ye, Z.-L. This is not correct. Why are the authors writing the first name and second name? Please check all reference formats to ensure they are correct.*

Our Response: Thank you very much for your reminder. We used EndNote to write the reference, but it is possible that some names were garbled in the process. We have carefully checked the references one by one to ensure that this issue has been resolved.

Reviewer (Comment 10): *In line 192-195, you say the "cytochrome c-type coding genes of microorganisms on the membrane 192 surface in the AnMBR and eRAnMBR", but the results were from eAnMBR? Is it a mistake??*

Our Response: Thank you very much for your reminder. We apologized for the typographical error here. We have revised in the manuscript and carefully reviewed the article to ensure that such errors would not occur again. This content aimed to compare the control membrane in the control AnMBR with the electro-membrane in the eAnMBR where an anodic potential was applied.

> **-line 288** And the cytochrome c-type coding genes associated with bioelectrochemical oxidation[41] of microorganisms on the membrane surface also increased by 14%, from 299 on the control membrane of AnMBR to 341 on the electro-membrane of the ~~eRAnMBR~~eAnMBR (Table S1).

Reviewer #2 (Remarks to the Author):

Reviewer： *The purpose and concept of wastewater treatment are shifting from pollution removal to resource recovery. AnMBR, as a promising approach, has attracted more and more attention because of a variety of advantages/benefits. In this study, Zhang et al. presented an electrochemical resource-recovery AnMBR (eRAnMBR) by employing a magnesium (Mg) plate and conductive membrane as dual anodes for simultaneous carbon, nitrogen, and phosphorus recovery, as well as membrane anti-fouling. Actually, the application of electrochemical method in AnMBR is not a new concept because several proof-of-concept studies have been carried out with very interesting outcomes obtained, such as "Water Research, 2022. 222: 118883, Nature Communications, 2019. 10(1): 4860." In addition, the supply of Mg or using magnesium (Mg) plate for P and NH4+ recovery is also studied previously in electrochemical process. Overall, the novelty for this study is not high enough, and the new findings or outcomes are very limited, hindering its publication in the journal of Nature Communications. Some specific comments are as follows:*

Our Response: We sincerely appreciate the valuable comments from the reviewers, which have helped us recognize our shortcomings. Although the mechanisms of membrane fouling mitigation and nitrogen and phosphorus recovery using magnesium (Mg) have been studied, incorporating Mg anodes into the sludge system has rarely been explored. In our study, the eRAnMBR realized simultaneous recovery of carbon, nitrogen, and phosphorus in the biological system through Mg while effectively mitigating membrane fouling. Moreover, an in-depth investigation into the microbial system and the physicochemical properties of the sludge were conducted during this process. With the reviewers' assistance, we have highlighted our innovations, making the logic clearer and the mechanistic explanations more comprehensive. Our specific responses to the reviewer's comments are given below.

Reviewer (Comment 1): *The main research gap is not very clear.*

Our Response: Thanks for pointing out this issue. We have modified the introduction to highlight the research gap and conducted an in-depth analysis of the mechanisms involved in the study.

As one of the most effective low-carbon wastewater treatment technologies, AnMBRs offer excellent application prospects in sustainable water treatment. This article focused on a novel eRAnMBR featuring dual anodes-an Mg anode and a membrane-anode, significantly alleviating membrane fouling to 95%, enhancing CH_4 purity to 93.97% and recovering nitrogen and phosphorus, significantly addressed the issues faced by traditional AnMBRs. The main research gap was as follows:

(1) Conventional AnMBRs lack the ability to remove NH_4^+ and PO_4^{3-}, and may even release them during this process[9, 10]. These substances are actually valuable

resources. Although the mechanisms of NH_4^+ and PO_4^{3-} recovery using Mg have been studied, these studies have primarily been conducted in abiotic systems, and the incorporation of Mg anodes into bioreactors has rarely been explored. In this study, the removal rate of PO_4^{3-} realized 99%. And the hydrogen evolution increased the localized pH at the cathode surface, creating favorable conditions for struvite precipitation, which facilitated the separation of struvite from the sludge for recovery

(2) Biogas produced by anaerobic processes plays a key role in the emerging market for renewable energy, but requires complicated operating systems to increase the calorific value and reduce unwanted components, e.g. CO_2[11]. Unlike previous studies on CH_4 production enhancement, this reactor didn't rely on a large input of H_2 or enhanced cathodic CO_2 reduction to CH_4[4, 12]. Instead, it achieved improvements in methane purity and yield by enhancing the Direct Interspecies Electron Transfer process (DIET) and promoting CO_2 mineralization which realized effective and economical in-situ biomethane upgrading.

(3) Conventional AnMBRs suffer from severe membrane fouling issues[13]. Unlike aerobic membrane bioreactors, research on the mechanisms of the regulation of the cake layer in AnMBRs is relatively scarce. In this study, an in-depth study on the effects of changes in the physicochemical properties of sludge on the structure of the gel layer and cake layer. The Mg^{2+} released increased floc size, decreased the adhesion force between the sludge particles and the membrane surface, and reduced the proportion of hydrogen bonds in the secondary structure of EPS proteins, thereby weakening the formation of the gel layer and making the cake layer structure loose and porous.

The revisions of the introduction and the related mechanistic research are as follows.

-line 37 Introduction

In the context of sustainable development, sewage treatment facilities are not only required to recover renewable resources but also achieve carbon emission reduction. Anaerobic membrane bioreactors (AnMBRs), which integrate anaerobic processes with membrane separation, are among the most effective low-carbon wastewater treatment technologies, offering excellent application prospects in sustainable water treatment. However, challenges such as membrane fouling[1,2] and the inability to recover nitrogen and phosphorus[3,4] significantly restrict the large-scale application of AnMBRs in wastewater treatment[5]. Addressing these issues could greatly enhance their efficiency and application scope[6]. Besides, biogas produced by anaerobic digestion processes plays a key role in the emerging market for renewable energy[7]. However, in addition to 60–70% methane (CH_4), raw biogas contains 30–40% carbon dioxide (CO_2) which decreases its specific calorific value[8]. Therefore, the ratio of CH_4 to CO_2 needs to be increased for biogas to work as an effective fuel source[9]. Furthermore, optimizing biogas production and upgrade to harness this renewable energy source more effectively is a critical future direction for anerobic process, thus supporting the goal of sustainable wastewater development and treatment.

Various methods for controlling membrane fouling have been reported, including biogas sparging, backwashing, acid cleaning, and bacterial quorum quenching[10]. Numerous methods have been employed to resist the membrane fouling[10.] Recently, a novel approach that incorporates an electric field in AnMBRs has garnered considerable attention as a chemical-free and efficient way to alleviate *in-situ* membrane pollution[11,12], avoiding the use of chemical agents and preventing secondary pollution. The electrostatic repulsion at the cathode effectively inhibited the deposition of negatively charged extracellular polymeric substances (EPS) and soluble microbial products (SMP), which are the main substances contributing to membrane fouling. Most studies have focused on the electrophoresis, electrostatic forces, and other electrodynamic effects of the electric field on the membrane fouling[11-13], while research on the mechanisms of the regulation of the cake layer in AnMBRs is relatively scarce. However, altering the physicochemical properties of the sludge can directly influence the substances that form membrane fouling, thus modifying the structure of the cake layer or slowing its formation.

As for nitrogen and phosphorus removal, struvite precipitation is considered an excellent method, with recovered struvite further serving as a slow-release fertilizer[14]. Among the various struvite precipitation methods, electrochemical processes are considered promising due to their high efficiency in nitrogen and phosphorus removal[15,16], coupled with the advantages of simple operation, controllable reactions, and reduced sludge production[17,18]. The electrochemical method mitigates membrane fouling via electrocoagulation, electrophoresis, and electrochemical oxidation[11-13]. Struvite generation utilizes sacrificial magnesium (Mg) anodes, where anode-released Mg ions (Mg^{2+}) react with ammonium (NH_4^+) and phosphate (PO_4^{3-}) ions in bulk solution to form struvite[19]. Typically, struvite precipitation is used as a post-treatment process following biological treatment[15,16], and its application within the bioreactor is rare which faces the problem of the separation of struvite and sludge. If Mg anode was introduced into the AnMBR system, it could exert dual effects of the nitrogen and phosphorus removal and electrochemical regulation on membrane fouling. Additionally, the Mg^{2+} ions and hydroxide ions (OH^-) produced from the hydrogen evolution reaction at the cathode can absorb CO_2 produced by anaerobic digestion, synergizing CO_2 mineralization and biogas upgrading[20]. Understanding how the Mg anode impacts the biological system during this process is a key area that warrants further investigation. Therefore, the electrochemical process enables simultaneous membrane antifouling and carbon, nitrogen, and phosphorus recovery. To date, however, few studies have explored these challenges and improved biogas performance within a single reactor.

-line 233 Third, Mg^{2+} changed the secondary structure of protein weakened the formation of gel layer. As a typical negatively charged and sticky substance[4], the contribution of proteins to membrane fouling in the AnMBRs was more than that of polysaccharides in EPS. In comparison to the AnMBR and the eAnMBR, EPS proteins of the eRAnMBR exhibited fewer α-helices and more β-sheets, contributing to the high aggregation capability of the sludge[32,33] (Fig.S13). A lower α-helix/(β-sheet + random coil) ratio suggested that the proteins in the eRAnMBR possessed less hydrogen bonds[35-37] which played a key role in the gel layer[38,39,] thus weakening the formation of a compact foulants layer .

-line 327 The intricate structure, diverse functional groups, and uneven surface charge made proteins more easily influenced by the electric field than polysaccharides[41,45]. Additionally, redox proteins, as the main electroactive components in EPS, played a crucial role in the extracellular electron transfer process[46]. These made proteins more likely to participate in redox reactions after the electric field applied, thereby accelerating the electron transport rate.

-line 373 Besides, Methanosaeta and Methanosarcina, the only methanogen genera with membrane-bound cytochromes[48], may facilitate extracellular electron exchange and DIET process[49]. The abundance of DMER64 genus possessing DIET ability and the cytochrome c-type coding genes related to DIET also increased (Fig.6c and Table.S2). And the apparent electron transfer rate constant (kapp) of the eRAnMBR was higher than the other two reactors (Fig.S17), indicating that the overall extracellular electron transfer performance was strengthened[53]. DIET, considered to be a more efficient electron transfer pathway[54] without relying on the diffusion of electron carriers such as H_2, was conducive to the increase of CH_4 yield in the eRAnMBR.

Reviewer (Comment 2): *Lines 87-93, and figure 1b, COD removal for three different reactors seems similar, with only difference during days 80-120; and what's the cause for such different? does "80 days" mean "more swiftly"? eAnMBR exhibited the same COD removal tendency toAnMBR, and why did the alkali addition from the hydrogen evolution reaction only happen in eRAnMBR but not in eAnMBR with the same posed voltage of 0.6 V?*

Our Response: Thank you very much for your insightful comments and for highlighting these important points. The relevant text has been revised to make this point more rigorous and specific. Regarding your query on whether the "80 days" implies a swifter process, we would like to clarify the observations depicted in the figure. Initially, the hydraulic retention time (HRT) was set as 6 days. This choice led to a rapid degradation of COD in the early stages of the experiment. However, between days 40 and 70, the COD degradation rate showed minimal improvement. After exploring various influencing factors, we concluded that the organic load

might have exceeded the anaerobic bacteria's capacity. Therefore, at day 70, the hydraulic retention time was extended to 8 days[14, 15]. Following this adjustment, the COD removal rate significantly increased. We realized that this explanation was not included in the original manuscript, which likely led to your query and might also confuse the readers.

Regarding your second question, why the alkali addition from the hydrogen evolution reaction only happened in the eRAnMBR but not in the eAnMBR with the same posed voltage of 0.6 V, it is because the eRAnMBR used Mg electrode as the anode while the eAnMBR used graphite electrode under the same voltage conditions (the other anode in both the eAnMBR and eRAnMBR was the membrane anode). From Fig.S3, in the eAnMBR, when the membrane was used as the anode, the current between the membrane anode and graphite cathode reached a maximum of about 0.012A, and when the graphite electrode was used as the anode, the current between the graphite anode and graphite cathode ranged from 0.00756A to 0.00245A. In contrast, in the eRAnMBR system with Mg as the anode, the current between the Mg anode and graphite cathode can reach up to 0.22A (Fig.S4). Even when Mg electrode was not used as an anode, there was still a current above 0.02A between the Mg anode and graphite cathode. This indicated that the electrochemical reactions in the eRAnMBR were much more intense than those in the eAnMBR, primarily due to the presence of the active metal Mg, which made the electrode reactions in the eRAnMBR system more vigorous under the same voltage conditions compared to the eAnMBR system. Additionally, the pH variation graph in the Fig.S2 showed that the pH of the eAnMBR group is not significantly different from the control group, whereas the pH of the eRAnMBR group can reach between 8.2 and 9, also indicating intense hydrogen evolution reactions in this system, resulting in increased alkalinity. The alkalinity produced by the cathodic hydrogen evolution reaction could prevent acid inhibition of the system in the initial stages of the anaerobic process, promoting the conversion of volatile fatty acids (VFA) to CH_4 thus allowing it to complete the four stages of anaerobic reaction earlier[16-18]. The VFA production in the three reactors at days 74, 81, and 86 were also measured. It can be observed that the VFA accumulation in the eRAnMBR system was less compared with the other two reactors (Fig.S5). The relevant content has been added to the article to help readers better understand.

-line 116 As shown in Fig.1b, during the 200 days of operation, the COD concentration gradually decreased and stabilized, with all three reactors demonstrating excellent degradation of organic contaminants, achieving a COD removal rate of 95%. In the first 40 days, the COD degradation rate increased rapidly, but between days 40 and 70, the rate of improvement hardly increased. Therefore, the hydraulic retention time (HRT) was extended from 6 days to 8 days to enhance acclimation efficiency at day 70. After this adjustment, a faster increase in the COD degradation rate of the sludge in the eRAnMBR was observed.

-line 124 This is because, compared to the AnMBR and the eAnMBR, the electrode reactions in the eRAnMBR were more intense (It was concluded from the pH data in Fig.S2 and the current data in Fig.S3, Fig.S4). The alkalinity produced by the cathodic hydrogen evolution reaction could prevent acid inhibition of the system in the initial stages of the anaerobic process (Fig.S5), allowing it to complete the four stages of anaerobic process earlier[21].

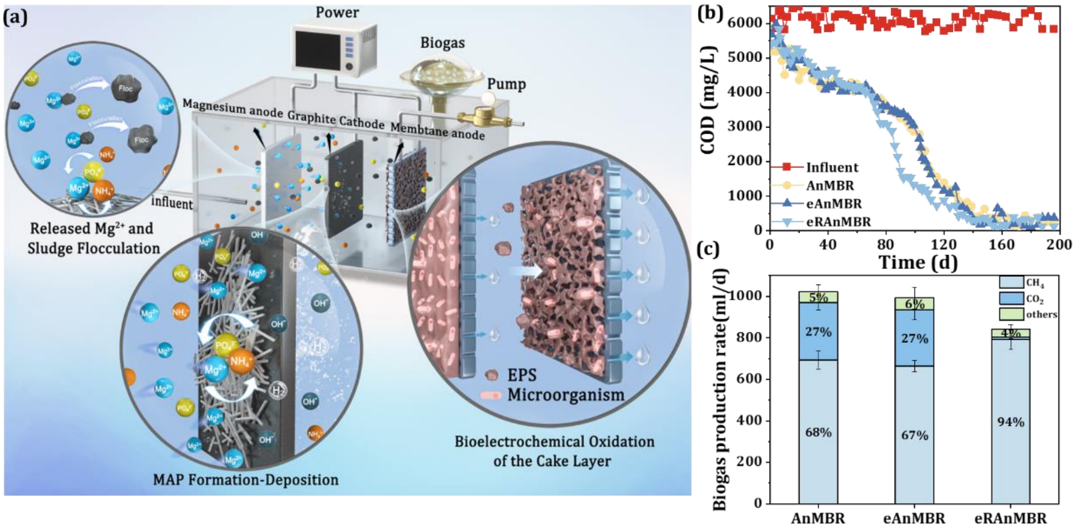

Fig. 1 (a) Mechanisms of the eRAnMBR for struvite recovery and membrane antifouling. ~~Variations in (b) COD, (c) Biogas production and composition in the three groups of AnMBRs during the operation period.~~ (b) Variations in COD in the three reactors of AnMBRs during the operation period. (c) Biogas production rate and biogas composition in the three reactors of AnMBRs during the stable operation period, the percentages of each gas have been rounded to the nearest whole number in the figure (The figure primarily highlights the production rates of the main components of biogas, CH_4 and CO_2. In addition to these two, other gases in biogas we measured were provided in Fig.S7). The anodes of the eAnMBR comprised a conductive membrane and a graphite plate, while the anodes of the eRAnMBR included a conductive membrane and a Mg plate. Applied voltage of both the eRAnMBR and eAnMBR was 0.6 V. COD concentration in the influent was 6 g/L. (Note: the word electro-AnMBR means we mention both the eRAnMBR and eAnMBR).

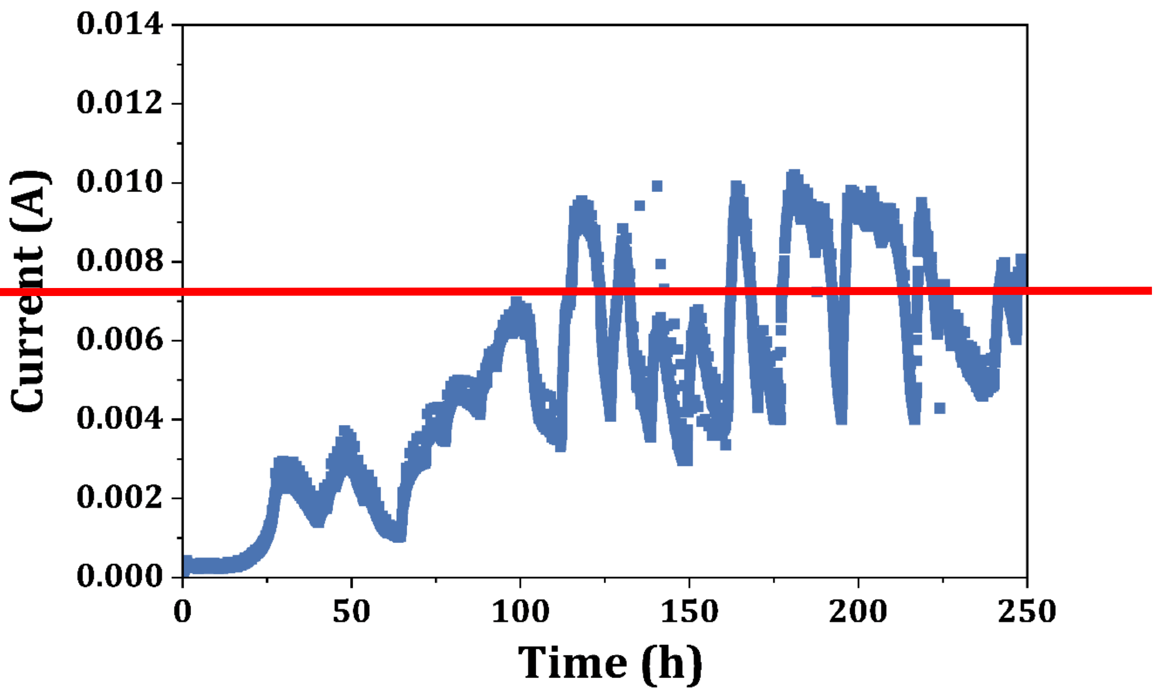

~~Fig.S8 Change of current density of the electro-membrane in eAnMBR after the membrane module renewed.~~

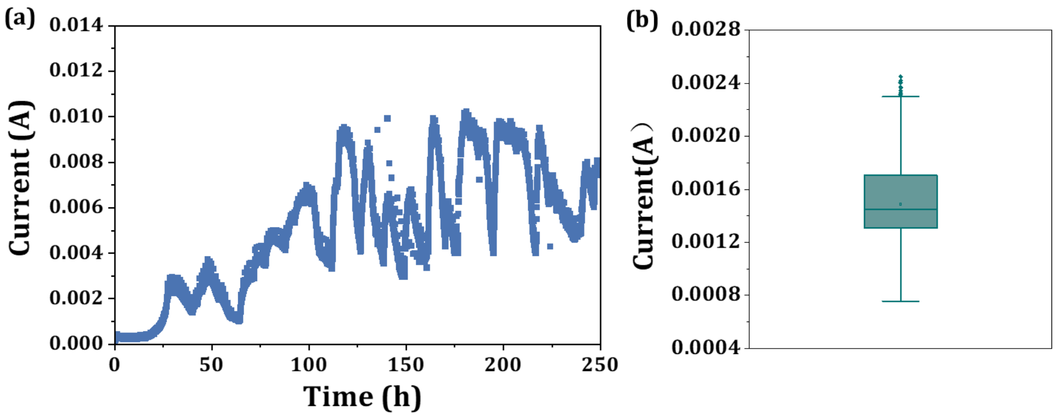

Fig.S3 (a) ~~Change of current density of the electro-membrane in eAnMBR after the membrane module renewed.~~ Change of current between the membrane anode and graphite cathode in the eAnMBR after the membrane module renewed. (b) The current between the graphite anode and graphite cathode when graphite is used as the anode in the eAnMBR.

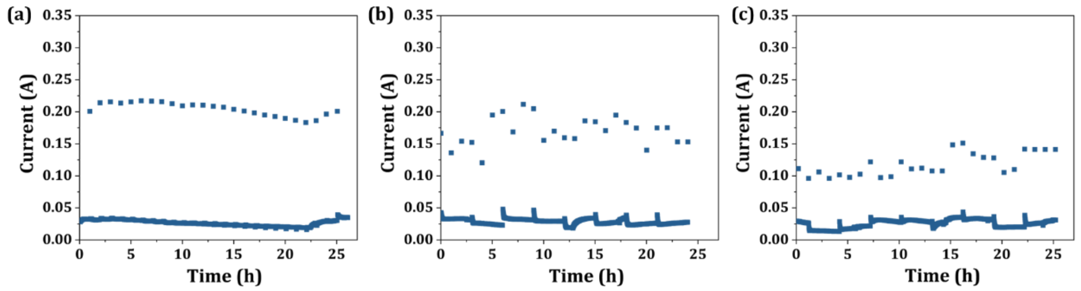

Fig.S4 The current between Mg anode and graphite cathode in the eRAnMBR. The switching time between Mg anode and membrane anode was 1min:1h. The dispersed points at high current were 0.6V voltage applied on the Mg anode, while the densely connected points at low current were 0.6V voltage applied on the membrane anode. The figure showed the current changes at different times after the replacement of Mg anode and graphite cathode. (a) was earlier stage after the replacement of Mg anode and graphite cathode, (b) was middle stage after the replacement of Mg anode and graphite cathode, and (c) was the end stage after the replacement of Mg anode and graphite cathode.

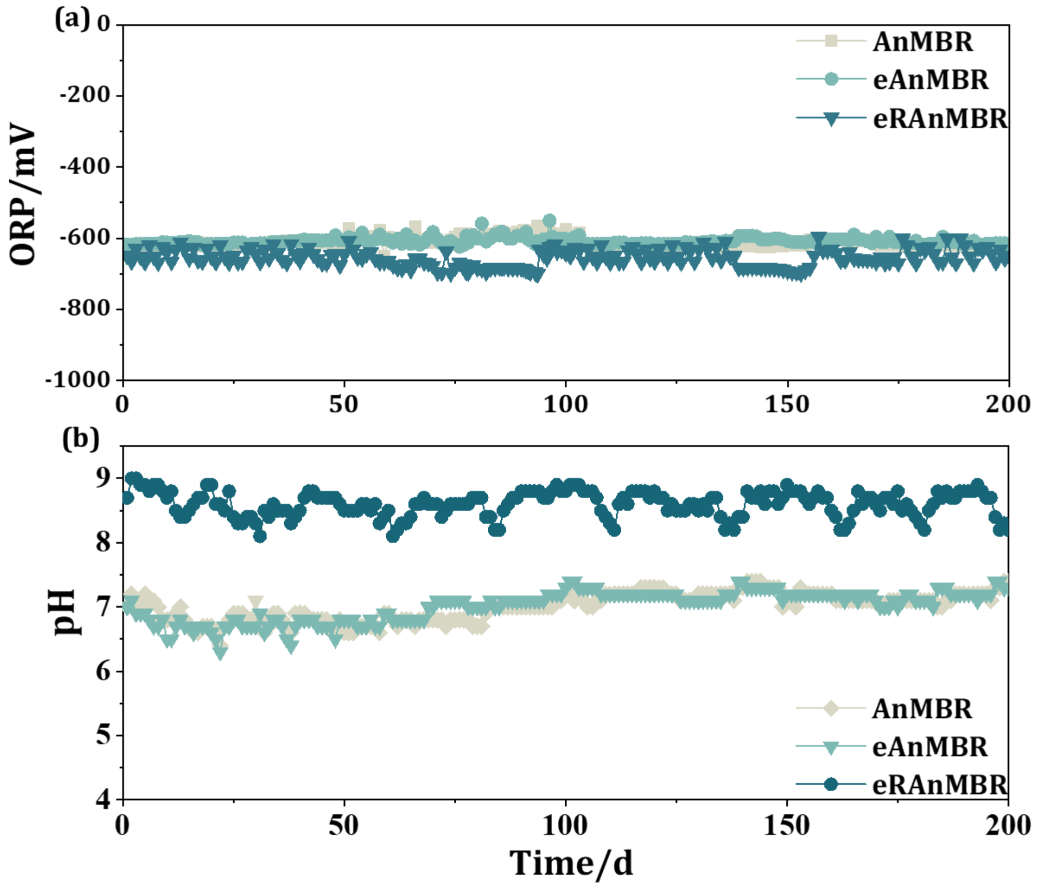

Fig.S2 Variation of (a) ORP and (b) pH of the three AnMBR reactors. The anodes of the eAnMBR comprised a conductive membrane and a graphite plate, while the anodes of the eRAnMBR included a conductive membrane and a Mg plate. Applied voltage of both the eRAnMBR and eAnMBR was 0.6 V. During the acclimation phase (before the COD degradation rate stabilized), if the system pH dropped below 6.5, we added appropriate amount

of sodium bicarbonate ($NaHCO_3$) to prevent acid inhibition, which could lead to system failure.

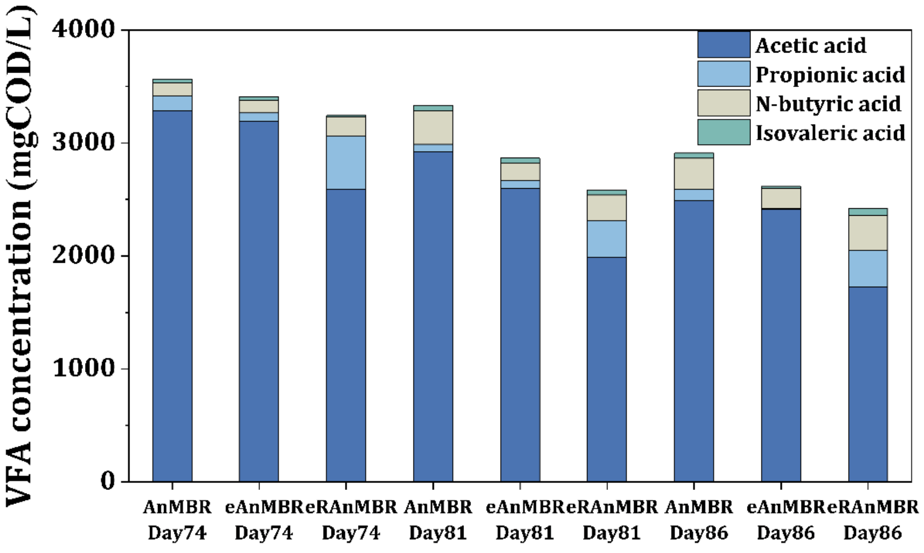

Fig.S5 Volatile fatty acids (VFA) production in the AnMBR, eAnMBR, eRAnMBR, at days 74, 81, and 86 (All the days chosen were after the hydraulic retention time was adjusted from 6 days to 8 days).

Reviewer (Comment 3): *figure 1C, what are the others in biogas with so high content?*

Our Response: Thank you for your valuable question. Supplementary explanations have been added in the original figure caption to aid reader understanding, and data of others gases measured were also provided in Fig.S7. The replacement of membrane modules approximately every two weeks during the experiment (to explore the membrane antifouling mechanism) inevitably allowed atmospheric nitrogen to enter the system (in the headspace above the anaerobic sludge). This led to a high proportion of other gases in our data, reaching around 10%. As a result, additional experiments were conducted to remeasured gas production, extending the membrane module replacement period to avoid nitrogen (N_2) interference. The newly measured results showed a reduction in nitrogen interference, as the proportion of other gases no longer being so high. As shown in Fig.1c, the proportion of other gases in the three reactors is relatively similar, around 5%. In addition to the main gas components in biogas, CH_4 and CO_2, other gases in biogas mainly included hydrogen (H_2), hydrogen sulfide (H_2S), nitrogen (N_2), carbon monoxide (CO), ammonia (NH_3), water vapor (H_2O), hydrocarbons, siloxanes and so on [19-22].

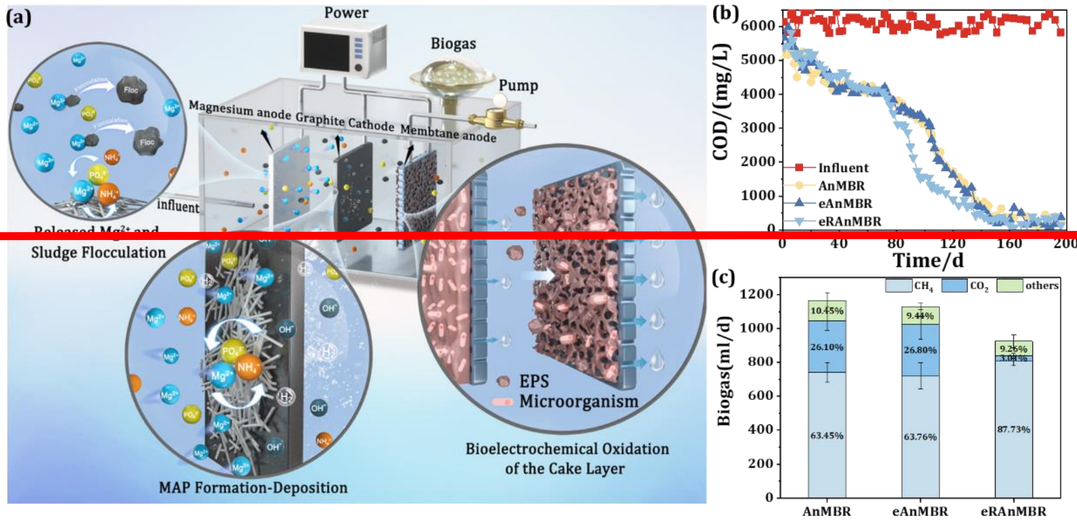

~~Fig.1 (a) Mechanisms of eRAnMBR for struvite recovery and membrane antifouling. Variations in (b) COD, (c) Biogas production and composition in the three groups of AnMBRs during the operation period. The anodes of eAnMBR comprised a conductive membrane and a graphite plate, while eRAnMBR anodes included a conductive membrane and a Mg plate. Applied voltage of both eRAnMBR and eAnMBR was 0.6 V. COD concentration in the influent was 6 g/L. (Note: the word electro-AnMBR means we mention both eRAnMBR and the eAnMBR).~~

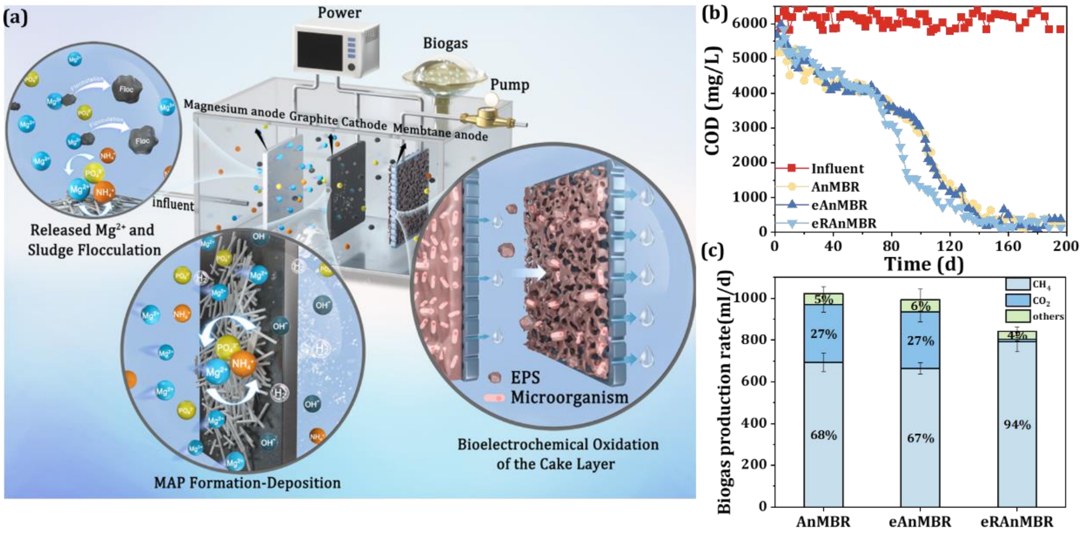

Fig.1 (a) Mechanisms of the eRAnMBR for struvite recovery and membrane antifouling. ~~Variations in (b) COD, (c) Biogas production and composition in the three groups of AnMBRs during the operation period.~~ (b) Variations in COD in the three reactors of AnMBRs during the operation period. (c) Biogas production rate and biogas composition in the three reactors of AnMBRs during the stable operation period, the percentages of each gas have been rounded to the nearest whole number in the figure (The figure primarily highlights the production rates of the main components of biogas, CH_4 and CO_2. In addition to these two, other gases in biogas we measured were provided in Fig.S7). The anodes of the eAnMBR comprised a conductive

membrane and a graphite plate, while the anodes of the eRAnMBR included a conductive membrane and a Mg plate. Applied voltage of both the eRAnMBR and eAnMBR was 0.6 V. COD concentration in the influent was 6 g/L. (Note: the word electro-AnMBR means we mention both the eRAnMBR and eAnMBR).

(a)

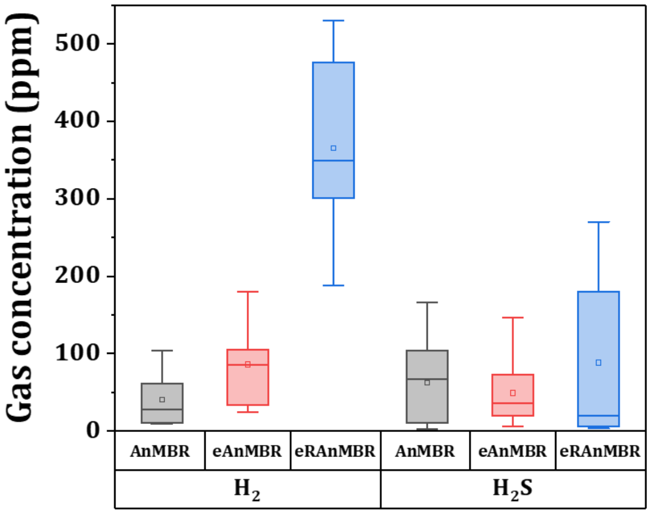

(b)

Gas	N_2	CO	C_2H_6	C_2H_4	C_3H_6	C_3H_8
Proportion	2.1831% ±0.2187%	0.0067% ±0.0028%	0	0	0	0

Fig.S7 Content of other gases in biogas (biogas except for CH_4 and CO_2): (a) Concentration of H_2 and H_2S in biogas in the AnMBR, eAnMBR and eRAnMBR which showed differences during the stable operation period. (b) Proportion of gases which were common in all the three reactors of AnMBRs. In this study, gas was collected using gas bags, making it impossible to avoid the escape of H_2[6-8]. As a result, data of H_2 is lower than the actual value, but it can still be used to compare differences between the three reactors.

Reviewer (Comment 4): *The data regarding methane production rate and biogas composition should be provided.*

Our Response: Yes, methane (CH_4) production rate and biogas composition of the three reactors have been presented in Fig.1(c). The title of the y-axis and the figure legend was modified. Further analysis of the CH_4 production rate and biogas composition was also added in the manuscript. Additionally, data of other gases in biogas were supplemented in Fig.S7, which included H_2S, H_2, N_2, CO and so on[19, 20].

-line 129 From Fig. 1c, the eRAnMBR also exhibited superior carbon recovery performance, with CH4 production rate reaching approximately 790.9 mL/d, higher than the other two groups, which produced about 692.8 mL/d. ~~Furthermore, the proportion of methane in the biogas increased from 63.6% to 87.7%,~~ Furthermore, in the eRAnMBR, the proportion of CO_2 in the biogas decreased from 27.18% to 1.63%, and the proportion of CH_4 in the biogas increased from 67.82% to 93.97%, which was considerably higher than that of previously published reports of AnMBRs[10, 22, 23]. This improvement aided the upgrade to biomethane which can be recovered as natural gas with less treatment[9, 24].

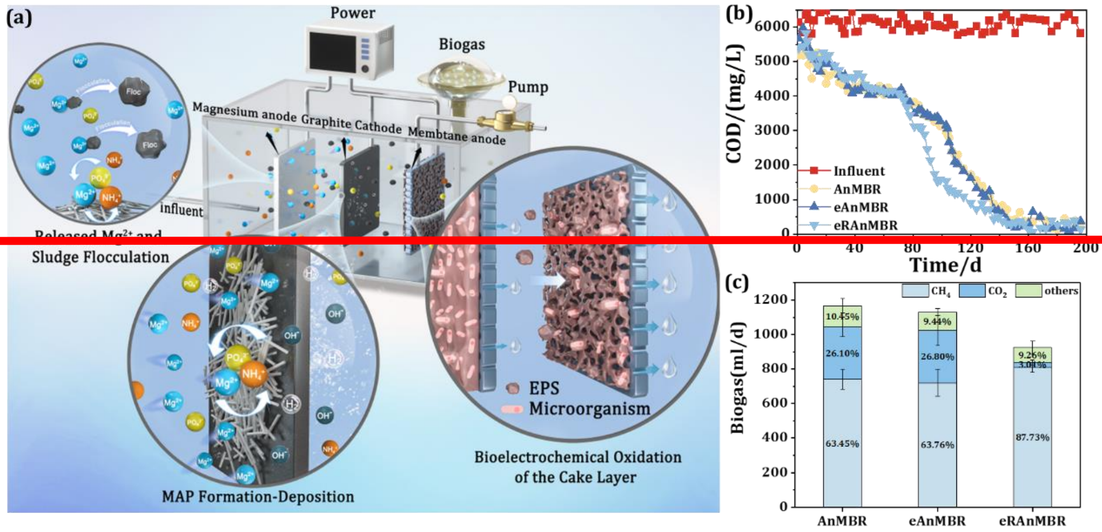

~~Fig.1 (a) Mechanisms of eRAnMBR for struvite recovery and membrane antifouling. Variations in (b) COD, (c) Biogas production and composition in the three groups of AnMBRs during the operation period. The anodes of eAnMBR comprised a conductive membrane and a graphite plate, while eRAnMBR anodes included a conductive membrane and a Mg plate. Applied voltage of both eRAnMBR and eAnMBR was 0.6 V. COD concentration in the influent was 6 g/L. (Note: the word electro-AnMBR means we mention both eRAnMBR and the eAnMBR).~~

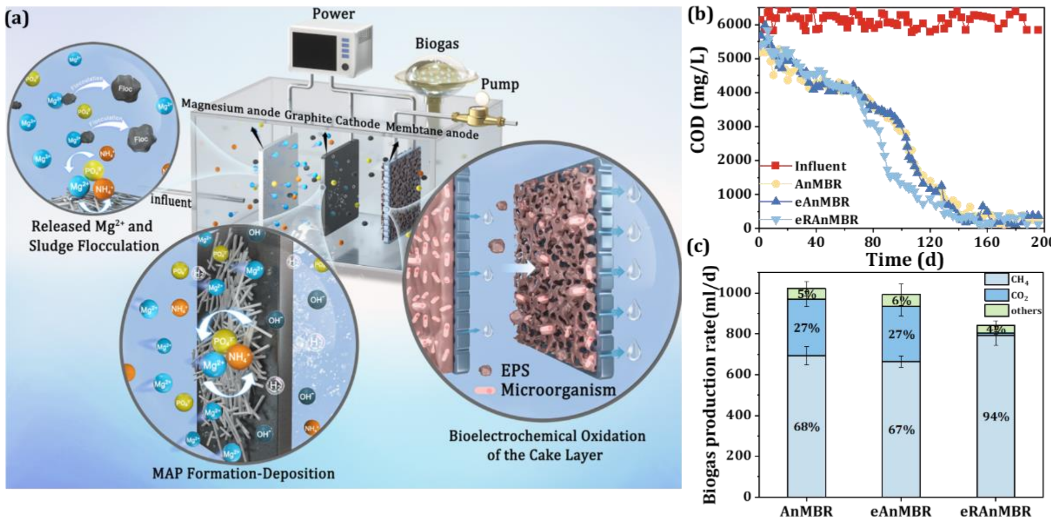

Fig.1 (a) Mechanisms of the eRAnMBR for struvite recovery and membrane antifouling. ~~Variations in (b) COD, (c) Biogas production and composition in the three groups of AnMBRs during the operation period.~~ (b) Variations in COD in the three reactors of AnMBRs during the operation period. (c) Biogas production rate and biogas composition in the three reactors of AnMBRs during the stable operation period, the percentages of each gas have been rounded to the nearest whole number in the figure (The figure primarily highlights the production rates of the main components of biogas, CH_4 and CO_2. In addition to these two, other gases in biogas we measured were provided in Fig.S7). The anodes of the eAnMBR comprised a conductive membrane and a graphite plate, while the anodes of the eRAnMBR included a conductive membrane and a Mg plate. Applied voltage of both the eRAnMBR and eAnMBR was 0.6 V. COD concentration in the influent was 6 g/L. (Note: the word electro-AnMBR means we mention both the eRAnMBR and eAnMBR).

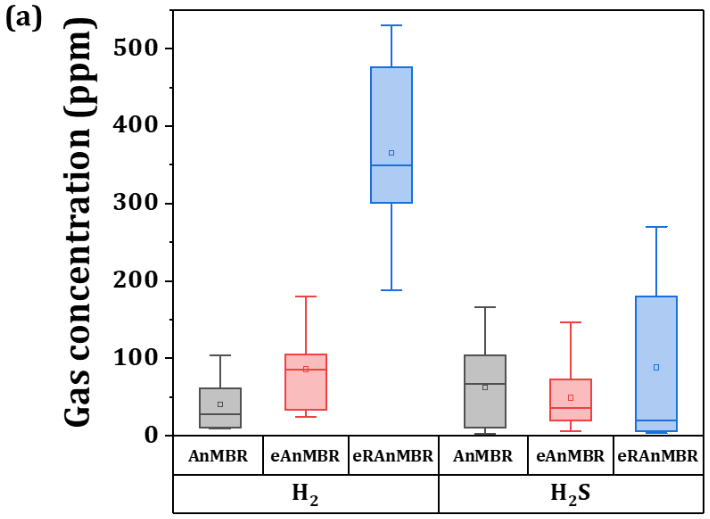

(b)

Gas	N_2	CO	C_2H_6	C_2H_4	C_3H_6	C_3H_8
Proportion	2.1831% ±0.2187%	0.0067% ±0.0028%	0	0	0	0

Fig.S7 Content of other gases in biogas (biogas except for CH_4 and CO_2): (a) Concentration of H_2 and H_2S in biogas in the AnMBR, eAnMBR and eRAnMBR which showed differences during the stable operation period. (b) Proportion of gases which were common in all the three reactors of AnMBRs. In this study, gas was collected using gas bags, making it impossible to avoid the escape of H_2[6-8]. As a result, data of H_2 is lower than the actual value, but it can still be used to compare differences between the three reactors.

Reviewer (Comment 5): *The precipitates that formed on the cathode surface will affect the mass transfer (CO_2) and electron exchange, and how to control such technical issue if it happens?*

Our Response: Thanks for your insightful question. The explanations of the measures we took were supplemented to address this issue to the manuscript, making our argument more rigorous. The precipitates that formed on the cathode surface would affect the mass transfer (CO_2) and electron exchange, and indeed required appropriate control measures. During the operation time, the current between the Mg anode and graphite cathode in the eRAnMBR was monitored (Fig.S4). As the deposits formed and covered the electrodes, the current between he Mg anode and graphite cathode would gradually decrease, which would affect the mass transfer (CO_2) and electron exchange. Therefore, when the current between the Mg anode and the graphite cathode dopped below 0.1A (referred to the current when the 0.6V applied on the Mg plate, current data was displayed in Fig.S4), we replaced the graphite cathode and scraped off the deposits to explore the recovery efficiency of struvite. The current value was derived from preliminary experiments, considering the combined effects of electron exchange and current density on struvite generation[23]. This prevented the deposits on the electrodes from affecting mass transfer (CO_2) and electron exchange, ensuring the system could operate stably. Besides, since the increase in alkalinity due to the hydrogen evolution reaction of graphite cathode increased the solubility of CO_2 in liquid phase, the mass transfer of CO_2 within the liquid phase system increased, reducing the impact of electrode deposits on it[5, 8].

-line 169 The removed nitrogen and phosphorus were recovered as struvite, which partially precipitated on the cathode surface and was separated from the sludge. When the current between the Mg anode and the graphite cathode dropped below 0.1A (referred to the current when the 0.6V applied on the Mg plate, current data was displayed in Fig.S4), both the Mg anode and graphite cathode were replaced, which prevented the deposits on the electrodes from affecting mass transfer and electron exchange as well as ensured sufficient Mg^{2+} throughout the experiment.

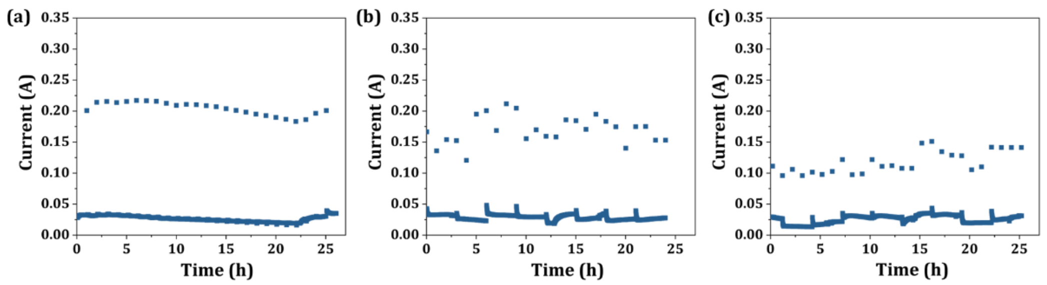

Fig.S4 The current between Mg anode and graphite cathode in the eRAnMBR. The switching time between Mg anode and membrane anode was 1min:1h. The dispersed points at high current were 0.6V voltage applied on the Mg anode, while the densely connected points at low current were 0.6V voltage applied on the membrane anode. The figure showed the current changes at different times after the replacement of Mg anode and graphite cathode. (a) was earlier stage after the replacement of Mg anode and graphite cathode, (b) was middle stage after the replacement of Mg anode and graphite cathode, and (c) was the end stage after the replacement of Mg anode and graphite cathode.

Reviewer (Comment 6): *The concentration of Mg^{2+} ions released into bulk solution should be provided because the size of sacrificial Mg anode will decrease and the release of Mg^{2+} will become slow.*

Our Response: Thank you very much for your suggestion. We overlooked the discussion of this part and indeed should provide the relevant data on the concentration of Mg^{2+} ions released into bulk solution. The figure concerning concentration of Mg^{2+} ions was put into SI (Fig.S8) and relevant content about Mg^{2+} release was supplemented into the main text.

Firstly, in our system, the release amount of Mg^{2+} was not strictly controlled. From Fig.S8, after the released Mg^{2+} have fully combined with NH_4^+ and PO_4^{3-} to form struvite (molar ratio 1:1:1), about 220 mg/L of Mg^{2+} still remained. This was because the Mg^{2+} release process was not just limited to the times when the 0.6V voltage was applied on the Mg plate, there was additional factor that accelerated the corrosion process of Mg. When the conductive membrane acted as the anode, the Mg electrode and graphite electrode formed a galvanic cell, so there was still current flow between them even under open circuit, causing Mg^{2+} to be constantly released. Besides, in the eRAnMBR, the amount of the Mg^{2+} calculated by the Faraday's law was less than the concentration of Mg^{2+} measured, indicating that except the Faraday current, there was also non-Faraday current[24-27]. Supplementary experiments were conducted to verify that the presence of microorganisms enhanced Mg corrosion. This is consistent with the results of previous studies [28, 29]. SBR experiments under both microbial and non-microbial conditions were conducted, with the same synthetic-wastewater distribution as the AnMBRs, without applying voltage, to observe Mg electrode corrosion. From the polarization curves in Fig.9a and Fig.9d, we can see that in the presence of microbial, the Mg plate corroded more easily, and the corrosion current increased. The cyclic

polarization curves in Fig.9b also showed the pitting corrosion on the Mg electrode under microbial conditions, with a lower corrosion potential. Therefore, the release of Mg^{2+} accelerated, resulting in a concentration of 360 mg/L within 3 days in Fig.9c. In summary, there were sufficient Mg^{2+} in the eRAnMBR to ensure the reaction with NH_4^+ and PO_4^{3-} to form struvite.

Indeed, after a long time of the sacrificial Mg anode reaction, the size of the Mg plate would decrease and the corrosion products would gradually cover the surface of Mg and graphite electrodes[23], leading to a drop in current. This reduced the ability of the Mg plate to corrode and release Mg^{2+} ions according to the Faraday's first law (Eq. (1))[30, 31], and also influenced the struvite precipitation on the cathode surface. Therefore, during the operation period of our study, when the current between the Mg anode and the graphite cathode dropped below 0.1A (this 0.1A referred to the current when the 0.6V applied on the Mg plate), both the Mg anode and graphite cathode were replaced to ensure adequate electron transfer and sufficient Mg^{2+} throughout the experiment. Information regarding the replacement of the electrodes were added into the manuscript to ensure that the article is more rigorous and complete.

$$m = \frac{Q \cdot M}{n \cdot F} = \frac{I \cdot t \cdot M}{n \cdot F} \quad (1)$$

(Where: m is the mass of the dissolved or deposited substance, Q is the quantity of electric charge passed through the electrode, M is the molar mass of the metal, n is the number of electrons involved in the reaction, F is the Faraday constant, I is the current and t is the duration of current flow.)

-line 159 As shown in Fig.2a, ammonia nitrogen (NH_4^+) and phosphate (PO_4^{3-}) significantly decreased in the eRAnMBR, with removal rates of 30.59% and 99%, respectively, compared to minimal recovery in the controlled AnMBR and the eAnMBR. In addition to the sacrificial anode reaction induced by a 0.6 V bias applied to the Mg anode, the Mg anode and graphite cathode also formed a galvanic cell when a 0.6 V bias was applied to the membrane anode, leading to the continuous release of Mg^{2+} (Fig. S4). Additionally, in the presence of microorganisms, non-Faradaic currents were generated, which further accelerated the corrosion of Mg (Fig. S8 and Fig. S9). These processes ensured that sufficient Mg^{2+} was available to meet the requirements for struvite formation, resulting in a phosphate removal rate of 99% through the precipitation of magnesium phosphate and struvite.

-line 169 The removed nitrogen and phosphorus were recovered as struvite, which partially precipitated on the cathode surface and was separated from the sludge. When the current between the Mg anode and the graphite cathode dropped below 0.1A (referred to the current when the 0.6V applied on the Mg plate, current data was displayed in Fig.S4), both the Mg anode and graphite cathode were replaced, which prevented the deposits on the electrodes from affecting mass transfer and electron exchange as well as ensured sufficient Mg^{2+} throughout the experiment.

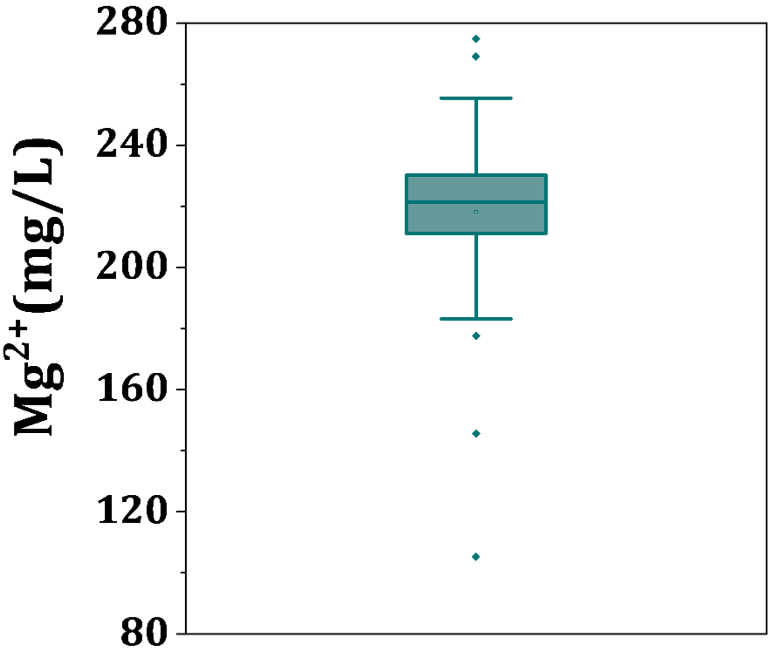

Fig.S8 Variations of Mg^{2+} in the eRAnMBR during the stable operation period.

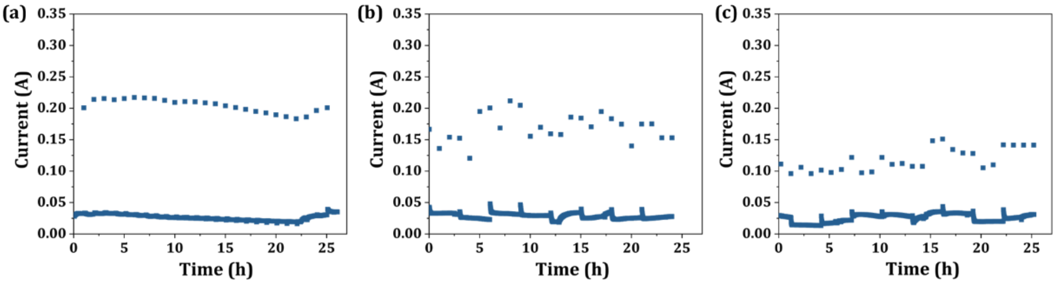

Fig.S4 The current between Mg anode and graphite cathode in the eRAnMBR. The switching time between Mg anode and membrane anode was 1min:1h. The dispersed points at high current were 0.6V voltage applied on the Mg anode, while the densely connected points at low current were 0.6V voltage applied on the membrane anode. The figure showed the current changes at different times after the replacement of Mg anode and graphite cathode. (a) was earlier stage after the replacement of Mg anode and graphite cathode, (b) was middle stage after the replacement of Mg anode and graphite cathode, and (c) was the end stage after the replacement of Mg anode and graphite cathode.

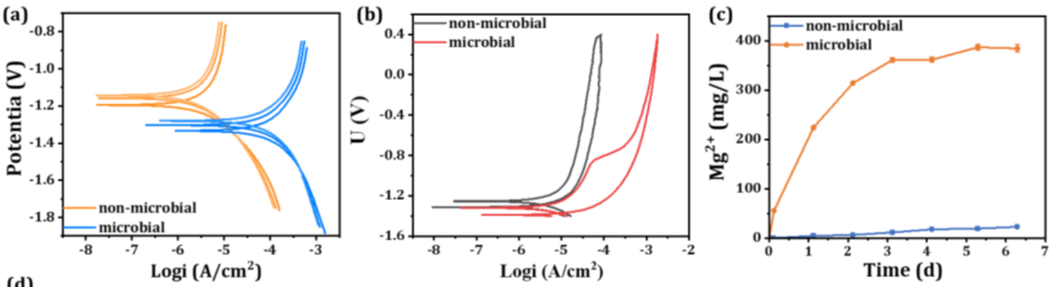

(d)

	icorr(A)	lin pol R(ohm)	Cat slp(1/V)	Ano slp(1/V)	Ecorr(V)
Non-microbial	9.36E-06	4673	6.159	3.931	-1.165
Microbial	2.90E-04	152	5.175	4.797	-1.306

Fig.S9 (a) The polarization curves of the Mg plate under microbial and non-microbial conditions on the third day. (b) The cyclic polarization curves of the Mg plate under microbial and non-microbial conditions on the first day. (c) Concentration of Mg^{2+} released in the system under microbial and non-microbial conditions within 7 days. (d) Corrosion current, linear polarization resistance, cathodic slope, anodic slope and corrosion potential of the Mg plate obtained from the polarization curves under microbial and non-microbial conditions on the third day. All data were based on the SBR reactor, with both microbial and non-microbial conditions and parallel groups. Mg was used as the anode, graphite as the cathode, and Ag/AgCl as the reference electrode. No voltage was applied. The wastewater conditions were consistent with the AnMBRs. The polarization curves and Mg^{2+} were measured over 7 days, with the polarization curves from the third day selected as representative.

Reviewer (Comment 7): *Line 225-230, more explanations should be given for why the sequence of electron disturbance was re-ordered by the applied electric field.*

Our Response: Thanks for your kind suggestion. The explanations regarding why the sequence of electron disturbance was re-ordered by the applied electric field have been supplemented to the manuscript, making the article more rigorous. Compared to polysaccharides, proteins are richer in chemical moieties and contain a large number of amino acid residues, such as cysteine, tyrosine, and tryptophan, which possess functional groups capable of redox reactions[10, 32, 33]. The electron clouds of these groups are more easily influenced by electric fields, leading to faster electron transfer and redox reactions[34-36]. On the other hand, for electroactive microorganisms, redox-active proteins are the main electroactive components in EPS[37, 38]. The protein such as Cytochrome c in EPS correlates well with extracellular electron transfer function[39]. Cytochrome c, the primary electroactive component in electroactive microbial EPS, mediates direct extracellular electron transfer in these microorganisms, contributing up to 80% of the total electron transfer capability in EPS[39, 40]. Therefore, under the condition of the electric field, the electron disturbance in proteins changed more quickly than in polysaccharides.

-line 322 ~~This suggests that the applied electric field facilitated easier electron gain or loss in proteins compared to polysaccharides, making them more likely to engage in redox reactions that accelerate the electron transport rate. This led to a notable decrease in the proportion of proteins and a slight increase in the proportion of polysaccharides in the cake layers of eAnMBR and eRAnMBR.~~ The intricate structure, diverse functional groups, and uneven surface charge made proteins more easily influenced by the electric field than polysaccharides[41,45]. Additionally, redox proteins, as the main electroactive components in EPS, played a crucial role in the extracellular electron transfer process[46]. These made proteins more likely to participate in redox reactions after the electric field applied, thereby accelerating the electron transport rate.

Reviewer (Comment 8): *Line 283, for the energy demand, how about the heating for feedstock, pump for feeding and others?*

Our Response: Thanks for pointing out this issue. We have recalculated the preliminary operating cost of the system and supplemented the detailed calculation process into SI (Text.S3). As the energy required for pumps and thermostatic water baths is necessary for both our new reactor and traditional reactors, these factors were not considered when assessing the economic viability of this novel reactor. However, your question reminded us that the cost of electrode plates, which is a significant expense, should also be included[41, 42]. Based on the calculation of the increased CH_4 production by the eRAnMBR compared to the control AnMBR, as the additional internal energy of the eRAnMBR, the C value calculated is -19.4 USD/m^3 which means the novel eRAnMBR is relatively economical if the CH_4 produced is properly utilized.

Text S3. Preliminary operating cost analysis for eAnRMBR

In this system, many sources of the electric energy consumption (e.g. the pump, the constant temperature water bath and the mixer) and operation costs (e.g.pipeline and membrane replacement) were consistent between the eRAnMBR and conventional AnMBR. Therefore, the additional operating cost of the eRAnMBR compared to conventional AnMBR was composed of energy consumption (electricity) and material consumption (electrode) which drove the electrochemical processes. It was estimated using the following equation.

$$\mathrm{C} = \mathrm{aENC} + \mathrm{bELC} \quad (1)$$

Where, C refers to the additional operating cost, USD/m^3; ENC is the energy consumption, kWh/m^3; ELC is the electrode consumption, kg/ m^3; a is the unit cost of electricity, i.e. 0.102 USD/kWh[1]; and b is the unit cost of the magnesium (Mg) electrode, i.e. 2.56 USD/kg (Because graphite plates are cheap and can be reused during the experiment, we only consider Mg electrode here).

In this system, although additional electrical energy is consumed, the internal methane produced is also a form of energy. Therefore, ENC was calculated as follows:

$$ENC = W_e - W_b \quad (2)$$

The electric energy consumption (W_e, Wh·m^{-3}) was calculated as follows:

$$W_e = \frac{UIt}{3600V} = \frac{UI}{3600Q} \quad (3)$$

Methane was the internal capacity of AnMBR. Under standard conditions, gas production (P_2, Nm^3) was calculated as follows:

$$\frac{P_1V_1}{T_1} = \frac{P_2V_2}{T_2} \quad (4)$$

Biogas energy (methane to electricity, W_b, Wh·m^{-3}) was calculated as follows:

$$W_b = \frac{\varepsilon q V_2 t}{3600V} = \frac{\varepsilon q V_2}{3600Q} \quad (5)$$

where U is the applied potential (V), I is the current (A), t is the running time (h), Q is the flow (m^3 • h^{-1}), P_1 is the working pressure (atm), V_1 is the working biogas production rate (m^3 • h^{-1}), T_1 is 298 K (25 °C), P_2 is one standard atmospheric pressure (atm), V_2 is the standard gas production (Nm^3 • h^{-1}), T_2 is 273 K (0 °C), q is the heat value of the biogas (J • Nm^{-3}), and ε (%) is the methane-to-electricity conversion efficiency with a typical value of 13.779% via recent methane fuel cells[2].

While, the electrode consumption was determined following Faraday's Law[3] as expressed in Eq. (6).

$$\mathrm{ELC} = \frac{\mathrm{IMt}}{\mathrm{nFV}} \quad (6)$$

Where, I is the applied current, A; t is the electrolysis time, h; V is the volume of treated effluent, m^3; M is the molecular weight of iron electrode, g/mol; n is the number of transferred electrons; and F is the Faraday constant, coulomb/mol.

Reviewer (Comment 9): *Line 292, "lowered the hydrophobicity of proteins in EPS" should be "increased", contradictory to the lines 160-164: "Due to the twisted and pleated structure of the β-sheets, the inner hydrophobic groups of amino acids were*

more readily exposed, thus expressing the hydrophobic properties of the sludge flocs. By altering the secondary structure of the EPS proteins, Mg2+ strengthened sludge particle hydrophobicity, thereby reducing sludge viscosity and improving sludge floc aggregation".

Our Response: Thanks for your kind recommendation. We made a typo in the discussion, which led to an inconsistency with the content in the above text. The related statement has been revised to ensure consistency between the discussion and the analysis results. An in-depth analysis of the secondary structure of the proteins was conducted, not just stop at the surface-level conclusions of the secondary structure analysis. Instead, these changes[43, 44] were linked to the formation of a gel layer with higher specific filtration resistance[45] and relative irreversibility[46, 47]. Based on this, revisions have been made as follow:

-line 233 Third, Mg^{2+} changed the secondary structure of protein weakened the formation of gel layer. As a typical negatively charged and sticky substance[4], the contribution of proteins to membrane fouling in the AnMBRs was more than that of polysaccharides in EPS. In comparison to the AnMBR and the eAnMBR, EPS proteins of the eRAnMBR exhibited fewer α-helices and more β-sheets, contributing to the high aggregation capability of the sludge[32,33] (Fig.S13). A lower α-helix/(β-sheet + random coil) ratio suggested that the proteins in the eRAnMBR possessed less hydrogen bonds[35-37] which played a key role in the gel layer[38,39,] thus weakening the formation of a compact foulants layer .

-line 430 The released Mg^{2+} increased floc size, decreased the adhesion force between the sludge particles and the membrane surface, and reduced the proportion of hydrogen bonds in the secondary structure of EPS proteins, thereby weakening the formation of the gel layer and making the cake layer structure loose and porous.

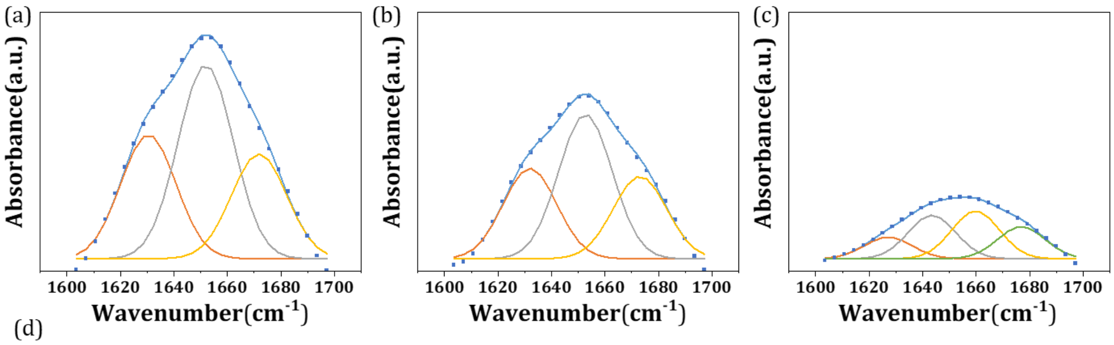

(d)

Sludge	α-helix	β-sheet	Random coil	β-turn	α-Helix/(β-sheet + random coil)
AnMBR	45.56%	28.49%	0.00%	25.96%	1.60
eAnMBR	45.89%	29.30%	0.00%	24.80%	1.57
eRAnMBR	32.97%	14.95%	29.96%	22.13%	0.73

Fig.S13 Second-derivative resolution enhancement and curve-fitted amide I region (1700–1600 cm^{-1}) for EPS proteins from the (a) AnMBR, (b) eAnMBR, and (c) eRAnMBR; (d) Relative content of each secondary structure in EPS proteins for the AnMBR, eAnMBR, and eRAnMBR.

Reviewer (Comment 10): *The findings of this study seems predictable without in-depth insights into either reactor configuration or mechanisms.*

Our Response: Thanks for pointing out this issue. Based on the reviewers' comments, supplemented experiments such as the electrochemical properties characterization of the sludge in the AnMBRs and further mechanistic discussion on the results were conducted. Corresponding text were revised in the article, making the overall study more in-depth. In this study, a novel electrochemical resource-recovery AnMBR was presented, and showed excellent performance in carbon, nitrogen, and phosphorus recovery for the first time. The sacrificial Mg anode enhanced the anaerobic electron transfer and the released Mg^{2+} reduced the adhesion force between the membrane surface and sludge flocs, thereby increasing CH_4 yield and reducing membrane fouling, and nitrogen-phosphorus are converted into struvite which partly deposited on the cathode plate for separation and recovery.

Regarding the eRAnMBR configuration, a dual-anode switching mode was adopted, using conductive membrane and Mg as dual anodes with a switching ratio of 1:60. Compared to continuously applying 0.6V voltage on Mg, this approach significantly reduced the input of Mg electrode and energy consumption. This is because, under a 0.6V applied voltage, the current in the system with Mg as the anode was much higher than when the membrane was used as the anode (Fig.S4), which would lead to a significantly higher energy supply[48]. However, when the membrane anode was connected to the power source (with 0.6V applied), a potential difference was created between the graphite cathode and Mg, which can drive the continuous release of Mg^{2+} without the need for an external applied voltage. This integrated AnMBR was a cylindrical vessel with a magnetic stirrer at the bottom for mixing, and the superior hydraulic conditions ensured thorough reactions within the system, which was superior than the separated AnMBR. The

graphite cathode was positioned between the two anodes, facilitating the migration of Mg^{2+}, NH_4^+ to the cathode. And the hydrogen evolution increased the localized pH at the cathode surface, creating favorable conditions for struvite precipitation, which facilitated the separation of struvite from the sludge for recovery[31, 49]. A removable module was designed at the center of the reactor's top lid, where the electrode plates and membrane modules were fixed. This setup allowed for quick replacement of the membrane modules and electrode plates without disrupting the anaerobic environment (The 3D diagram has been added to Fig.S1).

Regarding the eRAnMBR mechanisms, we emphasized the crucial role of altering the physicochemical properties of sludge in membrane anti-fouling. The released Mg^{2+} increased floc size, decreased the adhesion force between the sludge particles and the membrane surface, and reduced the proportion of hydrogen bonds in the secondary structure of EPS proteins, thereby weakening the formation of gel layer and making the cake layer structure loose and porous. Additionally, eRAnMBR enhanced anaerobic electron transfer and DIET process, thereby increased methane production[50] (Relevant experiments and analysis regarding the electron transfer have been supplemented in the manuscript, Fig.S17 and Table.S2). And the cathodic hydrogen evolution reaction enhanced the absorption of CO_2 from biogas into the liquid phase and provided H_2, thus achieving in-situ biogas upgrading. Besides, the application of the electric field also enhanced the microbial anodic oxidation, and made proteins more likely to participate in redox reactions.

-line 233 Third, Mg^{2+} changed the secondary structure of protein weakened the formation of gel layer. As a typical negatively charged and sticky substance[4], the contribution of proteins to membrane fouling in the AnMBRs was more than that of polysaccharides in EPS. In comparison to the AnMBR and the eAnMBR, EPS proteins of the eRAnMBR exhibited fewer α-helices and more β-sheets, contributing to the high aggregation capability of the sludge[32,33] (Fig.S13). A lower α-helix/(β-sheet + random coil) ratio suggested that the proteins in the eRAnMBR possessed less hydrogen bonds[35-37] which played a key role in the gel layer[38,39], thus weakening the formation of a compact foulants layer.

Consequently, the eRAnMBR cake layer exhibited a larger BET specific surface area and total pore volume, with porosity reaching 7.47% (10 times higher than that of the control group), ~~which greatly facilitated membrane fouling mitigation and water permeation.~~ The released Mg^{2+} improved the physicochemical properties of sludge flocs making which difficult to deposit or adhere to the membrane surface, thus forming a loose porous cake layer structure, which greatly improved the service life of the membrane.

-line 327 The intricate structure, diverse functional groups, and uneven surface charge made proteins more easily influenced by the electric field than polysaccharides[41,45]. Additionally, redox proteins, as the main electroactive components in EPS, played a crucial role in the extracellular electron transfer process[46]. These made proteins more likely to participate in redox reactions after the electric field applied, thereby accelerating the electron transport rate.

-line 373 Besides, Methanosaeta and Methanosarcina, the only methanogen genera with membrane-bound cytochromes[48], may facilitate extracellular electron exchange and DIET process[49]. The abundance of DMER64 genus possessing DIET ability and the cytochrome c-type coding genes related to DIET also increased (Fig.6c and Table.S2). And the apparent electron transfer rate constant (kapp) of the eRAnMBR was higher than the other two reactors (Fig.S17), indicating that the overall extracellular electron transfer performance was strengthened[53]. DIET, considered to be a more efficient electron transfer pathway[54] without relying on the diffusion of electron carriers such as H_2, was conducive to the increase of CH_4 yield in the eRAnMBR. In addition, Methanosarcina produced less EPS than Methanobacterium[51] which can help alleviating membrane fouling.

-line 452 A removable module was designed at the center of the reactor's top lid, where the electrode plates and membrane modules were fixed. This setup allowed for quick replacement of the membrane modules and electrode plates without disrupting the anaerobic environment (Fig.S1).

-line 395 Discussion

To strengthen energy and resource recovery as well as membrane anti-fouling, a novel eRAnMBR featuring dual anodes—an Mg anode and a membrane-anode—was developed. Notably, the eRAnMBR system outperformed traditional AnMBRs, removing 30.59% of NH_4^+ and 99% of PO_4^{3-}, while producing struvite with 60.64% purity and depositing 0.08 g/d on the cathode surface for easy separation from the sludge. The eRAnMBR system also upgraded biogas to biomethane, significantly increasing its calorific value and facilitating its recovery as natural gas. ~~Considering the conversion efficiency of methane to electricity, the average energy production capacity of the eRAnMBR was 883.35 Wh/m^3, far exceeding the energy demand (0.015 Wh/m^3), demonstrating the feasibility of using self-sufficient power for sewage treatment.~~ The operating cost analysis indicated that the average energy production capacity of the eRAnMBR, exceeded the additional energy and electrode material consumption, demonstrating the economic viability of this configuration (Text.S3). The enhancement of DIET and all methanogenesis pathways within the system, along with the H_2 evolution and alkalinity production after the application of the Mg anode, all contributed to the increase in CH_4 purity and yield. ~~Regarding antifouling performance, the eRAnMBR achieved a 95% reduction compared to traditional AnMBRs, primarily attributed to two reasons. First, the release of Mg^{2+} altered the physicochemical properties of the sludge, generating large flocs and reducing the adhesion force between the membrane surface and sludge, thus slowing the formation of an irreversible cake layer. Second, the strength of microbial anodic oxidation decomposed the proteins in the cake layer after applying anodic potential to the conductive membrane. The eRAnMBR reduced the secretion and lowered the hydrophobicity of proteins in EPS, and made them more likely to engage in redox reactions that accelerate the electron transport rate. Furthermore, the increased relative abundance of *DMER64* and *Methanosarcina* indicated enhanced direct interspecific electron transfer following the application of the electric field, and all methanogenesis-related modules were strengthened in the eRAnMBR, thus promoting methane production efficiency. The eRAnMBR introduces a novel approach to anaerobic digestion for more sustainable wastewater treatment. Future research should focus on improving the proportion of struvite deposited on the cathode surface for easier nutrient recovery, and on optimizing the use of collected methane to offset the greenhouse gas footprint, thereby maximizing the application potential of integrated AnMBR systems.~~ In-depth research on the physicochemical properties of the sludge explained the 95% reduction in membrane fouling of the eRAnMBR. The released Mg^{2+} increased floc size, decreased the adhesion force between the sludge particles and the membrane surface, and reduced the proportion of hydrogen bonds in the secondary structure of EPS proteins, thereby weakening the formation of the gel layer and making the cake layer structure loose and porous. The application of the electric field also enhanced the microbial anodic oxidation, and made proteins more likely to participate in redox reactions.

In this study, it was found that alleviating membrane fouling by regulating the cake layer through altering the physicochemical properties of the sludge was far more effective than the singular electrodynamic effect. This provided a new approach for extending membrane lifespan in future applications. The new configuration, which switched between the membrane and Mg anodes, reduced Mg material input while effectively recovering three types of resources, enabling the application of AnMBRs in a more sustainable wastewater treatment. As for industrial applications, more economical anode materials, electrode configurations that highly facilitate mass transfer and struvite precipitation, and more efficient utilization of the generated CH_4 to reduce costs are engineering considerations for the practical application of this reactor.

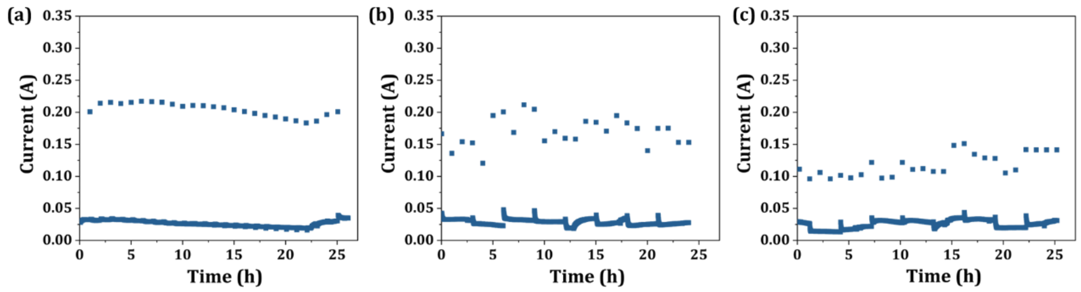

Fig.S4 The current between Mg anode and graphite cathode in the eRAnMBR. The switching time between Mg anode and membrane anode was 1min:1h. The dispersed points at high current were 0.6V voltage applied on the Mg anode, while the densely connected points at low current were 0.6V voltage applied on the membrane anode. The figure showed the current changes at different times after the replacement of Mg anode and graphite cathode. (a) was earlier stage after the replacement of Mg anode and graphite cathode, (b) was middle stage after the replacement of Mg anode and graphite cathode, and (c) was the end stage after the replacement of Mg anode and graphite cathode.

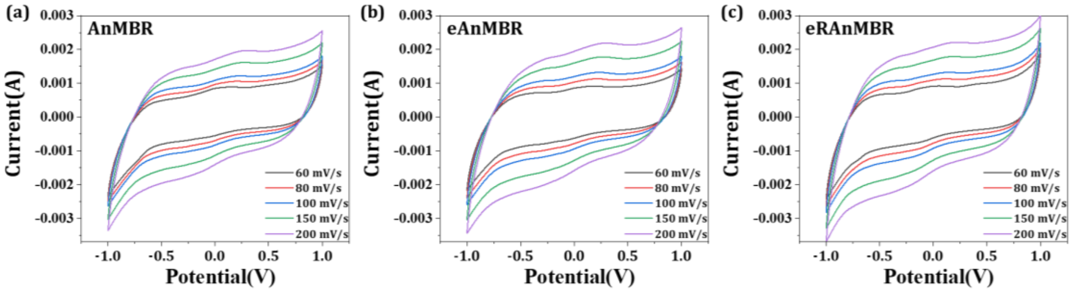

(d)

	AnMBR	eAnMBR	eRAnMBR
k_{app} (s^{-1})	0.2021	0.2009	0.2569

Fig.S17 Cyclic voltammetry (CV) of sludge sampled from the (a) AnMBR, (b) eAnMBR, (c) eRAnMBR measured at scan rates of 60, 80, 100, 150 and 200 mV/s. (d) The apparent electron transfer rate constant (kapp, s^{-1}) of the sludge in the three reactors. CV curves were constructed using a three-electrode system (CHI660, Chenhua Instrument, China), in which Ag/AgCl served as the reference electrode, a graphite plate served as the working electrode, and a platinum sheet served as the opposing electrode. The sludge samples were taken from the three reactors during the stable operation period. The apparent electron transfer rate constant (kapp, s−1) was calculated based on the Laviron equation[9].

Table.S2 Cytochrome c gene counts of the microorganisms in sludge bulk solution of the AnMBR, eAnMBR and eRAnMBR.

KO	KEGG Name	KO Description	Gene_Count		
			AnMBR	eAnMBR	eRAnMBR
K02197	ccmE	cytochrome c-type biogenesis protein CcmE	23	23	27
K02198	ccmF	cytochrome c-type biogenesis protein CcmF	69	59	82
K02199	ccmG, dsbE	cytochrome c biogenesis protein CcmG, thiol:disulfide interchange protein DsbE	179	179	197
K02200	ccmH	cytochrome c-type biogenesis protein CcmH	6	7	15
K06196	ccdA	cytochrome c-type biogenesis protein	178	161	211
K07399	resB, ccs1	cytochrome c biogenesis protein	41	41	45
K08738	CYC	cytochrome c	48	41	54
K26318	fccA, fcc3	fumarate reductase (cytochrome) [EC:1.3.2.4]	15	18	22
Total			559	529	653

Reviewer #3 (Remarks to the Author):

<u>Reviewer</u>: *This paper investigates a novel resource-recovery anaerobic membrane bioreactor and delves into its mechanism. The work is intriguing, but I believe there are still many issues that need to be addressed to meet publication standards. My comments are listed below:*

Our Response: We thank the reviewer for pointing out the interesting insights of our study. All specific comments/questions have been addressed as below.

<u>Reviewer (Comment 1)</u>: *Fig.4b: What is the control membrane and electro-membrane?*

Our Response: Thanks for pointing out this issue. The figure caption of Fig.4b was supplemented to provide a more comprehensive description of the results. The control membrane was taken from the control AnMBR, while the electro-membrane was taken from the eAnMBR. The redox activity of microorganisms on the membrane surface were characterized after applying the anode potential by comparing these two membranes.

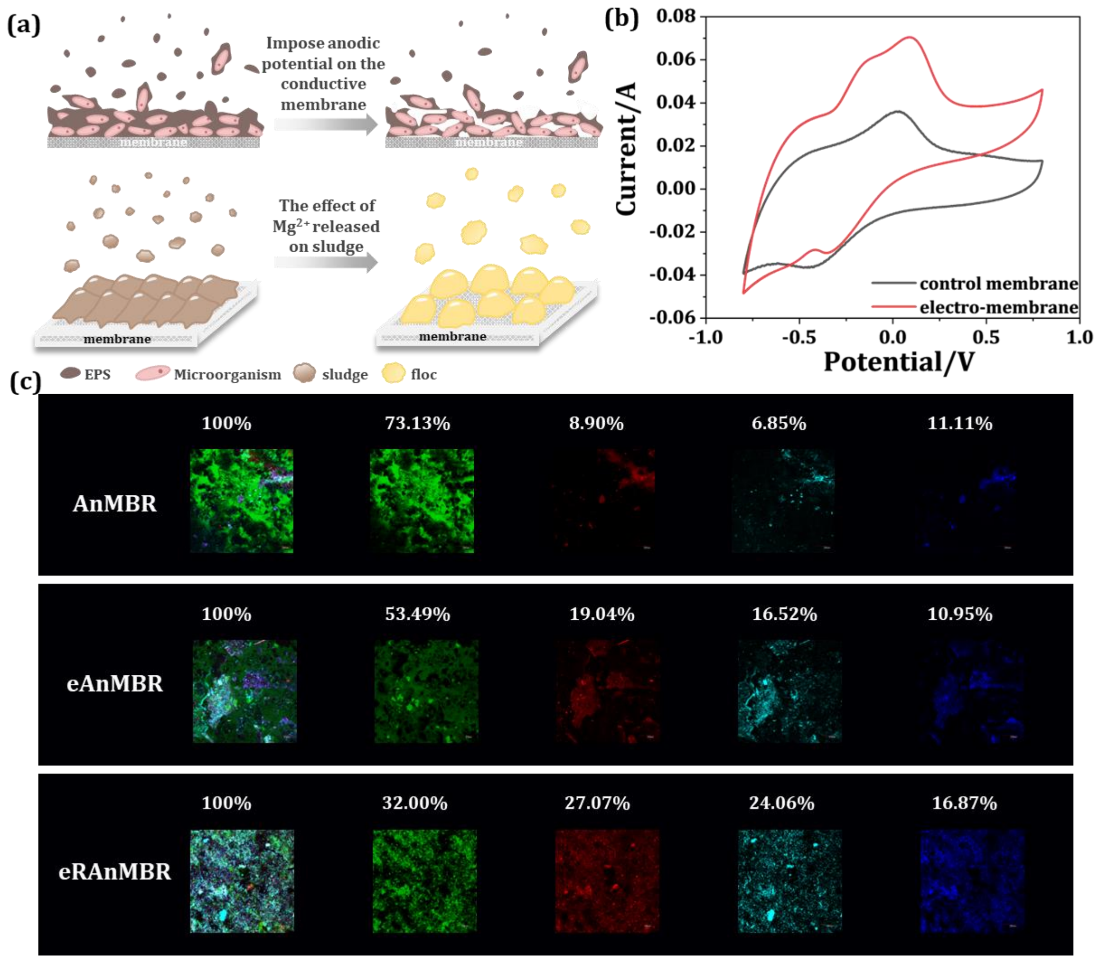

Fig. 4 (a) ~~Effects of Mg on sludge and anode potential on membrane fouling alleviation.~~ (a) Membrane fouling mitigation of the eRAnMBR: effects of anode potential on the cake layer and Mg^{2+} released on sludge. (b) Cyclic voltametric analyses of control membrane and electro-membrane in the 0.8 to −0.8 V range at scan rates of 2 mV/s using a three-electrode system, with Ag/AgCl as the reference electrode, conductive membrane as the working electrode, and graphite cathode as the opposing electrode. Control membrane was taken from the AnMBR, and electro-membrane was taken from the eAnMBR. (c) CLSM images of membrane surface of three reactors (total cells are in red, proteins are in green, α-polysaccharides are in purple, and β-polysaccharides are in blue).

<u>Reviewer (Comment 2)</u>: *Fig.2c: I noticed the same image appearing in both Fig.2 and Fig.S3, but they appear inconsistent. Please check.*

Our Response: Thanks for pointing out this issue. This inconsistence was caused by the different ranges of the vertical axis. To ensure consistency in data presentation, the vertical axis ranges was adjusted in Fig.S3(now is Fig.S10) to be consistent with the same figure in the main text.

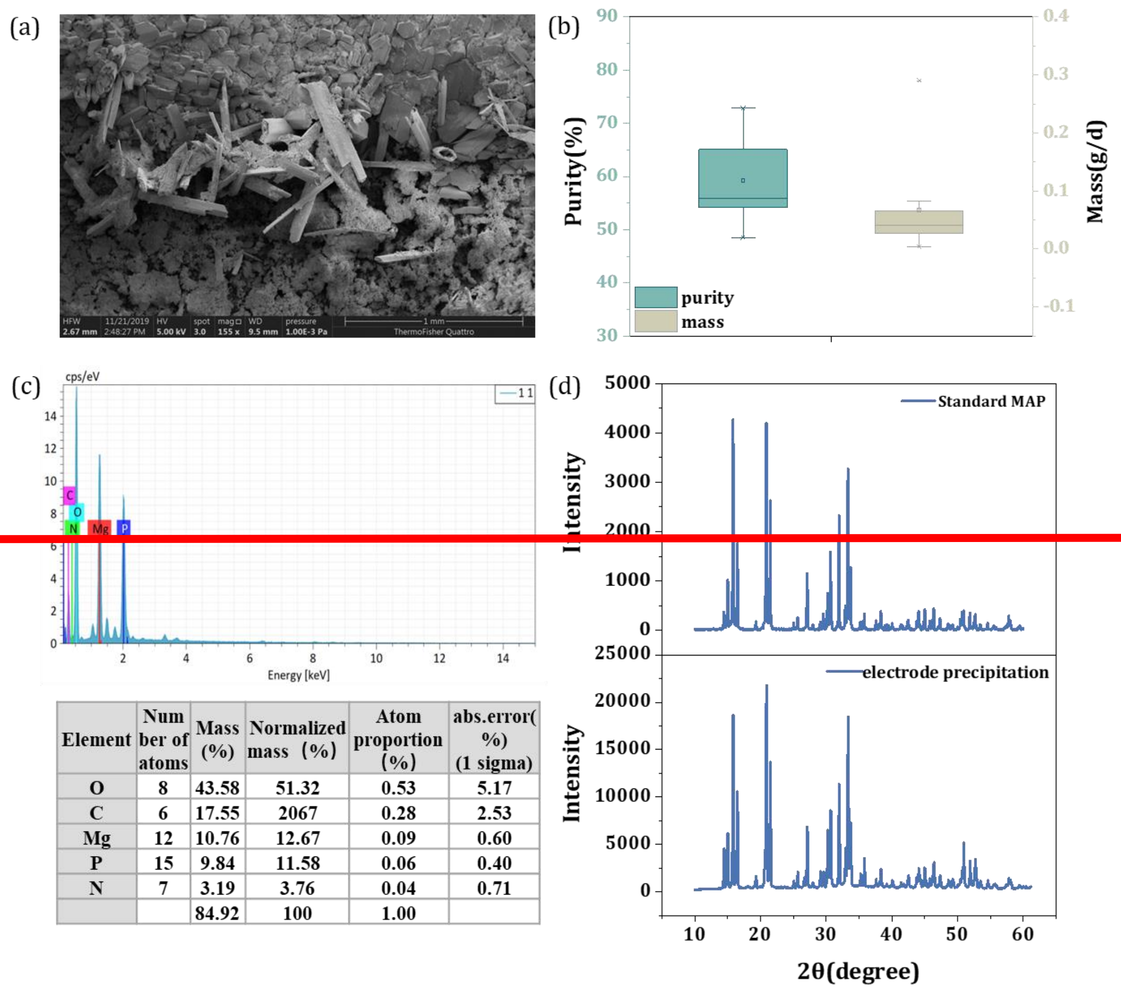

Element	Number of atoms	Mass (%)	Normalized mass (%)	Atom proportion (%)	abs.error(%) (1 sigma)
O	8	43.58	51.32	0.53	5.17
C	6	17.55	2067	0.28	2.53
Mg	12	10.76	12.67	0.09	0.60
P	15	9.84	11.58	0.06	0.40
N	7	3.19	3.76	0.04	0.71
		84.92	100	1.00	

~~Fig.S3 (a) SEM images of the formed struvite on cathode surface (The measuing scale is 1 mm). (b) Mass and purity of the struvite deposited on cathode plate during the stable period. (c) EDS~~

~~images and the elementary composition of the precipitations deposited on cathode. (d) XRD spectra of the precipitations deposited on cathode.~~

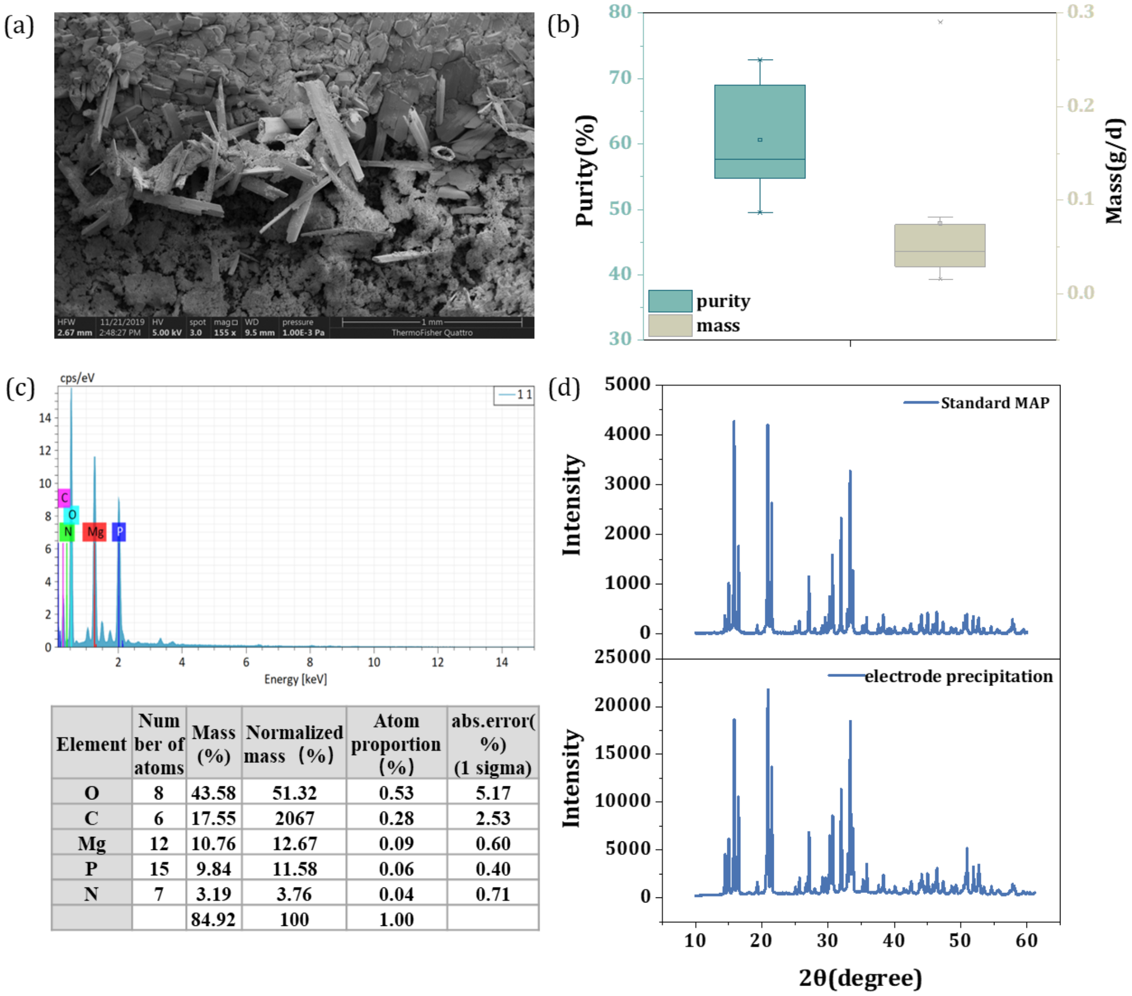

Element	Number of atoms	Mass (%)	Normalized mass (%)	Atom proportion (%)	abs.error(%) (1 sigma)
O	8	43.58	51.32	0.53	5.17
C	6	17.55	2067	0.28	2.53
Mg	12	10.76	12.67	0.09	0.60
P	15	9.84	11.58	0.06	0.40
N	7	3.19	3.76	0.04	0.71
		84.92	100	1.00	

Fig.S10 (a) SEM images of the formed struvite on cathode surface (The measuing scale is 1 mm). (b) Mass and purity of the struvite deposited on cathode plate during the stable period. (c) EDS images and the elementary composition of the precipitations deposited on cathode. (d) XRD spectra of the precipitations deposited on cathode.

Reviewer (Comment 3): *Section "Microbial anodic oxidation and its effect on EPS": "These results confirmed that microbial anodic oxidation was strengthened, leading to the decomposition of complex organics in the cake layer of the membrane-anode" However, the author only focused on proteins in the explanation of CLSM images. It should be noted that complex organics are not limited to proteins alone.*

Our Response: Thanks for pointing out this issue. After discussing the decomposition of complex organic matter, we focused solely on proteins when explaining the CLSM results, which is indeed not rigorous enough. The entire section has been revised to align with the research logic and to ensure more precise expression. In fact, in this part of the experimental design, the distribution of organic matter on the membrane surface was firstly measured, and then, based on the observed results, the underlying electrochemical mechanisms were further explored. Therefore, the logical flow in the manuscript needed refinement to better convey our research. This part has been revised, starting with the observation that applying an electric field resulted in a decrease in proteins on membrane and an increase in the proportion of active cells (with this effect being more pronounced in the eRAnMBR). We hypothesized that this could be due to the enhanced electron transfer capabilities of microorganisms on the membrane surface[35, 51]. To further validate our hypothesis, biofilm electrochemical characterization was conducted, analyzed membrane anode current changes, and examined changes in genes related to extracellular electron transfer in microorganisms on the membrane surface. The microbial anodic oxidation capability of microorganisms on the membrane surface was enhanced, leading to the degradation of proteins[52-54]. Compared to polysaccharides, proteins have a greater impact on membrane fouling, and their reduction played a significant role in alleviating membrane fouling[43, 55]. In the subsequent section, the EPS around the membrane were extracted and used 2D-correlation spectroscopy to further investigate the effect of electric field on EPS forming membrane fouling.

-line 258 To investigate the mitigation mechanisms of membrane fouling following the application of anodic potential to the membrane, ~~we further explored the electrochemical performance and fouling distribution of the cake layer~~ fouling distribution of the cake layer and electrochemical performance were further explored. ~~Results showed that the current density between the anode and cathode increased due to anodic oxidation driven by exoelectrogens (Fig.S7)[34]. The cyclic voltammogram (CV) curves also indicated that the sludge cake layer on the membrane-anode exhibited markedly higher electron-storage capacity and peak oxidation current compared to the control membrane. The cytochrome c-type coding genes of microorganisms on the membrane surface in the AnMBR and eRAnMBR, associated with bioelectrochemical oxidation[35], were aligned. As detailed in Table S1, total cytochrome c coding genes increased by 14%, from 299 on the control membrane of AnMBR to 341 on the electro-membrane of the eAnMBR, notably due to the ccm proteins involved in post translational modifications essential for Mtr pathway protein production[36]. These results confirmed that microbial anodic oxidation was strengthened, leading to the decomposition of complex organics in the cake layer of the membrane anode[37,38]. Furthermore,~~ The Confocal laser-scanning microscopy (CLSM) images showed that while protein intensity was very high in the AnMBR, it significantly decreased after the application of anodic potential imposed on the membrane (Fig.4c). In contrast, total cell intensity increased from 8.90% to 19.04% following the imposition of anodic potential on the membrane, indicating ~~protein degradation and electro-biofilm formation~~ the formation of electro-biofilm. The images also revealed that proteins in the AnMBR tightly and thickly covered the membrane surface, whereas proteins in the membrane-anode showed a much looser structure, facilitating water permeation. Consequently, the roughness of the cake layer increased after the anodic potential was applied on the membrane (Fig.S14).

The mechanisms behind the aforementioned results were further investigated. The cyclic voltammogram (CV) curves in Fig.4b showed that the sludge cake layer on the membrane-anode exhibited markedly higher electron-storage capacity and peak oxidation current compared to the control membrane, indicating the enhancement of microbial anodic oxidation. And the cytochrome c-type coding genes associated with bioelectrochemical oxidation[41] of microorganisms on the membrane surface also increased by 14%, from 299 on the control membrane of AnMBR to 341 on the electro-membrane of the ~~eRAnMBR~~eAnMBR (Table S1), especially the ccm proteins involved in post-translational modifications essential for Mtr pathway protein production[42]. Therefore, the current density between the anode and cathode increased driven by exoelectrogens (Fig.S3a)[40]. These results confirmed that microbial anodic oxidation was strengthened after the imposition of anodic potential on the membrane, conductive to the degradation of proteins in the cake layer of the membrane-anode[43,44]. Given the higher contribution of proteins to membrane fouling in AnMBRs than polysaccharides[4], the degradation of proteins was more beneficial for the mitigation of membrane fouling.

<u>Reviewer (Comment 4)</u>: *Section "Antifouling performance and optimization of cake layer structure": The analysis of protein secondary structure quite interesting, but the correlation with membrane fouling is inadequately described. It is necessary for the author to improve their explanation of this aspect of the mechanism.*

Our Response: We sincerely appreciated your interest in our research and your valuable feedback. We have revised the entire paragraph to present a more logical explanation. In this section, regarding the introduction of the Mg plate for mitigating membrane fouling, extensive characterization experiments were conducted. Firstly, the changes of TMP among the three reactors were presented. Secondly, mechanisms behind the significant mitigation of membrane fouling in the eRAnMBR system were analyzed. Specifically: (1) The sludge viscosity as well as the adhesive forces between the sludge particles and the membrane anode reduced. (2) The floc particle size increased. (3) Secondary structure of sludge proteins was conducted[56], exploring how changes in secondary structure influenced the structural changes in membrane fouling within the eRAnMBR, including the gel layer and cake layer. In this section, an in-depth analysis of the secondary structure of the proteins was conducted, not just stop at the surface-level conclusions of the secondary structure analysis. Instead, these changes[44, 57] were linked to the formation of a gel layer with higher specific filtration resistance[45] and relative irreversibility[46, 47]. Lastly, the analysis of the structural characteristics of the formed cake layer were supplemented and a summary of the entire section was provided.

-line 203 ~~Membrane fouling in the eRAnMBR was significantly alleviated due to improvements in the physical and chemical properties of the sludge floc.~~ Membrane fouling was also significantly alleviated in the eRAnMBR. As illustrated in Fig.3a, under identical effluent flux conditions, the transmembrane pressure (TMP) of the AnMBR reached approximately 80 kPa, while the TMP of the eAnMBR and the eRAnMBR were approximately 64 kPa and 4 kPa, respectively. ~~These findings indicated that membrane fouling was reduced by 20% and 95%, respectively~~ Membrane fouling was reduced by 95% in the eRAnMBR, substantially extending the life span of the membrane, indicating the key role on membrane fouling mitigation of Mg anode.

First, the presence of Mg^{2+} decreased the viscosity of the sludge (Fig.S12b), potentially weakening the adsorption force between the membrane surface and sludge flocs, as highly viscous sludge flocs tend to accumulate on the membrane surface[29]. This effect was confirmed by the images of Atomic force microscopy (AFM) [30], which showed a decrease in adhesion force from 13.15 nN to 4.94 nN, thereby slowing the formation of the cake layer[31]. ~~The amide I region of the Fourier transform infrared spectroscopy (FTIR) spectra of extracellular polymeric substances (EPS) was analyzed to assess the protein secondary structure. In comparison to the AnMBR and eAnMBR, the eRAnMBR EPS proteins exhibited fewer α-helices and more β-sheets, contributing to the high aggregation capability of the sludge[29] (Fig. S6). Due to the twisted and pleated structure of the β-sheets, the inner hydrophobic groups of amino acids were more readily exposed, thus expressing the hydrophobic properties of the sludge flocs[30]. By altering the secondary structure of the EPS proteins, Mg^{2+} strengthened sludge particle hydrophobicity, thereby reducing sludge viscosity and improving sludge floc aggregation[31].~~ Second, the size of the sludge flocs increased in the eRAnMBR (Fig.3d), and X-ray photoelectron spectroscopy (XPS) analysis confirmed the existence of $Mg(OH)_2$. Mg hydroxide coagulation primarily occurred through charge neutralization and adsorptive mechanisms, leading to floc formation[34]. ~~The positively charged colloidal particles of Mg hydroxide act as condensation nuclei, adsorbing negatively charged organic matter and thus increasing the floc particle size[31].~~ The decrease in absolute zeta potential of sludge (Fig.S12a) in the eRAnMBR further confirmed the effect of Mg^{2+} on sludge floc agglomeration. Third, Mg^{2+} changed the secondary structure of protein weakened the formation of gel layer. As a typical negatively charged and sticky substance[4], the contribution of proteins to membrane fouling in the AnMBRs was more than that of polysaccharides in EPS. In comparison to the AnMBR and the eAnMBR, EPS proteins of the eRAnMBR exhibited fewer α-helices and more β-sheets, contributing to the high aggregation capability of the sludge[32,33] (Fig.S13). A lower α-helix/(β-sheet + random coil) ratio suggested that the proteins in the eRAnMBR possessed less hydrogen bonds[35-37] which played a key role in the gel layer[38,39], thus weakening the formation of a compact foulants layer.

Consequently, the eRAnMBR cake layer exhibited a larger BET specific surface area and total pore volume, with porosity reaching 7.47% which was 10 times higher than that of the control group (Fig.S14). ~~which greatly facilitated membrane fouling mitigation and water permeation.~~ The released Mg^{2+} improved the physicochemical properties of sludge flocs making which difficult to deposit or adhere to the membrane surface, thus forming a loose porous cake layer structure, which greatly improved the service life of the membrane.

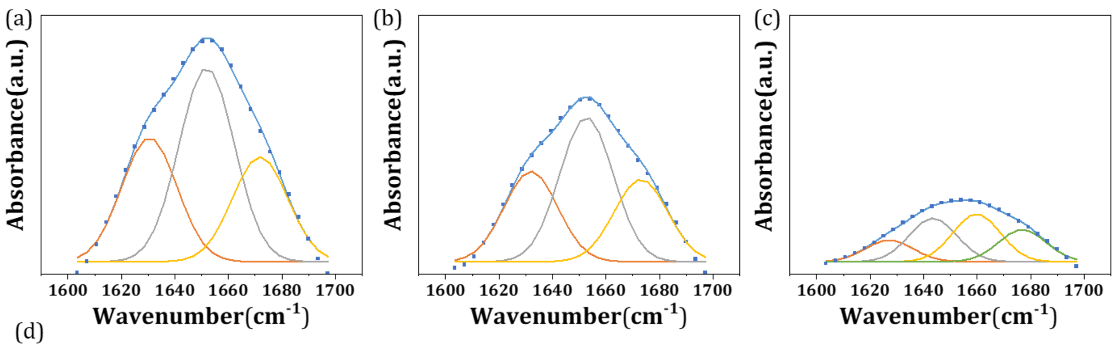

(d)

Sludge	α-helix	β-sheet	Random coil	β-turn	α-Helix/(β-sheet + random coil)
AnMBR	45.56%	28.49%	0.00%	25.96%	1.60
eAnMBR	45.89%	29.30%	0.00%	24.80%	1.57
eRAnMBR	32.97%	14.95%	29.96%	22.13%	0.73

Fig.S13 Second-derivative resolution enhancement and curve-fitted amide I region (1700–1600 cm^{-1}) for EPS proteins from the (a) AnMBR, (b) eAnMBR, and (c) eRAnMBR; (d) Relative content of each secondary structure in EPS proteins for the AnMBR, eAnMBR, and eRAnMBR.

Reviewer (Comment 5): *Section "Carbon, nitrogen, and phosphorus recovery in eRAnMBR": "And from the Energy-dispersive X-ray spectroscopy (EDS) spectrogram as well as the atom proportion, we can draw a conclusion that except struvite there may be a small amount of other Mg phosphate phase generating on the cathode surface." What does this sentence mean here? The author should clarify the research focus of this paragraph.*

Our Response: Thanks for your reminder. This sentence was removed and relevant data on the proportion of struvite was supplemented. We understand that the focus should be on the core content of the paragraph, and that data results should serve the main research points rather than simply being presented. This paragraph is meant to confirm the deposition of struvite on the cathode plate. The statement about the EDS results showing the production of other Mg phosphate compounds besides struvite was not appropriate in this context.

-line 186 Calculating through the method of determination of the struvite on the cathode surface, the purity of struvite was approximately 60.64% and the mass was 0.08 g/d, which meant approximately 9.04% struvite generated in the reactor was deposited on the graphite cathode surface (Fig.S11). ~~And from the Energy-dispersive X-ray spectroscopy (EDS) spectrogram as well as the atom proportion, we can draw a conclusion that except struvite there may be a small amount of other Mg phosphate phase generating on the cathode surface.~~

Reviewer (Comment 6): *Write the future research perspectives in conclusions section. The present information is VERY short and more inclined towards lab studies. The entire world is now shifting from lab to pilot scale works and its important that the authors look beyond the lab for future applications.*

Our Response: Thank you for your kind suggestion. We agree that it is important to look beyond the lab for future applications. Therefore, the conclusion was divided into two sections: one focusing on summarizing the existing results and the other on future prospects. The latter included the significant role of altering the physicochemical properties of sludge in mitigating membrane fouling, the trend of resource recovery in sustainable water treatment when using AnMBR, and the considerations that should be taken into account when applying this novel reactor in practical scenarios.

-line 436 Discussion

In this study, it was found that alleviating membrane fouling by regulating the cake layer through altering the physicochemical properties of the sludge was far more effective than the singular electrodynamic effect. This provided a new approach for extending membrane lifespan in future applications. The new configuration, which switched between the membrane and Mg anodes, reduced Mg material input while effectively recovering three types of resources, enabling the application of AnMBRs in a more sustainable wastewater treatment. As for industrial applications, more economical anode materials, electrode configurations that highly facilitate mass transfer and struvite precipitation, and more efficient utilization of the generated CH_4 to reduce costs are engineering considerations for the practical application of this reactor.

Reviewer (Moderate comments, Comment 1): *Figures and tables should be referenced within the text to guide the reader through the data presentation.*

Our Response: Thanks for your kind recommendation. We have checked all the figures and tables to ensure that they were at the corresponding content in the text

Reviewer (Moderate comments, Comment 2): *More information should be included in Introduction to underscore the significance of this research.*

Our Response: Thanks for your valuable suggestion. The introduction was revised to better highlight the core content of this paper. We clarified the important role of resource and energy recovery in wastewater treatment within the context of sustainable development, and highlighted the promising application prospects of the AnMBRs. However, AnMBRs face challenges such as severe membrane fouling, inability in nutrient recovery, and the need to improve the bioenergy content. We employed the method of electric field to mitigate membrane fouling, focusing not solely on the electrodynamic effects but more on the changes in the physicochemical properties of the sludge and the regulation of the cake layer. Typically, struvite precipitation is used as a post-treatment process following biological treatment, and its application within the bioreactor is rare which faces the problem of the separation of struvite and sludge. The Mg anode was introduced into the AnMBR to achieve simultaneous carbon, nitrogen, and phosphorus recovery,

and mechanisms such as the changes in the physicochemical properties of the sludge and microbial electron transfer within the reactor were further investigated.

-line 37 Introduction

In the context of sustainable development, sewage treatment facilities are not only required to recover renewable resources but also achieve carbon emission reduction. Anaerobic membrane bioreactors (AnMBRs), which integrate anaerobic processes with membrane separation, are among the most effective low-carbon wastewater treatment technologies, offering excellent application prospects in sustainable water treatment. However, challenges such as membrane fouling[1,2] and the inability to recover nitrogen and phosphorus[3,4] significantly restrict the large-scale application of AnMBRs in wastewater treatment[5]. Addressing these issues could greatly enhance their efficiency and application scope[6]. Besides, biogas produced by anaerobic digestion processes plays a key role in the emerging market for renewable energy[7]. However, in addition to 60–70% methane (CH_4), raw biogas contains 30–40% carbon dioxide (CO_2) which decreases its specific calorific value[8]. Therefore, the ratio of CH_4 to CO_2 needs to be increased for biogas to work as an effective fuel source[9]. ~~Furthermore, optimizing biogas production and upgrade to harness this renewable energy source more effectively is a critical future direction for anerobic process, thus supporting the goal of sustainable wastewater development and treatment.~~

~~Various methods for controlling membrane fouling have been reported, including biogas sparging, backwashing, acid cleaning, and bacterial quorum quenching10.~~ Numerous methods have been employed to resist the membrane fouling[10]. Recently, a novel approach that incorporates an electric field in AnMBRs has garnered considerable attention as a chemical-free and efficient way to alleviate *in-situ* membrane pollution[11,12]~~, avoiding the use of chemical agents and preventing secondary pollution~~. The electrostatic repulsion at the cathode effectively inhibited the deposition of negatively charged extracellular polymeric substances (EPS) and soluble microbial products (SMP), which are the main substances contributing to membrane fouling. Most studies have focused on the electrophoresis, electrostatic forces, and other electrodynamic effects of the electric field on the membrane fouling[11-13], while research on the mechanisms of the regulation of the cake layer in AnMBRs is relatively scarce. However, altering the physicochemical properties of the sludge can directly influence the substances that form membrane fouling, thus modifying the structure of the cake layer or slowing its formation.

-line 69 Introduction (continued from the previous page)

As for nitrogen and phosphorus removal, struvite precipitation is considered an excellent method, with recovered struvite further serving as a slow-release fertilizer[14]. Among the various struvite precipitation methods, electrochemical processes are considered promising due to their high efficiency in nitrogen and phosphorus removal[15,16], coupled with the advantages of simple operation, controllable reactions, and reduced sludge production[17,18].~~ The electrochemical method mitigates membrane fouling via electrocoagulation, electrophoresis, and electrochemical oxidation[11-13]. Struvite generation utilizes sacrificial magnesium (Mg) anodes, where anode-released Mg ions (Mg^{2+}) react with ammonium (NH_4^+) and phosphate (PO_4^{3-}) ions in bulk solution to form struvite[19].~~ Typically, struvite precipitation is used as a post-treatment process following biological treatment[15,16], and its application within the bioreactor is rare which faces the problem of the separation of struvite and sludge. If Mg anode was introduced into the AnMBR system, it could exert dual effects of the nitrogen and phosphorus removal and electrochemical regulation on membrane fouling. Additionally, the Mg^{2+} ions and hydroxide ions (OH^-) produced from the hydrogen evolution reaction at the cathode can absorb CO_2 produced by anaerobic digestion, synergizing CO_2 mineralization and biogas upgrading[20]. Understanding how the Mg anode impacts the biological system during this process is a key area that warrants further investigation.~~ Therefore, the electrochemical process enables simultaneous membrane antifouling and carbon, nitrogen, and phosphorus recovery. To date, however, few studies have explored these challenges and improved biogas performance within a single reactor.~~

In this study, a novel electrochemical resource-recovery AnMBR (eRAnMBR) equipped with double anodes, consisting of an Mg plate and conductive membrane-anode, was developed to facilitate nutrient and energy recovery, as well as alleviate membrane fouling by unblocking the cake layer. Over a 200-day operational period, the eRAnMBR enhanced the recovery of carbon, nitrogen, and phosphorus and improved membrane filtration capacity. ~~Proteins were more likely to participate in redox reactions, accelerating the electron transport rate following the imposition of anodic potential on the membrane.~~ The introduction of the Mg anode improved struvite generation and enhanced the absorption of CO_2 from the biogas into the sludge-water mixture, ~~while also alleviating membrane fouling by increasing particle size, strengthening hydrophobicity, and reducing the viscosity of the sludge flocs~~ while also altering the structure of the cake layer and inhibiting the formation of the gel layer. The introduction of the electric field enhanced the microbial anodic oxidation and accelerated the transformation of proteins within the system. ~~Additionally, an increase in *Methanosarcina* archaea within the eRAnMBR facilitated more effective interspecific electron transfer and membrane fouling mitigation.~~ Changes in the archaea community and the increased capacity for DIET within the system were also the reason for the promotion of CH_4 production. This study presents a novel strategy for resource and energy recovery from sewage, demonstrating considerable potential for efficient and eco-friendly wastewater treatment.

Reviewer (Moderate comments, Comment 3): *There appears to be a lack of a coherent logical connection between the various sections of the paper. It is necessary for the author to improve it.*

Our Response: Thanks for your kind recommendation. We should improve the logical coherence of the paper, so sentences that provide coherence between sections were added to enhance the logical flow.

-Line 158 The eRAnMBR also demonstrated considerable nitrogen and phosphorus resource recovery. As shown in Fig.2a, ammonia nitrogen (NH_4^+) and phosphate (PO_4^{3-}) significantly decreased in the eRAnMBR, with removal rates of 30.59% and 99%, respectively, compared to minimal recovery in the controlled AnMBR and eAnMBR.

-Line 203 ~~Membrane fouling in the eRAnMBR was significantly alleviated due to improvements in the physical and chemical properties of the sludge floc.~~ Membrane fouling was also significantly alleviated in the eRAnMBR.

-Line 284 The mechanisms behind the aforementioned results were further investigated. The cyclic voltammogram (CV) curves in Fig. 4b showed that the sludge cake layer on the membrane-anode exhibited markedly higher electron-storage capacity and peak oxidation current compared to the control membrane, indicating the enhancement of microbial anodic oxidation.

-Line 310 Moreover, the quantity of slime, loosely-bound EPS (LB-EPS), tightly-bound EPS (TB-EPS) of sludge around the membrane in the three reactors was also calculated to further investigate the effect of electric field on organic matter forming membrane fouling.

-Line 364 The analysis of archaeal community also explained the mechanism behind enhanced CH_4 production of the eRAnMBR.

Reviewer (Moderate comments, Comment 4): *The section of method in the manuscript is quite lengthy, but there are still some details of the methods that are not clearly written. It is necessary for the author to supplement or move to SI.*

Our Response: Thanks for your valuable advice. More details of the methods were added to make the paper more comprehensive and rigorous, and some of the more detailed methods were moved to the supplementary information to avoid making the main article overly lengthy. The method of Preliminary operating cost analysis for the eRAnMBR (Text.S3) and EPS extraction (Text.S4) were put into SI, and supplement the method of 2DCOS and Metagenomic analysis in the method.

-Line 563 BLASTP v2.2.28+ (http://blast.ncbi.nlm.nih.gov/Blast.cgi) was used to compare non-redundant gene sequences with the Kyoto Encyclopedia of Genes and Genomes (KEGG) database, with the expected value of BLAST comparison parameters set to $1e^{-5}$. Based on the comparative results, functional annotation was conducted using KOBAS 2.0 [4] (KEGG Orthology Based Annotation System). The abundance of genes corresponding to KO, Pathway, EC, and Module was summed to calculate the abundance of this functional category.

-line 495 All extracted EPS samples were measured using a three-dimensional excitation emission matrix (EEM) (F-4600, HITACHI, Japan) and Fourier transform infrared spectroscopy (FTIR) (Nicolet iN10MX, Thermo Fisher Scientific, USA). The software 2D Shige ver. 1.3 (Shigeaki Morita, Kwansei-Gakuin University, Japan) was used to analyze the infrared spectra data of LB-EPS and SMP, and then obtained the 2D-COS spectra to gain insights into the molecular compositions and formation mechanisms of organic substances.

Text S3. Preliminary operating cost analysis for eAnRMBR

In this system, many sources of the electric energy consumption (e.g. the pump, the constant temperature water bath and the mixer) and operation costs (e.g.pipeline and membrane replacement) were consistent between the eRAnMBR and conventional AnMBR. Therefore, the additional operating cost of the eRAnMBR compared to conventional AnMBR was composed of energy consumption (electricity) and material consumption (electrode) which drove the electrochemical processes. It was estimated using the following equation.

$$\mathrm{C} = \mathrm{aENC} + \mathrm{bELC} \quad (1)$$

Where, C refers to the additional operating cost, USD/m^3; ENC is the energy consumption, kWh/m^3; ELC is the electrode consumption, kg/ m^3; a is the unit cost of electricity, i.e. 0.102 USD/kWh[1]; and b is the unit cost of the magnesium (Mg) electrode, i.e. 2.56 USD/kg (Because graphite plates are cheap and can be reused during the experiment, we only consider Mg electrode here).

In this system, although additional electrical energy is consumed, the internal methane produced is also a form of energy. Therefore, ENC was calculated as follows:

$$ENC = W_e - W_b \quad (2)$$

The electric energy consumption (W_e, Wh·m^{-3}) was calculated as follows:

$$W_e = \frac{UIt}{3600V} = \frac{UI}{3600Q} \quad (3)$$

Methane was the internal capacity of AnMBR. Under standard conditions, gas production (P_2, Nm^3) was calculated as follows:

$$\frac{P_1V_1}{T_1} = \frac{P_2V_2}{T_2} \quad (4)$$

Biogas energy (methane to electricity, W_b, Wh·m^{-3}) was calculated as follows:

$$W_b = \frac{\varepsilon q V_2 t}{3600V} = \frac{\varepsilon q V_2}{3600Q} \quad (5)$$

where U is the applied potential (V), I is the current (A), t is the running time (h), Q is the flow (m^3 • h^{-1}), P_1 is the working pressure (atm), V_1 is the working biogas production rate (m^3 • h^{-1}), T_1 is 298 K (25 °C), P_2 is one standard atmospheric pressure (atm), V_2 is the standard gas production (Nm^3 • h^{-1}), T_2 is 273 K (0 °C), q is the heat value of the biogas (J • Nm^{-3}), and ε (%) is the methane-to-electricity conversion efficiency with a typical value of 13.779% via recent methane fuel cells[2].

While, the electrode consumption was determined following Faraday's Law[3] as expressed in Eq. (6).

$$\mathrm{ELC} = \frac{\mathrm{IMt}}{\mathrm{nFV}} \quad (6)$$

Where, I is the applied current, A; t is the electrolysis time, h; V is the volume of treated effluent, m^3; M is the molecular weight of iron electrode, g/mol; n is the number of transferred electrons; and F is the Faraday constant, coulomb/mol.

Text S4. The method of extracellular polymeric substances (EPS) extraction

Extracellular polymeric substances (EPS) were extracted by thermal extraction[4]. In brief, 25 mL of sludge-water mixture collected from the AnMBRs was first centrifuged at 4 000 *g* and 4 °C for 5 min, with the collected supernatant representing the slime layer. The sludge remaining in the centrifuge tube was then added to 25 mL of 0.05% NaCl solution for re-suspension, sheared for 1 min using a vortex mixer, and centrifuged again at 4 000 *g* for 10 mins and 4 °C, with the collected supernatant representing LB-EPS. Subsequently, 25 mL of 0.05% NaCl solution was added to re-suspend the sludge left in the centrifuge tube, followed by a water bath at 60 °C for 30 min and centrifugation at 4 000 *g* for 15 min and 4 °C, with the collected supernatant representing TB-EPS.

References

1. Sanders, C., Turkarslan, S., Lee, D.W., and Daldal, F., *Cytochrome c biogenesis: the Ccm system.* Trends in Microbiology, 2010. **18**(6): 266-274.
2. Rotaru, A.E., Shrestha Pravin, M., Liu, F., Markovaite, B., Chen, S., Nevin Kelly, P., and Lovley Derek, R., *Direct Interspecies Electron Transfer between Geobacter metallireducens and Methanosarcina barkeri.* Applied and Environmental Microbiology, 2014. **80**(15): 4599-4605.
3. McGlynn, S.E., Chadwick, G.L., Kempes, C.P., and Orphan, V.J., *Single cell activity reveals direct electron transfer in methanotrophic consortia.* Nature, 2015. **526**(7574): 531-535.
4. Chen, M., Li, Y.L., Sun, X.Y., Dai, R.B., Zheng, J.J., Wang, X., and Wang, Z.W., *Recent advances in electrochemical processes integrated with anaerobic membrane bioreactor in wastewater treatment.* Chemical Engineering Journal, 2023. **468**.
5. Andronikou, M., Adamou, V., Koutsokeras, L., Constantinides, G., and Vyrides, I., *Magnesium ribbon and anaerobic granular sludge for conversion of CO2 to CH4 or biogas upgrading.* Chemical Engineering Journal, 2022. **435**: 134888.
6. Belmabkhout, Y., Bhatt, P.M., Adil, K., Pillai, R.S., Cadiau, A., Shkurenko, A., Maurin, G., Liu, G., Koros, W.J., and Eddaoudi, M., *Natural gas upgrading using a fluorinated MOF with tuned H2S and CO2 adsorption selectivity.* Nature Energy, 2018. **3**(12): 1059-1066.
7. He, Q.Y., Yu, G., Tu, T., Yan, S.P., Zhang, Y.L., and Zhao, S.F., *Closing CO2 Loop in Biogas Production: Recycling Ammonia As Fertilizer.* Environmental Science & Technology, 2017. **51**(15): 8841-8850.
8. Liu, C.Q., Xiao, J.W., Li, H.Y., Chen, Q., Sun, D.Z., Cheng, X., Li, P.S., Dang, Y., Smith, J.A., and Holmes, D.E., *High efficiency in-situ biogas upgrading in a bioelectrochemical system with low energy input.* Water Research, 2021. **197**: 117055.
9. Lim, K., Evans, P.J., and Parameswaran, P., *Long-Term Performance of a Pilot-Scale Gas-Sparged Anaerobic Membrane Bioreactor under Ambient Temperatures for Holistic Wastewater Treatment.* Environmental Science & Technology, 2019. **53**(13): 7347-7354.
10. Cheng, D.L., Ngo, H.H., Guo, W.S., Liu, Y.w., Chang, S.W., Nguyen, D.D., Nghiem, L.D., Zhou, J.L., and Ni, B.J., *Anaerobic membrane bioreactors for antibiotic wastewater*

treatment: Performance and membrane fouling issues. Bioresource Technology, 2018. **267**: 714-724.

11. Hu, Z.T., Li, L.Q., Cen, X.T., Zheng, M., Hu, S.H., Wang, X.H., Song, Y.R., Xu, K.N., and Yuan, Z.G., *Integrated urban water management by coupling iron salt production and application with biogas upgrading.* Nature Communications, 2023. **14**(1): 6405.
12. Katuri, K.P., Werner, C.M., Jimenez-Sandoval, R.J., Chen, W., Jeon, S., Logan, B.E., Lai, Z., Amy, G.L., and Saikaly, P.E., *A Novel Anaerobic Electrochemical Membrane Bioreactor (AnEMBR) with Conductive Hollow-fiber Membrane for Treatment of Low-Organic Strength Solutions.* Environmental Science & Technology, 2014. **48**(21): 12833-12841.
13. Meng, F.G., Zhang, S.Q., Oh, Y.t., Zhou, Z.B., Shin, H.S., and Chae, S.R., *Fouling in membrane bioreactors: An updated review.* Water Research, 2017. **114**: 151-180.
14. Yuan, H.P. and Zhu, N.W., *Progress in inhibition mechanisms and process control of intermediates and by-products in sewage sludge anaerobic digestion.* Renewable and Sustainable Energy Reviews, 2016. **58**: 429-438.
15. Jiang, J.G., Zhang, Y.J., Li, K.M., Wang, Q., Gong, C.X., and Li, M.L., *Volatile fatty acids production from food waste: Effects of pH, temperature, and organic loading rate.* Bioresource Technology, 2013. **143**: 525-530.
16. Zhang, W.L., Xing, W.L., and Li, R.D., *Real-time recovery strategies for volatile fatty acid-inhibited anaerobic digestion of food waste for methane production.* Bioresource Technology, 2018. **265**: 82-92.
17. Yuan, Y.Y., Hu, X.Y., Chen, H.B., Zhou, Y.Y., Zhou, Y.F., and Wang, D.B., *Advances in enhanced volatile fatty acid production from anaerobic fermentation of waste activated sludge.* Science of The Total Environment, 2019. **694**: 133741.
18. Cotter Paul, D. and Hill, C., *Surviving the Acid Test: Responses of Gram-Positive Bacteria to Low pH.* Microbiology and Molecular Biology Reviews, 2003. **67**(3): 429-453.
19. Munoz, R., Meier, L., Diaz, I., and Jeison, D., *A review on the state-of-the-art of physical/chemical and biological technologies for biogas upgrading.* Reviews in Environmental Science and Bio-Technology, 2015. **14**(4): 727-759.
20. Sun, Q., Li, H.L., Yan, J.Y., Liu, L.C., Yu, Z.X., and Yu, X.H., *Selection of appropriate biogas upgrading technology-a review of biogas cleaning, upgrading and utilisation.* Renewable and Sustainable Energy Reviews, 2015. **51**: 521-532.
21. Miltner, M., Makaruk, A., and Harasek, M., *Review on available biogas upgrading technologies and innovations towards advanced solutions.* Journal of Cleaner Production, 2017. **161**: 1329-1337.
22. Makaruk, A., Miltner, M., and Harasek, M., *Membrane biogas upgrading processes for the production of natural gas substitute.* Separation and Purification Technology, 2010. **74**(1): 83-92.
23. Khoiruddin, K., Ursada, R., Warmadewanthi, I.D.A.A., Bagastyo, A.Y., Anggrainy, A.D., and Wenten, I.G., *Electrochemically-driven struvite recovery: Prospect and challenges for the application of magnesium sacrificial anode.* Separation and Purification Technology, 2022. **288**.
24. Kato, S., Yumoto, I., and Kamagata, Y., *Isolation of Acetogenic Bacteria That Induce Biocorrosion by Utilizing Metallic Iron as the Sole Electron Donor.* Applied and Environmental Microbiology, 2015. **81**(1): 67-73.

25. Huang, H.M., Zhang, P., Zhang, Z., Liu, J.H., Xiao, J., and Gao, F.M., *Simultaneous removal of ammonia nitrogen and recovery of phosphate from swine wastewater by struvite electrochemical precipitation and recycling technology.* Journal of Cleaner Production, 2016. **127**: 302-310.
26. Cao, F.Y., Shi, Z.M., Hofstetter, J., Uggowitzer, P.J., Song, G.L., Liu, M., and Atrens, A., *Corrosion of ultra-high-purity Mg in 3.5% NaCl solution saturated with Mg(OH)2.* Corrosion Science, 2013. **75**: 78-99.
27. Thomas, S., Medhekar, N.V., Frankel, G.S., and Birbilis, N., *Corrosion mechanism and hydrogen evolution on Mg.* Current Opinion in Solid State and Materials Science, 2015. **19**(2): 85-94.
28. Qu, Q., Wang, L., Li, L., He, Y., Yang, M., and Ding, Z.T., *Effect of the fungus, Aspergillus niger, on the corrosion behaviour of AZ31B magnesium alloy in artificial seawater.* Corrosion Science, 2015. **98**: 249-259.
29. Qu, Q., Li, S.L., Li, L., Zuo, L.M., Ran, X., Qu, Y., and Zhu, B.L., *Adsorption and corrosion behaviour of Trichoderma harzianum for AZ31B magnesium alloy in artificial seawater.* Corrosion Science, 2017. **118**: 12-23.
30. Hug, A. and Udert, K.M., *Struvite precipitation from urine with electrochemical magnesium dosage.* Water Research, 2013. **47**(1): 289-299.
31. Kékedy-Nagy, L., Teymouri, A., Herring, A.M., and Greenlee, L.F., *Electrochemical removal and recovery of phosphorus as struvite in an acidic environment using pure magnesium vs. the AZ31 magnesium alloy as the anode.* Chemical Engineering Journal, 2020. **380**: 122480.
32. Xu, J., Sheng, G.P., Ma, Y., Wang, L.F., and Yu, H.Q., *Roles of extracellular polymeric substances (EPS) in the migration and removal of sulfamethazine in activated sludge system.* Water Research, 2013. **47**(14): 5298-5306.
33. Jiang, Z.H., Yu, Q.L., Sun, C., Wang, Z.X., Jin, Z., Zhu, Y.H., Zhao, Z.Q., and Zhang, Y.B., *Additional electric field alleviates acidity suppression in anaerobic digestion of kitchen wastes via enriching electro-active methanogens in cathodic biofilms.* Water Research, 2022. **212**: 118118.
34. Hou, R., Luo, C., Zhou, S.F., Wang, Y., Yuan, Y., and Zhou, S.G., *Anode potential-dependent protection of electroactive biofilms against metal ion shock via regulating extracellular polymeric substances.* Water Research, 2020. **178**: 115845.
35. Yu, Q.L. and Zhang, Y.B., *Fouling-resistant biofilter of an anaerobic electrochemical membrane reactor.* Nature Communications, 2019. **10**(1): 4860.
36. Jiang, Z.H., Yu, Q., Zhao, Z.Q., Song, X.Y., and Zhang, Y.B., *Reason for the increased electroactivity of extracellular polymeric substances with electrical stimulation: Structural change of α-helix peptide of protein.* Water Research, 2023. **238**: 119995.
37. Yang, G.Q., Huang, L.Y., Yu, Z., Liu, X.M., Chen, S.S., Zeng, J.X., Zhou, S.G., and Zhuang, L., *Anode potentials regulate Geobacter biofilms: New insights from the composition and spatial structure of extracellular polymeric substances.* Water Research, 2019. **159**: 294-301.
38. Lee, D.-y.D., Prindle, A., Liu, J.T., and Suel, G.M., *SnapShot: Electrochemical Communication in Biofilms.* Cell, 2017. **170**(1).
39. Li, S.W., Sheng, G.P., Cheng, Y.Y., and Yu, H.Q., *Redox properties of extracellular*

polymeric substances (EPS) from electroactive bacteria. Scientific Reports, 2016. **6**(1): 39098.

40. Xu, H., He, E.k., Peijnenburg, W.J.G.M., Song, L., Zhao, L., Xu, X.Y., Cao, X.D., and Qiu, H., *Contribution of pristine and reduced microbial extracellular polymeric substances of different sources to Cu(II) reduction.* Journal of Hazardous Materials, 2021. **415**: 125616.
41. Udomkittayachai, N., Xue, W.C., Xiao, K., Visvanathan, C., and Tabucanon, A.S., *Electroconductive moving bed membrane bioreactor (EcMB-MBR) for single-step decentralized wastewater treatment: Performance, mechanisms, and cost.* Water Research, 2021. **188**: 116547.
42. Ying, X.B., Huang, J.J., Shen, D.S., Feng, H.J., Jia, Y.F., and Guo, Q.Q., *Fouling behaviors are different at various negative potentials in electrochemical anaerobic membrane bioreactors with conductive ceramic membranes.* Science of The Total Environment, 2021. **761**: 143199.
43. Juntawang, C., Rongsayamanont, C., and Khan, E., *Entrapped cells-based-anaerobic membrane bioreactor treating domestic wastewater: Performances, fouling, and bacterial community structure.* Chemosphere, 2017. **187**: 147-155.
44. Chen, Y.F., Teng, J.H., Shen, L.G., Yu, G.Y., Li, R.J., Xu, Y.C., Wang, F.Y., Liao, B.Q., and Lin, H.J., *Novel insights into membrane fouling caused by gel layer in a membrane bioreactor: Effects of hydrogen bonding.* Bioresource Technology, 2019. **276**: 219-225.
45. Lei, Z., Wang, J., Leng, L.W., Yang, S.M., Dzakpasu, M., Li, Q., Li, Y.Y., Wang, X.C.C., and Chen, R., *New insight into the membrane fouling of anaerobic membrane bioreactors treating sewage: Physicochemical and biological characterization of cake and gel layers.* Journal of Membrane Science, 2021. **632**: 119383.
46. Chen, J.R., Zhang, M.J., Li, F.Q., Qian, L., Lin, H.J., Yang, L.N., Wu, X.L., Zhou, X.L., He, Y.M., and Liao, B.Q., *Membrane fouling in a membrane bioreactor: High filtration resistance of gel layer and its underlying mechanism.* Water Research, 2016. **102**: 82-89.
47. Wang, X.M. and Li, X.Y., *Accumulation of biopolymer clusters in a submerged membrane bioreactor and its effect on membrane fouling.* Water Research, 2008. **42**(4): 855-862.
48. Udomkittayachai, N., Xue, W., Xiao, K., Visvanathan, C., and Tabucanon, A.S., *Electroconductive moving bed membrane bioreactor (EcMB-MBR) for single-step decentralized wastewater treatment: Performance, mechanisms, and cost.* Water Research, 2021. **188**: 116547.
49. Wang, Y.C., Kuntke, P., Saakes, M., van der Weijden, R.D., Buisman, C.J.N., and Lei, Y., *Electrochemically mediated precipitation of phosphate minerals for phosphorus removal and recovery: Progress and perspective.* Water Research, 2022. **209**: 117891.
50. Zhao, Z.Q., Zhang, Y.B., Yu, Q.L., Dang, Y., Li, Y., and Quan, X., *Communities stimulated with ethanol to perform direct interspecies electron transfer for syntrophic metabolism of propionate and butyrate.* Water Research, 2016. **102**: 475-484.
51. Xiao, Y., Zhang, E.H., Zhang, J.D., Dai, Y.F., Yang, Z.H., Christensen, H.E.M., Ulstrup, J., and Zhao, F., *Extracellular polymeric substances are transient media for microbial extracellular electron transfer.* Science Advances. **3**(7): e1700623.
52. Prindle, A., Liu, J., Asally, M., Ly, S., Garcia-Ojalvo, J., and Süel, G.M., *Ion channels enable electrical communication in bacterial communities.* Nature, 2015. **527**(7576): 59-63.

53. Ki, D., Popat, S.C., Rittmann, B.E., and Torres, C.I., *H2O2 Production in Microbial Electrochemical Cells Fed with Primary Sludge.* Environmental Science & Technology, 2017. **51**(11): 6139-6145.
54. Yu, Q.L., Jin, X.C., and Zhang, Y.B., *Sequential pretreatment for cell disintegration of municipal sludge in a neutral Bio-electro-Fenton system.* Water Research, 2018. **135**: 44-56.
55. Zhang, H.M., Jiang, W., and Cui, H.T., *Performance of anaerobic forward osmosis membrane bioreactor coupled with microbial electrolysis cell (AnOMEBR) for energy recovery and membrane fouling alleviation.* Chemical Engineering Journal, 2017. **321**: 375-383.
56. Hou, X., Liu, S., and Zhang, Z., *Role of extracellular polymeric substance in determining the high aggregation ability of anammox sludge.* Water Research, 2015. **75**: 51-62.
57. Tamate, R., Peng, Y., Kamiyama, Y., and Nishikawa, K., *Extremely Tough, Stretchable Gel Electrolytes with Strong Interpolymer Hydrogen Bonding Prepared Using Concentrated Electrolytes to Stabilize Lithium-Metal Anodes.* Advanced Materials, 2023. **35**(22): 2211679.

REVIEWERS' COMMENTS

Reviewer #1 (Remarks to the Author):

I have confirmed that the manuscript has been modified and improved based on my previous comments.

Our Response: Thank you very much for your valuable feedback, which has allowed us to improve our paper.

Reviewer #2 (Remarks to the Author):

Authors have reivsed the manuscript and it can be acceptable.

Our Response: Thank you very much for your trust.

Reviewer #3 (Remarks to the Author):

I have reviewed the revised manuscript and the response, and think the comments have been well addressed by the authors. I suggest the manuscript can be accepted in the present form.

Our Response: Thank you very much for your help and guidance.
